# T and B cell responses against Epstein–Barr virus in primary sclerosing cholangitis

**DOI:** 10.1038/s41591-025-03692-w

**Published:** 2025-06-11

**Authors:** Hesham ElAbd, Mitchell Pesesky, Gabriel Innocenti, Brian K. Chung, Aya K. H. Mahdy, Valeriia Kriukova, Laila Kulsvehagen, Dennis Strobbe, Claudia Stühler, Gabriele Mayr, Damon H. May, Melanie Prinzensteiner, Tim A. Steiert, Florian Tran, Michel V. Hadjihannas, Rainer Günther, Elisa Rosati, Sören Mucha, Wolfgang Lieb, Malte Ziemann, Astrid Dempfle, Felix Braun, Trine Folseraas, Johannes R. Hov, Espen Melum, Petra Bacher, Martina Sterneck, Tobias J. Weismüller, Henrike Lenzen, Bernd Bokemeyer, Bryan Howie, Harlan S. Robins, Christoph Röcken, Stefan Schreiber, Nina Khanna, Anne-Katrin Pröbstel, Christoph Schramm, Thomas Vogl, Tom H. Karlsen, Andre Franke

**Affiliations:** 1 Institute of Clinical Molecular Biology, Kiel University and University Hospital Schleswig-Holstein (UKSH), Kiel, Germany; 2 Adaptive Biotechnologies, Seattle, WA USA; 3 Center for Cancer Research, Medical University of Vienna, Vienna, Austria; 4 Norwegian PSC Research Center, Department of Transplantation Medicine, Division of Surgery and Specialized Medicine Oslo University Hospital Rikshospitalet, Oslo, Norway; 5 Research Institute of Internal Medicine, Oslo University Hospital, Oslo, Norway; 6 Institute of Clinical Medicine, University of Oslo, Oslo, Norway; 7 Departments of Neurology, Biomedicine and Clinical Research, and Research Center for Clinical Neuroimmunology and Neuroscience Basel (RC2NB), University Hospital Basel and University of Basel, Basel, Switzerland; 8 Department of Biomedicine, Infection Biology Laboratory, University and University Hospital Basel, Basel, Switzerland; 9 Department of Internal Medicine I, Division of Hepatology/Transplant Hepatology, University Hospital Schleswig-Holstein, Kiel, Germany; 10 Popgen Biobank and Institute of Epidemiology, University of Kiel, Kiel, Germany; 11 Institute of Transfusion Medicine, University Hospital Schleswig-Holstein, Lübeck/Kiel, Kiel, Germany; 12 Institute of Medical Informatics and Statistics, Kiel University and University Hospital Schleswig-Holstein, Kiel, Germany; 13 Department of General, Visceral-, Thoracic-, Transplant- and Pediatric-Surgery, University Hospital Schleswig-Holstein Campus Kiel, Kiel, Germany; 14 Hybrid Technology Hub-Centre of Excellence, Institute of Basic Medical Sciences, Faculty of Medicine, University of Oslo, Oslo, Norway; 15 Institute of Immunology, University of Kiel, Kiel, Germany; 16 Department of Internal Medicine, University Medical Center Hamburg-Eppendorf, Hamburg, Germany; 17 University Transplant Center, University Medical Center Hamburg-Eppendorf, Hamburg, Germany; 18 Department of Internal Medicine I, University Hospital of Bonn, Bonn, Germany; 19 Department of Gastroenterology, Hepatology and Oncology, Vivantes Humboldt-Hospital, Berlin, Germany; 20 Department of Gastroenterology, Hepatology Infectious Diseases and Endocrinology, Hannover Medical School, Hannover, Germany; 21 Interdisciplinary Crohn Colitis Centre Minden, Minden, Germany; 22 Department of Pathology, University Medical Center Schleswig-Holstein, Kiel, Germany; 23 Division of Infectious Diseases, University and University Hospital Basel, Basel, Switzerland; 24 Center of Neurology, Department of Neuroimmunology, University Hospital and University Bonn, Bonn, Germany; 25 German Center for Neurodegenerative Diseases (DZNE), Bonn, Germany; 26 First Department of Medicine, University Medical Centre Hamburg Eppendorf, Hamburg, Germany; 27 Martin Zeitz Center for Rare Diseases, University Medical Centre Hamburg-Eppendorf, Hamburg, Germany; 28 Hamburg Centre for Translational Immunology (HCTI), University Medical Center Hamburg-Eppendorf, Hamburg, Germany

**Keywords:** Adaptive immunity, Chronic inflammation

## Abstract

Primary sclerosing cholangitis (PSC) is an idiopathic, progressive and incurable liver disease. Here, we aimed for systematic analyses of adaptive immune responses in PSC. By profiling the T cell repertoires of 504 individuals with PSC and 904 healthy controls, we identified 1,008 clonotypes associated with PSC. A substantial fraction of these clonotypes was restricted to known PSC human leukocyte antigen susceptibility alleles and known to target Epstein–Barr virus (EBV) epitopes. We further utilized phage-immunoprecipitation sequencing to determine antibody epitope repertoires of 120 individuals with PSC and 202 healthy controls, which showed a higher burden of anti-EBV responses in PSC than controls. EBV-specific monoclonal antibodies isolated from B cells in PSC livers corroborated convergent B and T cell responses against EBV. By analyzing electronic health records of >116 million people, we identified an association between infectious mononucleosis and PSC (odds ratio, 12; 95% confidence interval, 6.3–22.9), suggesting a link between EBV and PSC.

## Abstract (web summary)

Interrogation of electronic health records and the B and T cell repertoires of people with PSC shows a link between Epstein–Barr virus and primary sclerosing cholangitis, an incurable autoimmune liver disease.

## Main

PSC is characterized by chronic inflammation and progressive fibrosis of the bile ducts leading to bile duct obstruction and liver failure^1^. The lack of effective medical therapy means that liver transplantation is the only therapeutic option available in PSC with end-stage liver disease^1^. Although a rare disease with an incidence rate of ~0.4 to 2.0 in 100,000 per year and a prevalence of 1 per 10,000 in the United States and Northern Europe, up to 10–15% of all liver transplantations in these regions can be attributed to PSC^2,3^, and patient symptom burden and healthcare costs are high^4^. More than 30–40% of people with PSC experience recurrence after liver transplantation despite the ongoing immunosuppression, sometimes requiring a second liver transplant, highlighting the need for a deeper understanding of disease etiology and effective treatments.

Several genetic variants at different loci show robust associations with PSC, for example, the T cell relevant *FOXP1* (ref. ^5^), *NFKB1* (ref. ^6^) and *CD226* (ref. ^7^) genes, in addition to multiple human leukocyte antigen (HLA) alleles, for example, HLA-B*08:01 and HLA-DRB1*13:01 (refs. ^8,9^). Direct evidence for an involvement of T cells has been reported, for example, an oligoclonal expansion of multiple T cell receptor (TCR) clonotypes in the bile duct in PSC was identified previously^10^, indicating chronic antigenic exposure. Furthermore, an expansion of tissue-resident naive-like CD4^+^ T cells in PSC livers has been reported^11^. Liver-infiltrating T cells exhibit functional differences, such as impaired proliferative activities^12^, decreased production of IL-2, increased production of tumor necrosis factor^12^ and a reduction in CD28 surface expression^13^, in PSC compared with controls. However, the antigenic targets of these liver-infiltrating T cells remain unknown.

TCRs govern the antigenic specificity of T cells and are generated through a somatic recombination process known as V(D)J recombination^14^ that creates a diverse repertoire of clonotypes, enabling T cells to recognize a wide range of antigens. Although recent advances in next-generation sequencing have enabled reliable identification of clonotypes^15,16^, unraveling the corresponding antigen specificity remains difficult. Nonetheless, by comparing the TCR beta (TRB) repertoires of cytomegalovirus (CMV) seropositive and seronegative people statistically, clonotypes associated with CMV infection were identified^17^. The same framework was used to identify clonotypes associated with SARS-CoV-2 exposure^18^ and Lyme disease^19^. TCR profiling was also employed to discover clonotypes associated with chronic inflammatory diseases such as ankylosing spondylitis^20,21^, enabling therapeutic strategies that deplete disease-associated clones and seem to introduce clinical remission in these patients^22^.

Here, we profiled the TRB repertoires of people with PSC using TCR sequencing (TCR-seq), which, relative to controls, showed distinct repertoire features, primarily expanded immune responses against several EBV epitopes. We then applied phage-immunoprecipitation sequencing (PhIP–seq)^23,24^ to profile antibody responses (from (1) blood and (2) monoclonal antibodies isolated from livers of people with PSC) against 357,000 bacterial and viral antigens, demonstrating converging expanded responses against EBV antigens in PSC.

## Results

### Identifying PSC-associated TRB clonotypes

**Fig. 1:**
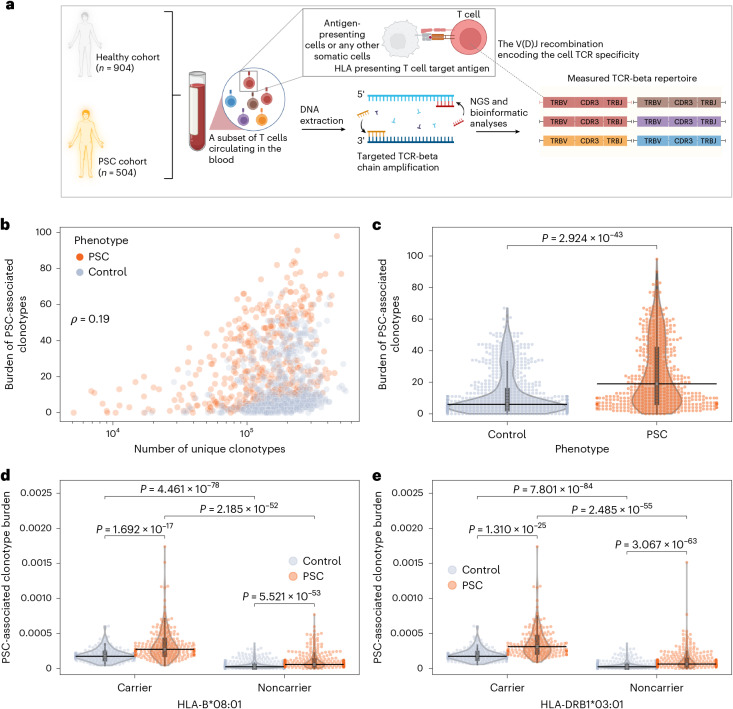
Burden of PSC-associated meta-clonotypes and associations with alleles of the ancestral HLA haplotype 8.1. **a**, Schematic overview of the TCR sequencing (TCR-seq) pipeline used, starting with the collection of peripheral blood, followed by DNA extraction, then targeted amplification using PCR and then sequencing. Finally, different bioinformatic pipelines were used to identify T cell clonotypes and to quantify their expansion from the generated sequencing reads. **b**, Weak correlation between the burden of primary sclerosing cholangitis (PSC)-associated meta-clonotypes (*n* = 1,008 clonotypes) and repertoire size as measured by Spearman correlation (ρ) across all repertoires (*n* = 1,408 people). Burden is defined as the number of unique PSC-associated clonotypes identified in the repertoire of each person (*y* axis). The *x* axis shows the total number of unique clonotypes, defined as unique V and J genes combinations in addition to the amino acid sequence of the CDR3 region. **c**, Average difference in the burden of PSC-associated meta-clonotypes in people with PSC (*n* = 504) and controls (*n* = 904). **d**, Normalized burden in HLA-B*08:01 carrier individuals with (*n* = 205) and without (*n* = 167) PSC relative to noncarrier individuals with (*n* = 299) and without (*n* = 737) PSC. **e**, The same observations for HLA-DRB1*03:01 carrier individuals with (*n* = 179) and without (*n* = 173) PSC in comparison with noncarrier individuals with (*n* = 325) and without (*n* = 731) PSC. In **b**–**e** each dot represents the repertoire of an individual with or without PSC; black lines represent the median across different individuals; boxplot, interquartile range (IQR); whiskers represent datapoints within the 1.5 times the IQR range. In **c**–**e**, a two-sided Mann–Whitney–Wilcoxon test was used to compare the burden or the normalized burden between the different groups included in the study. Panel **a** was created using BioRender.com.

Given the strong evidence for involvement of T cells in the pathogenesis of PSC, we profiled the TRB repertoires (Fig. 1a; Methods) of 504 individuals with PSC and 904 sex- and age-matched healthy controls (Extended Data Table 1). To identify PSC-associated clonotypes in a hypothesis-free manner, we compared their TRB repertoires with that of healthy controls (Methods)^17^, resulting in the identification of 136 clonotypes associated with PSC (Supplementary Table 1). We next expanded these 136 clonotypes into PSC-associated ‘meta-clones’ defined as a collection of highly similar clonotypes associated with PSC using seeded clustering (Methods). This enabled us to identify 1,008 PSC-associated clonotypes (Supplementary Table 2) arranged into 136 meta-clonotypes.

**Extended Data Fig. 1:**
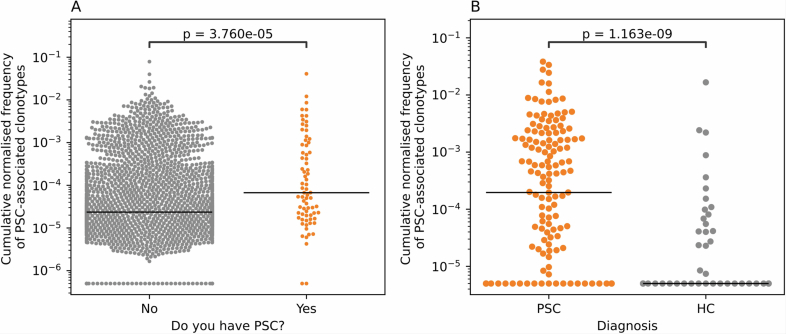
The expansion of the 1,008 PSC-associated clonotypes identified in the current study in two independent validation cohorts. (**a**) The expansion of the PSC-associated clonotypes in the US-based SPARC IBD cohort containing 2,487 individuals with IBD and 73 individuals with PSC-IBD^25^. (**b**) The expansion of PSC-associated clonotypes in 154 individuals with PSC and 64 healthy controls from Norway. Each dot represents the cumulative expansion of all PSC-associated clonotypes in the repertoire of an individual, and the black lines represent the median. In both panels, a two-sided Mann-Whitney-Wilcoxon test was used to compare the expansion of PSC-associated clonotypes.

To validate the identified clonotypes, we analyzed their expansion in two independent cohorts. First, the ‘A Study of a Prospective Adult Research Cohort with IBD’ (SPARC IBD) cohort of the US-based Crohnʼs and Colitis Foundation inflammatory bowel disease (IBD) Plexus program^25^ and, second, a PSC cohort from Norway (Methods). The SPARC IBD cohort contained 2,487 individuals with IBD only and 73 individuals with PSC-IBD. The identified 1,008 PSC-associated clonotypes were expanded significantly in people with PSC-IBD relative to IBD without PSC (*P* = 3.76 × 10^−5^) (Extended Data Fig. 1a). Additionally, we compared the expansion of PSC-associated clonotypes in 154 people with PSC and 64 healthy controls from Norway, showing a significant expansion of these clonotypes in PSC (*P* = 1.16 × 10^−9^) (Extended Data Fig. 1b).

**Extended Data Fig. 2:**
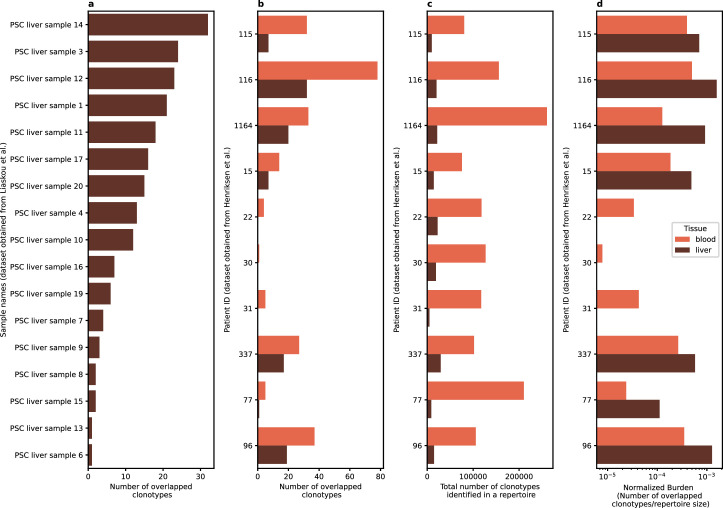
The identified 1,008 PSC-associated clonotypes are detected and expanded in PSC livers. (**a**) The number of PSC-associated clonotypes that were detected in different PSC-liver repertoires. The PSC liver TRB repertoires were obtained from Liaskou *et al.*^26^. (**b**) shows the number of PSC-associated clonotypes that were detected in the blood and liver repertoires of individuals with PSC. (**c**) depicts the total number of unique clonotypes identified in the blood and liver of each individual in the utilized dataset. (**d**) illustrates the normalized burden of PSC-associated clonotypes in the liver as well as blood of people with PSC. The dataset used in (**b**), (**c**), and (**d**) is derived from Henriksen *et al.*^27^.

To investigate whether PSC-associated clonotypes identified from blood were also present and/or expanded in affected PSC livers, we utilized a previously published dataset containing TRB repertoires of PSC livers^26^ (Extended Data Fig. 2a). There was an overlap between PSC-associated clonotypes and PSC liver repertoires with a median of 12 PSC-associated clonotypes per repertoire. To investigate their contribution to the blood compared with the liver repertoire, we utilized another previously published dataset^27^ with paired blood and liver repertoires of people with PSC. Whereas the number of overlapping clonotypes was higher in blood than in liver biopsies (Extended Data Fig. 2b), the number of identified clonotypes in these two tissues, that is, the repertoire size, was different (Extended Data Fig. 2c). After normalizing by repertoire size, we observed a higher abundance of PSC-associated clonotypes in PSC livers relative to PSC-blood (Extended Data Fig. 2d). These findings indicate that the identified 1,008 PSC-associated clonotypes were not only expanded in blood but also in the liver of people with PSC, suggesting that these clonotypes may be involved in the ongoing liver pathology.

### PSC-associated clonotypes are restricted to risk HLA alleles

**Extended Data Fig. 3:**
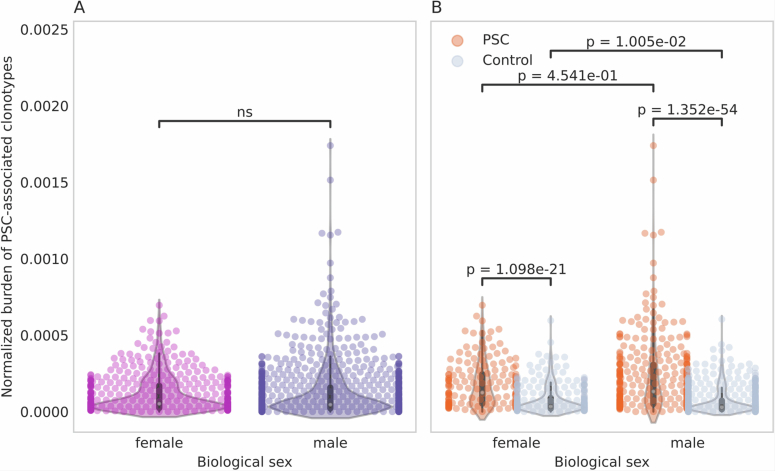
The normalized burden of PSC-associated clonotypes is not associated with biological sex. (**a**) The lack of difference in the normalized burden in females (n = 471 individuals) relative to males (n = 937 individuals) irrespective of the underlying disease across the entire cohort of PSC and healthy controls. (**b**) The burden of PSC-associated clonotypes was significantly higher in individuals with PSC relative to healthy controls independent of the biological sex. This analysis was performed across the entire cohort which contains 304 females without PSC, 167 females with PSC, 600 males without PSC and 337 males with PSC. In both panels, each dot represents the cumulative expansion of all PSC-associated clonotypes in the repertoire of an individual, black lines represent the median across different individuals, the boxplots represent the interquartile range (IQR) and the whiskers represent datapoints within the 1.5 times the IQR range. Lastly, a two-sided Mann-Whitney-Wilcoxon test was used to compare the normalized burden across the different groups included in the study.

**Extended Data Fig. 4:**
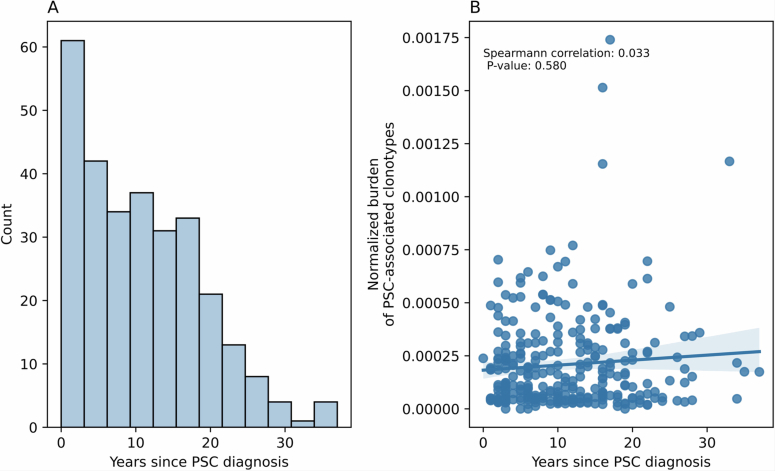
The relationship between the burden of PSC-associated clonotypes and years since PSC-diagnosis. (**a**) The distribution of years since diagnosis across the cohort. (**b**) The correlation between years since PSC diagnosis and the normalized burden of PSC-associated clonotypes. In both panels, the TRB repertoires of 289 individuals with PSC was included in the analysis.

Subsequently, we investigated the relationship between repertoire size and the number of PSC-associated clonotypes (*n* = 1,008), that is, the burden of PSC-associated clonotypes, which correlated poorly with repertoire size (Spearman correlation, ~0.19; Fig. 1b). Additionally, the number of PSC-associated clonotypes per repertoire was higher in people with PSC relative to controls (*P* = 2.9 × 10^−43^; Fig. 1c). To investigate factors shaping the burden of PSC-associated clonotypes, we normalized the burden of these clonotypes by repertoire size to produce a single number that represents the fraction of PSC-associated clonotypes in the repertoire. The normalized abundance did not correlate with the biological sex (Extended Data Fig. 3) or years since diagnosis (Extended Data Fig. 4). Given that conventional TCRs are restricted to particular HLA alleles, we tested whether differences in the HLA background of the study participants (Methods) might explain the difference in the normalized burden.

**Extended Data Fig. 5:**
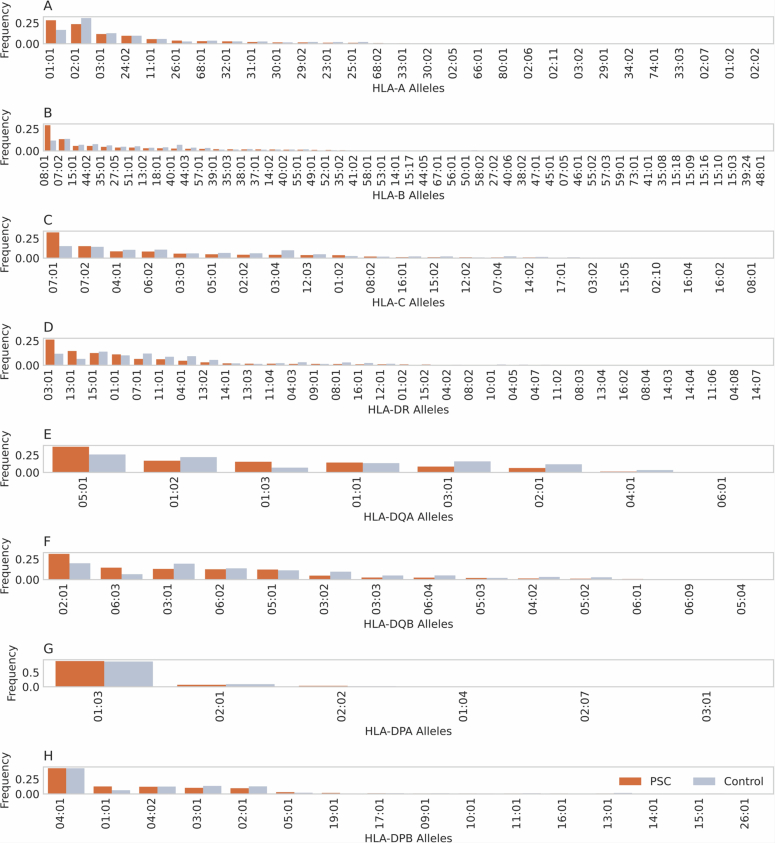
The frequency of different HLA alleles in the discovery cohort of individuals with PSC and healthy controls. (**a**) The frequency of HLA-A alleles in the PSC cohort and the healthy cohort. (**b**), (**c**), (**d**), (**e**), (**f**), (**g**) and (**h**), The frequency of HLA-B alleles, HLA-C alleles, HLA-DRB1 alleles, HLA-DQA1 alleles, HLA-DQB1 alleles, HLA-DPA1 alleles and HLA-DPB1 alleles in the PSC cohort and the healthy cohort, respectively. In all panels, the imputed HLA alleles from genotyping arrays for 431 individuals with PSC and 773 individuals without PSC were analyzed.

The alleles of the ancestral HLA haplotype 8.1 (AH 8.1) (HLA-(A*01:01, C*07:01, B*08:01, DRB1*03:01, DQA1*05:01 and DQB1*02:01)) were enriched in peoples with PSC compared with healthy controls (for example, for HLA-B*08:01, the frequency was 29% in PSC compared with 11% in controls; Extended Data Fig. 5). Given this strong association, we aimed to compare the normalized burden between carriers and noncarriers of these alleles, focusing on the two alleles with the strongest PSC association: HLA-B*08:01 (Fig. 1d) and HLA-DRB1*03:01 (Fig. 1e). The burden was dependent on two factors, the HLA background and disease status, with PSC carriers having the highest burden and noncarrier controls having the lowest. This finding suggests that PSC-associated meta-clonotypes represent public, HLA-restricted clonotypes that are present in carriers of AH 8.1 alleles but are significantly expanded in people with PSC relative to controls.

**Fig. 2:**
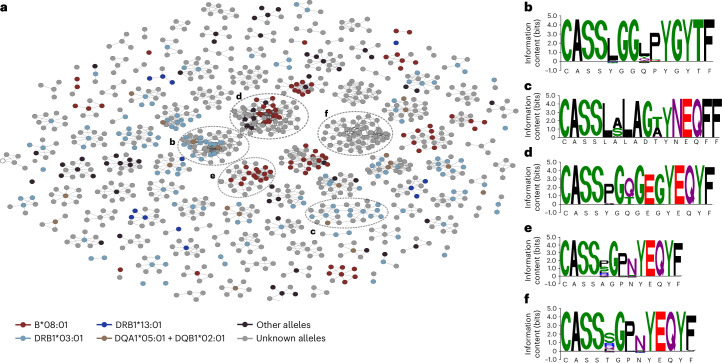
PSC-associated clonotypes are restricted to PSC risk HLA alleles. **a**, Arrangement of primary sclerosing cholangitis (PSC)-associated clonotypes into several clusters. To build these clusters, we established an edge between two clonotypes (nodes in the graphic above) if the two clonotypes share the same V and J genes and differ in their complementarity-determining region 3 (CDR3) sequence by one amino acid only. **b**,**c**, The CDR3 motifs of the two main clusters associated with the HLA-DRB1*03:01 allele. Each of these clusters is derived from a different V/J gene combination and exhibits different CDR3 sequence motif depicted in (**b**) and (**c**). **d**,**e**, Two main CDR3 motifs associated with the HLA-B*08:01 allele each with a distinct V/J gene usage and a CDR3 sequence. **f**, Motif derived from the largest cluster without a known HLA restriction. In **b**–**f**, amino acids are colored according to their physiochemical properties, whereas the height of each amino acid representation can be interpreted as the degree of conservation of a specific amino acid at a specific position within a CDR3 motif.

To discover the cognate HLA alleles of PSC-associated clonotypes, we queried the HLA-DB database^28^. Out of the 1,008 PSC-associated clonotypes, 332 were observed in the HLA-DB. Of these, 87 were specific to HLA-B*08:01, 121 to HLA-DRB1*03:01, 21 to the HLA-DQA1*05:01-DQB1*02:01 heterodimer and 16 could not be distinguished between HLA-DRB1*03:01 and the HLA-DQA1*05:01-DQB1*02:01 heterodimer due to the strong linkage disequilibrium of the AH 8.1 haplotype (Fig. 2a). A smaller subset of clonotypes was also specific to HLA-DRB1*13:01—another known PSC risk allele (Fig. 2a). There were also several clusters without any known HLA association (Fig. 2a), these clonotypes might be restricted to HLA alleles that are rarer in the United States where the HLA-DB^28^ was developed. Alternatively, they might be recognizing antigens that are not encountered at high frequency in healthy people and hence absent from the HLA-DB.

Next, we investigated common motifs within PSC-associated clonotype clusters (Fig. 2a). We started by analyzing the two main clusters that harbored multiple HLA-DRB1*03:01-restricted clonotypes. These clusters were defined by a clear motif that varied only in three (Fig. 2b) or two (Fig. 2c) amino acid sites. A similar pattern was observed for the two main clusters containing several HLA-B*08:01-restricted clonotypes (Fig. 2d,e). The complementarity-determining region 3 (CDR3) amino acid motif for the biggest cluster without a known HLA allele (Fig. 2f) showed high sequence similarity to one of the HLA-B*08:01-restricted clusters (Fig. 2e). By comparing the V and J gene usages among the two clusters, we found that clonotypes derived from the unknown cluster (Fig. 2f) belong to the TCRBV*07 gene family whereas the HLA-B*08:01-restricted cluster (Fig. 2e) contains TCRBV*07:02-derived clonotypes. This suggested that clonotypes derived from these two clusters (Fig. 2d,f) were probably a single cluster restricted to HLA-B*08:01 and are potentially responding to the same antigen. Thus, even though we identified over 1,008 PSC-associated clonotypes, several of these clonotypes were restricted to known PSC risk alleles and shared a considerable degree of sequence similarity, suggesting that they recognize a limited set of antigens.

### PSC-associated clonotypes recognize mainly EBV antigens

**Fig. 3:**
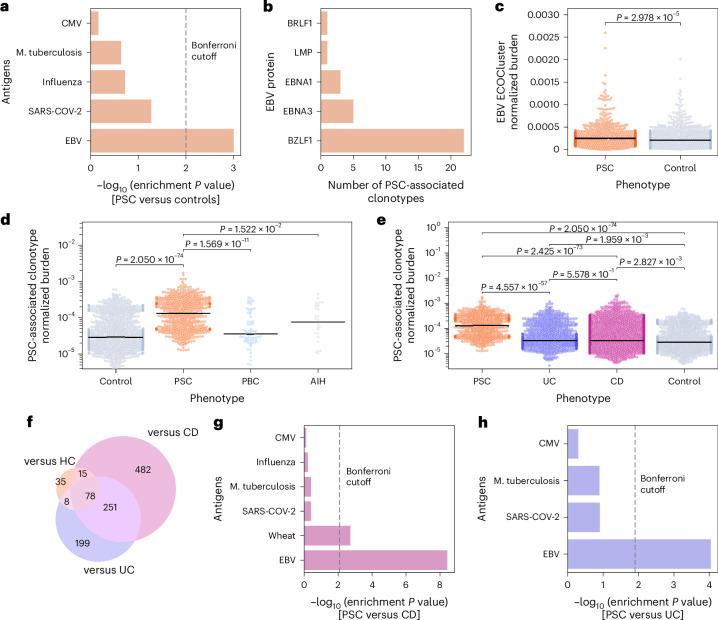
Pathogen enrichment analysis of PSC-associated clonotypes. **a**, Enrichment of primary sclerosing cholangitis (PSC)-associated clonotypes identified by comparing the TCR beta (TRB) repertoires of people with PSC relative to controls. This enrichment analysis is based on the number of overlapping clonotypes, for example Epstein-Barr virus (EBV; *n* = 6 clonotypes), cytomegalovirus (CMV; *n* = 3 clonotypes) and *M. tuberculosis* (Mtb; *n* = 1 clonotypes) and the number of pathogens-specific clonotypes in the public datasets. **b**, The strong response against the lytic phase master regulator BZLF1 protein of EBV obtained by searching the list of 1,008 PSC-associated clonotypes in the MIRA-generated annotation database (MIRA database); *y* axis, different EBV proteins; *x* axis, number of PSC-associated clonotypes that recognize peptides derived from these proteins. **c**, Burden of the EBV ECOCluster in people with PSC (*n* = 504) and healthy controls (*n* = 904). This burden was calculated by dividing the number of clonotypes overlapping with the EBV ECOCluster by the total number of clonotypes identified in a sample’s repertoire. **d**, Difference in the normalized burden of PSC-associated clonotypes (*n* = 1,008) in PSC relative to controls as well as two autoimmune liver diseases, namely primary biliary cholangitis (PBC; *n* = 63) and autoimmune hepatitis (AIH; *n* = 28). **e**, Burden of PSC-associated clonotypes in PSC relative to controls, people with Crohn’s disease (CD; *n* = 2,037) and people with ulcerative colitis (UC;*n* = 844). **f**, Overlap in the sets of PSC-associated clonotypes identified by comparing the TRB repertoire of people with PSC with healthy controls (versus HC), people with UC (versus UC) and people with CD (versus CD). These sets of clonotypes were derived by comparing the different phenotypes, that is, CD, UC and controls with PSC. **g**,**h**, Pathogen enrichment analysis of PSC-associated clonotypes identified by comparing the TRB repertoire of people with PSC relative to people with CD (**g**) or UC (**h**). In **c–e**, each dot represents an individual, that is, the repertoire of a study participant, and black lines represent the median. All statistical comparisons were conducted using the two-sided Mann–Whitney–Wilcoxon test without correction for multiple testing.

Subsequently, we sought to identify the antigen-specificity of PSC-associated clonotypes. First, we investigated the overlap between these clonotypes and public datasets, namely VDJdb^29^ and McPAS-TCR^30^. Given that most PSC-associated meta-clonotypes have been observed only rarely in public datasets, we focused on the main 136 clonotypes identified before seeded clustering (Supplementary Table 1). Clonotypes were annotated with their antigenic target if they had a match in the database with the exact V and J gene as well as CDR3 amino acid sequence. The overlapping TRB clonotypes (*n* = 12) were specific towards common pathogens such as EBV (*n* = 6), CMV (*n* = 3) and *Mycobacterium tuberculosis* (n = 1). Given that the entries per pathogen in public databases vary, we calculated an enrichment *P* value using a hypergeometric test as employed in traditional gene enrichment analyses (Methods). As seen in Fig. 3a, only EBV-specific TRB clonotypes were enriched in people with PSC relative to controls (*P* = 9.96 × 10^−4^).

**Extended Data Fig. 6:**
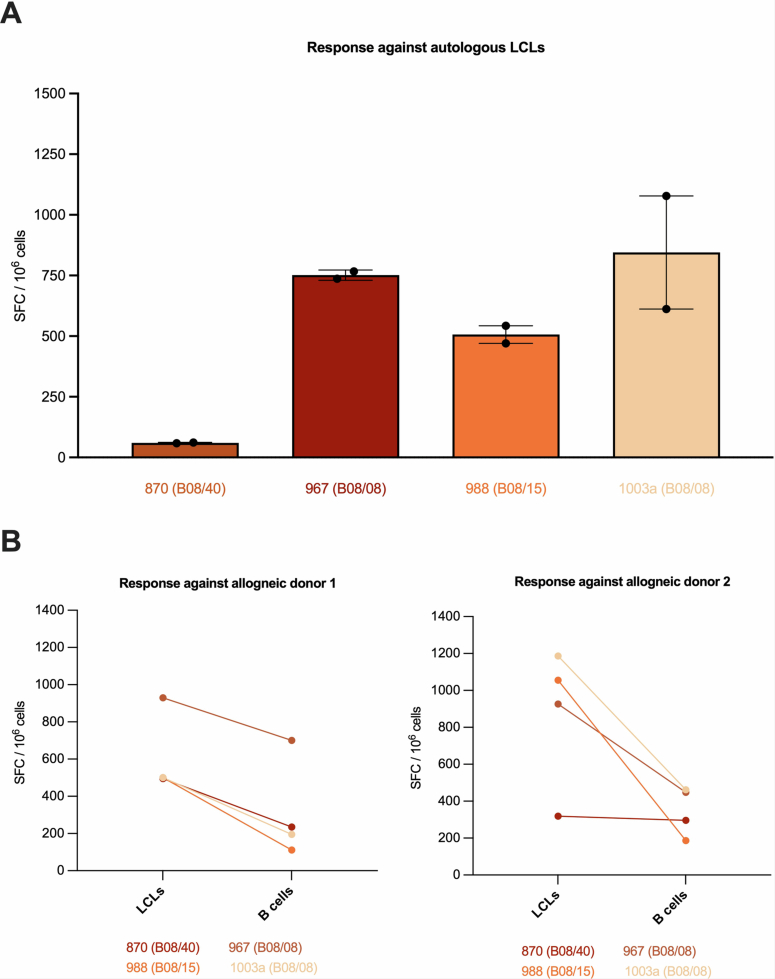
EBV-specific response of expanded T cell lines against EBV-LCLs and non-transformed B cells in IFN-gamma ELISpot. Response of T cell lines from individuals with PSC (n = 4) towards autologous Epstein–Barr virus lymphoblastoid cell lines (EBV-LCLs) in an effector:target (E:T) ratio of 1:1 (**a**) or against EBV-LCLs and non-transformed B cells in E:T ratio of 5:1 from two partially HLA-matched allogenic donors (**b**). Plotted values represent the mean and standard deviation of the spot-forming colonies (SFC) per 10^6^ cells subtracted by the spot-forming colonies (SFC) per 10^6^ cells of the unstimulated T cell controls (negative controls ranging from 0–7 SFC/10^6^ cells) of two technical replicates.

We also queried a peptide–TCR interaction dataset that was generated using the MIRA assay^31^ and hosted by Adaptive Biotechnologies, which also showed an enrichment of EBV-specific clonotypes (Supplementary Table 3). Most of the clonotypes overlapping with the MIRA assay were known to recognize epitopes derived from the EBV lytic phase protein BZLF1 (*n* = 22 clonotypes out of 7,403 in the database) followed by the EBV latency III protein EBNA3 (*n* = 5 clonotypes out of 1,109 clonotypes) (Fig. 3b). To validate this experimentally, we enriched for EBV-specific TRB clonotypes using either HLA-B*08:01 tetramers loaded with peptides from the BZLF1 and EBNA3 proteins or using antigen stimulation with peptide pools derived from the BZLF1 and EBNA1 proteins followed by sorting of activated cells (Methods). Out of the 1,008 PSC-associated clonotypes we detected 13 clonotypes that overlapped with the sorted EBV-specific TRB clonotypes (*n* = 944), corroborating the in silico analyses. To further validate T cell recognition of EBV-infected B cells in an HLA-restricted manner in people with PSC, we assessed EBV-specific T cell reactivity using autologous and allogeneic HLA-B*08-matched EBV-transformed lymphoblastoid cell lines (LCLs) as well as untransformed B cells (Methods). Our findings revealed that PSC-derived T cell lines effectively recognized autologous LCLs (Extended Data Fig. 6). Additionally, we observed enhanced reactivity against LCLs compared with untransformed B cells when using allogeneic donor targets (Extended Data Fig. 6). To further characterize the disease-specific functionality of EBV-specific T cells in people with PCS, future studies will need to include healthy control samples and other disease controls.

We recently used co-occurrence clustering of common, HLA-associated clonotypes in 30,674 donor-repertoires to identify clonotype groupings that represent the public immune signature to common exposures (exposure co-occurrence clusters, hereafter referred to as ECOClusters), including EBV^32^. Utilizing co-occurrence clusters with our dataset, we identified 35 PSC-associated clonotypes in the previously determined 9,704 clonotypes EBV-specific ECOCluster^32^. The HLA-matched clonotype burden of the EBV ECOCluster was significantly higher in PSC relative to healthy controls (*P* = 2.97 × 10^−5^; Fig. 3c). Thus, the co-occurrence analysis further supported the association of clonotypes specific to EBV in PSC and the link to known PSC HLA susceptibility alleles.

### PSC-associated clonotypes are specifically expanded in PSC

To ensure that PSC-associated clonotypes were specific to PSC and not a general marker of liver injury, we calculated the normalized burden of PSC-associated clonotypes in other autoimmune liver diseases, that is, primary biliary cholangitis (PBC; *n* = 63 repertoires) and autoimmune hepatitis (AIH; *n* = 28 repertoires). The normalized burden was significantly higher in PSC relative to PBC samples (*P* = 1.6 × 10^−11^) and AIH samples (*P* = 1.2 × 10^−2^) (Fig. 3d). To exclude the possibility that PSC-associated clonotypes were confounded by concurrent IBD, which occurs in 60–80% of people with PSC^1^, we determined the burden of these clonotypes in 2,037 people with Crohn’s disease (CD) and 844 people with ulcerative colitis (UC), based on a recently described dataset^33^. Again, the normalized burden of PSC-associated clonotypes was higher in PSC relative to UC (*P* = 4.5 × 10^−57^) and CD (*P* = 2.4 × 10^−73^) (Fig. 3e). Additionally, we compared the TRB repertoires of people with PSC with that of people with CD (*n* = 2,037) and UC (*n* = 844) using the approach used to identify PSC-associated clonotypes. Across the three comparisons, the same set of PSC-associated clonotypes were identified repeatedly (Fig. 3f). Subsequently, we ran a pathogen enrichment analysis on PSC-associated clonotypes identified from comparing PSC samples with either UC or CD, which showed that EBV-specific clonotypes were specifically enriched in PSC (versus CD, *P* = 3.71 × 10^−9^ (Fig. 3g); versus UC, *P* = 9.1 × 10^−5^ (Fig. 3h)). These findings suggest that PSC-associated clonotypes were indeed specific to PSC and not shared with other chronic immune-mediated diseases of the liver and the bowel, such as PBC, AIH and IBD.

To investigate whether the expansion of PSC-associated clonotypes was different between people with PSC with and without IBD, that is, PSC and PSC-IBD, we compared the normalized burden of PSC-associated clonotypes among three groups, PSC (*n* = 127 people), PSC-CD (*n* = 33 people) and PSC-UC (*n* = 167 people), showing no significant difference among these three groups. We next used the same statistical framework utilized for discovering PSC-associated clonotypes to discover clonotypes associated with each of these subphenotypes. Whereas there was a varying level of overlap among the associated clonotypes of the different subgroups, anti-EBV responses were a common theme among these different clonotype sets. Specifically, five, 12 and four of the meta-clonotypes associated with PSC, PSC-UC and PSC-CD were EBV-specific, respectively. These findings indicate that elevated anti-EBV T cell responses are a common feature in PSC and its relevant subphenotypes.

### Increased antibody responses against EBV in PSC

PhIP–seq enables the screening of antibody specificities by testing a predesigned subset, often several 100,000s, of peptides (antigens) against serum antibodies^24^ of an individual. PhIP–seq has been used to understand factors shaping the immune response to different antigens^34,35^, as well as for identifying the exposure signature of different diseases such as chronic fatigue syndrome^36^ and inflammatory bowel disease^37^.

**Fig. 4:**
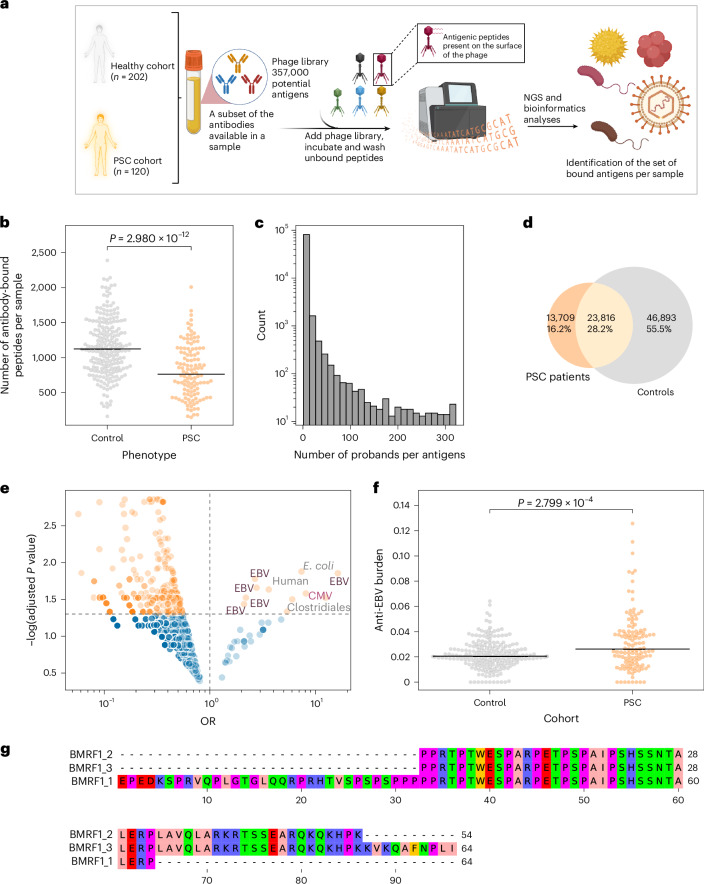
The antibody repertoire of people with PSC is marked by a higher burden of anti-EBV responses. **a**, Overview of the framework used to decode the exposure history of people with primary sclerosing cholangitis (PSC) and healthy controls using phage-immunoprecipitation sequencing (PhIP–seq). Briefly, sera were incubated with a bacteriophage library containing 357,000 peptides expressed on the capsids of the library bacteriophages. Subsequently, immunoprecipitation was conducted to pulldown antibody–bacteriophage complexes. After washing away unbound bacteriophages, DNA was extracted, and the inserted peptide sequences were amplified using PCR and then sequenced to identify target antigens that were recognized by the antibodies in the sera. **b**, Reduction in the number of bound antigens by the PSC cohort (*n* = 115) relative to controls (*n* = 201). **c**, Number of individuals binding to a particular antigen across all antigens included in the library. **d**, Number of antigens identified only in controls, in people with PSC or in both cohorts. **e**, Incidence analysis of antigens bound by the PSC and healthy cohorts; before the analysis, we calculated the prevalence of each bound antigen in the PSC and healthy cohorts, and we restricted our analysis to antigens with an absolute difference in prevalence between the two cohorts of more than 0.05 (that is, 5% differences in prevalence between the two cohorts). **f**, Burden of anti-Epstein-Barr virus (EBV) responses in the PSC cohort (*n* = 115) relative to the healthy control cohort (*n* = 201). The immune response burden was calculated by dividing the number of bound EBV antigens by the total number of antigens identified in the functional antibody repertoire. **g**, The antigenic region of BMRF1, which is covered by three independent peptides identified from the incidence analysis shown in **e**. In **g**, the color scheme reflects the physicochemical properties of the amino acids. In **b** and **f**, each dot represents an individual, that is, the repertoire of a study participant, and black lines represent the median. Additionally, the two-sided Mann–Whitney–Wilcoxon test was used to compare the functional antibody repertoire of people with PSC and healthy controls. Panel **a** was created using BioRender.com.

To complement the results of the TCR profiling, and to analyze B cell responses, we performed PhIP–seq on sera from 202 healthy controls and 120 people with PSC, screening 357,000 peptides in parallel per serum sample (Fig. 4a). Relative to controls, PSC samples had a significantly lower number of bound antigens, that is, their serum antibodies recognized fewer antigens from the library relative to controls (Fig. 4b). Similar to previous PhIP–seq studies in other cohorts^34,35,37^, most antigens in the library were detected in a single donor, that is, most responses were private (Fig. 4c).

Given the larger control sample size, most antigens were observed only in healthy controls (55%), with 16% only in people with PSC and 28% in both PSC and matching controls (Fig. 4d). When we compared the prevalence, that is, the frequencies of recognized antigens in individuals with PSC versus healthy controls, using the Fisher’s exact test, we observed a significantly higher prevalence in anti-EBV responses in people with PSC (Fig. 4e). We detected several overlapping antigens derived from the EBV protein BMRF1, also known as the early antigen diffuse component, which is a lytic phase protein (Fig. 4g). Globally, the fraction of anti-EBV responses relative to all bound antigens was significantly higher in people with PSC relative to controls (Fig. 4f). Thus, the PhIP–seq results complemented and corroborated the TCR analyses and showed that an expanded response towards EBV in PSC is evidenced for both T cells and B cells.

### In PSC there is a robust EBV reactivation

**Fig. 5:**
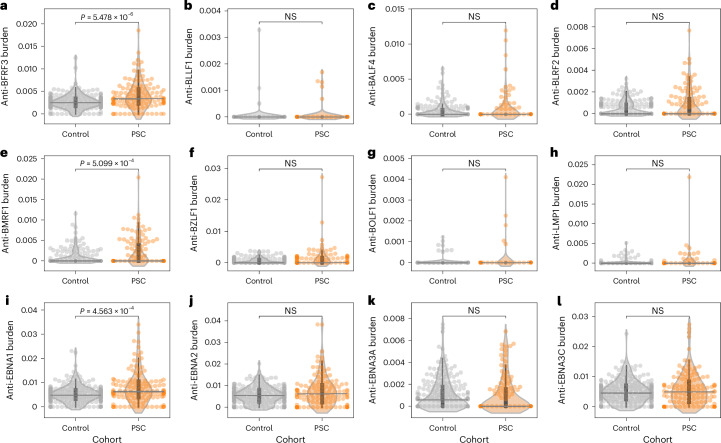
Expansion of immune responses toward different EBV antigens in PSC and controls as inferred from PhIP–seq data. In all panels, the burden of a particular protein in a sample refers to the number of antigens derived from this protein divided by the total number of antigens recognized in this sample, which was calculated for people with (*n* = 115) or without (*n* = 201) primary sclerosing cholangitis (PSC). **a**–**d**, Burden of capsid and structural Epstein-Barr virus (EBV) antigens, namely, BFRF3 (**a**), BLLF1 (**b**), BALF4 (**c**) and BLRF2 (**d**). **e**–**h**, Burden of lytic phase proteins, namely, BMRF1 (**e**), BZLF1 (**f**), BOLF1 (**g**) and LMP1 (**h**). **i**–**l** Burden of different latency program antigens, namely EBNA1 (**i**), EBNA2 (**j**), EBNA3A (**k**) and EBNA3C (**l**). In all panels, each dot represents the burden of response against a particular EBV protein in an individual and black lines represent the median across different people, the boxplots represent the IQR and the whiskers represent datapoints within the 1.5 times the IQR range. All statistical comparisons were conducted using the two-sided Mann–Whitney–Wilcoxon test.

Given the increased anti-EBV responses observed in people with PSC relative to controls, we investigated whether this is uniformly targeting all EBV antigens or is directed toward a subset of these antigens. Therefore, we compared antibody responses against different EBV antigens in people with PSC and controls, focusing on three groups of antigens defined in the utilized PhIP–seq library: (1) structural and capsid proteins, (2) lytic phase proteins and (3) latency phase proteins. Within the structural and capsid proteins group, only BFRF3 had a significantly higher burden in people with PSC relative to controls (Fig. 5a–d), and antibodies against BFRF3 were also shown to be cross-reactive to human proteins such as septin-9 (ref. ^38^). Within the lytic phase antigens, only BMRF1 had a significantly higher burden in PSC relative to controls (Fig. 5e–h). Within the subset of latency proteins (Fig. 5i–l), only EBNA1 had a higher response burden in people with PSC relative to controls (Fig. 5i).

We then used the PhIP–seq data to impute EBV seropositivity rates in people with PSC and controls using three EBV proteins: BMRF1 (early antigen diffuse component), EBNA1 and BFRF3 (part of the viral capsid antigen), defining positivity as having three bound antigens or more per protein. This resulted in an EBV seropositivity rate of 27.8% in people with PSC and 14.9% in controls based on BMRF1 (*P* = 0.008), 80.8% in PSC and 85% in controls based on EBNA1 (*P* = 0.34) and 74.7% in PSC and 67.6% in controls based on BFRF3 (*P* = 0.20). We also defined EBV-positivity as having a positive response toward any of these three proteins, resulting in a positivity rate of 90.4% in people with PSC and 91% in controls (*P* = 0.84). These data indicate that, in PSC, there was an elevated immune response toward lytic phase antigens, suggesting EBV reactivation in PSC similar to other autoimmune diseases such as rheumatoid arthritis (RA)^39^, multiple sclerosis (MS)^40^ and Sjögren syndrome^41^. Nonetheless, there is a small fraction of EBV-negative people with PSC, possibly due to phenotype heterogeneity (PSC-like conditions caused by other etiologies) or a reflection that PSC in rare cases may arise also in an EBV-negative setting.

### Expanded B cells in PSC livers produce anti-EBV antibodies

**Fig. 6:**
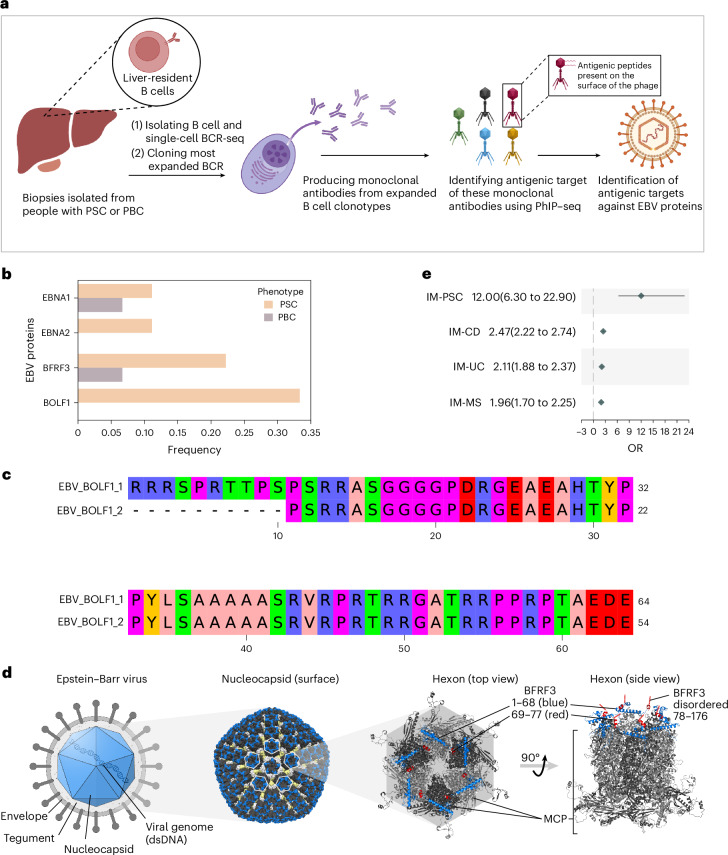
Expanded liver-resident B cells in people with PSC show a higher anti-EBV response burden relative to PBC. **a**, Steps used to generate monoclonal antibodies from liver-derived B cells and to decode their antigenic specificities. Briefly, single-cell B cell receptor sequencing was conducted on B cells isolated from liver biopsies. Subsequently, expanded B cell clonotypes were cloned into a mammalian expression system to produce monoclonal antibodies. The antigenic specificities of these monoclonal antibodies was identified using phage-immunoprecipitation sequencing (PhIP–seq). **b**, Frequency of anti-EBV monoclonal antibodies generated from expanded B cells in the liver of three PSC (*n* = 9 total antibodies) and three PBC liver biopsies (*n* = 15 total antibodies). Frequency was calculated across the produced monoclonal antibodies and not the number of samples. **c**, The antigenic region of BOLF1 to which monoclonal antibodies from the liver of people with PSC bind. **d**, Three-dimensional location of BFRF3 (blue) on the surface of the EBV capsid structure. The capsid is composed of 11 pentons and 150 surrounding hexons^56^, which are complexes of several different proteins, among which a multimer of the main capsid protein (MCP, light and dark gray cartoons) provides for the main structural basis together with BFRF3 cross-linking on the top. BFRF3 also mediates Tegument binding through the flexible C terminus^56^. The antigenic peptide ranges from 69 to 122 and is partly structured (red helices, positions 69–77) but mostly disordered (indicated schematically as a thin red line). **e**, Association of different autoimmune and immune-mediated inflammatory diseases, including primary sclerosing cholangitis (PSC), Crohn's disease (CD), ulcerative colitis (UC) and multiple sclerosis (MS) with infectious mononucleosis (IM), with filled diamonds representing the association odds ratio (OR) and error bars representing the 95% CI. Panel **a** was created using BioRender.com.

We further investigated liver-infiltrating B cells in PSC by collecting liver biopsies from three people with PSC and three people with PBC (as a control group). Subsequently, we produced monoclonal antibodies from expanded B cells (Methods) and characterized their cognate antigens using PhIP–seq (Fig. 6a). Given the small sample size and building from our previous findings, we focused our analysis on EBV antigens, which were found to have a higher frequency in PSC relative to PBC livers (Fig. 6b). Due to the small sample size, we were not able to perform robust statistical testing to compare the frequency of EBV bound antigens between PSC and PBC samples. Nonetheless, antibodies that had higher abundance in the sera of people with PSC relative to controls were also detected at a higher frequency in PSC livers. These included antibodies against the small capsid protein (SCP/BFRF3) (Fig. 5a) as well as EBNA1 (Fig. 5i). We also observed antibody responses against two additional epitopes from the EBV lytic phase protein BOLF1 (Fig. 6c), corroborating our previous findings about the higher burden of anti-EBV responses in PSC relative to controls. Mapping the BFRF3 antigenic region bound by the monoclonal antibody present in PSC livers onto the three-dimensional structure of the protein pointed towards the partially disordered C-terminal region of the capsid protein as a key epitope (Fig. 6d).

### Infectious mononucleosis is associated with PSC

To assess the epidemiological plausibility of our findings, we investigated a potential link between infectious mononucleosis, which is commonly caused by an EBV infection^42^, and PSC. To this end, we used the TriNetX database (Methods), containing >116 million electronic health records, to associate infectious mononucleosis with PSC (Fig. 6e). Infectious mononucleosis was associated significantly with PSC (odds ratio (OR) = 12; 95% confidence interval (CI), 6.3–22.9), which was higher than for inflammatory bowel disease, specifically, CD (OR = 2.47; 95% CI, 2.22–2.74) and UC (OR = 2.11; 95% CI, 1.88–2.37), as well as for MS (OR = 1.96; 95% CI, 1.7–2.25). Overall, these findings support an immunological link between EBV and PSC.

## Discussion

Using large-scale high-throughput immune-profiling technologies such as TCR-seq and PhIP–seq in addition to epidemiological data, we were able to establish a strong association between EBV and PSC. EBV has been implicated previously in multiple autoimmune diseases and immune-mediated inflammatory diseases^43^ such as MS^44^, RA^39^, systemic lupus erythematosus (SLE)^45^ and Sjögren syndrome^46^. EBV reactivation—a process where the virus exits its latency and starts its lytic program—has been observed in several autoimmune diseases, for example, SLE^47^, RA^39^ and in MS^40^. We observed higher antibody responses against the BMRF1 protein and elevated T cell-mediated responses against the BZLF1 protein, both of which are lytic phase proteins, in people with PSC relative to healthy controls, suggesting that EBV reactivation is probably involved in the pathogenesis of PSC, similar to other autoimmune diseases^43^.

Although the contribution of EBV and EBV reactivation in autoimmune diseases remains poorly understood, different mechanisms, such as molecular mimicry, have been proposed. Within SLE cross-reactivities between the EBNA1 protein and Ro60 (ref. ^48^) as well as C1q^49^ were observed, similarly, in MS between EBNA1 and GlicalCAM^50^. Here, we observed a significant increase in the anti-EBNA1 responses in people with PSC relative to controls. Whereas this may suggest the presence of a yet-to-be-discovered cross-reactive self-antigen in PSC, it could also indicate a dysregulated immune response toward EBV antigens. Specifically, viral proteins produced during viral replication can have immunomodulatory effects, for example, BZLF1 can inhibit INFγ signaling^51^ and the production of inflammatory cytokines^52^. Additionally, BMRF1 inhibits responses to DNA damage^53^ and BZLF1 interferes with the cell cycle to increase EBV viral production^52^.

Although the molecular data provide evidence for the involvement of EBV in the pathophysiology of PSC, it does not establish a causal relationship between EBV and PSC due to the cross-sectional study design. Using temporal data from, for example, the Department of Defense Serum Repository^54^, a relationship between EBV infection before the onset of MS could be established^44^. Similar observations have also been recently made for IBD^55^. A common feature of these studies is the long timespan between EBV infection and the onset of these diseases. This suggests that multiple rounds of EBV reactivation, additional risk factors/triggers and/or a prolonged interaction between EBV and the host immune system are needed for disease development.

The TCR-seq analysis enabled us to identify TRB clonotypes that are expanded in people with PSC; however, it did not provide the functional state of T cells expressing these TRB chains, for example, their subtypes and gene expression patterns. Furthermore, most of the identified clonotypes were restricted to known HLA risk alleles, such as HLA-B*08:01 and HLA-DRB1*03:01, which are frequent in people with PSC but are not PSC specific. Whereas our PhIP–seq library covered multiple EBV epitopes, it did not cover the entire antigenic space of EBV; subsequently, only a subset of potential epitopes could be measured in the current study. Follow-up studies should focus on investigating the entire antigenic space of EBV using tailored libraries, and replication and elaboration of relevant antigenic specificities in diverse PSC cohorts from other geographical regions are warranted. Additionally, quantification of the EBV seropositivity rate in large PSC cohorts would be needed to further disentangle the role of EBV in the pathogenesis of PSC and recurrent PSC after liver transplantation.

In conclusion, our analysis has established a strong link between EBV infection and PSC. Further studies are needed to elucidate potential mechanisms by which infectious mononucleosis and EBV infection contribute to PSC development.

## Methods

### Ethical approval

The study was approved by the ethical committee at the University of Kiel under the following ethical votes: D441/16, D474/12, A161/08, A103/14 and A148/14, and by the Regional Committees for Medical and Health Research Ethics of South-Eastern Norway (reference numbers 18221 and 13381). Written informed consent was collected from all participants before the beginning of the study. For isolating monoclonal antibodies from liver samples, fresh PSC and PBC liver explants were collected at Queen Elizabeth Hospital in Birmingham, UK and at Oslo University Hospital Rikshospitalet in Oslo, Norway. As per the Declaration of Helsinki, written informed consent was obtained from participants in accordance with local research ethics committee approvals in Birmingham (LREC no. 06-Q2702-61) and Regional Committees for Medical and Health Research Ethics of South-Eastern Norway (reference numbers 13381, 18221 and 15368).

### Study cohorts

PSC samples were collected from three tertiary referral centers across Germany, namely the University Medical Center Hamburg–Eppendorf, Hannover Medical School and Charité—Berlin University Medicine. Detailed clinical information related to these samples is shown in Extended Data Table 1. The samples used for TCR-seq (*n* = 504) were collected from 2008 to 2020, whereas the serum samples utilized for PhIP–seq measurements (*n* = 120) were collected from 2015 to 2021 primarily at the University Medical Center Hamburg–Eppendorf. Recruitment of participants and collection of biomaterials was coordinated through the biobank popgen (https://portal.popgen.de/). Specifically, popgen sent blank study sets to most of the recruiting clinics. The clinic included study participants and sent the biomaterials and questionnaires back to popgen. The study set contained blood sample kits, a questionnaire and an informed consent form. In some other cases, participants were asked by the clinic to take part in the study and subsequently contacted popgen to receive the study set. Healthy controls were derived from age- and sex-matched healthy blood donors (Extended Data Table 1) collected at the Institute of Transfusion Medicine through support of the Institute of Clinical Molecular Biology at the University Medical Center Schleswig-Holstein (UKSH). Samples included in the TCR-seq analysis (*n* = 904) were obtained from 2015 to 2017, whereas samples included in the PhIP–seq analysis (*n* = 202) were collected from 2016 to 2017.

### TCR profiling

Genomic DNA was extracted from frozen, plasma-depleted blood samples using the Qiagen DNeasy Blood Extraction Kit (Qiagen). As much as 18 μg of input DNA was then used to perform immunosequencing of the CDR3 regions of TCR-β (TRB) chains, that is the TRB repertoire, using the immunoSEQ assay (Adaptive Biotechnologies). Briefly, input DNA was amplified in a bias-controlled multiplex PCR, followed by high-throughput sequencing^16^. Sequences were collapsed and filtered to identify and quantify the absolute abundance of each unique TRB CDR3 region for further analyses^57^. To estimate the proportion of T cells out of total nucleated cells input for sequencing, or T cell fraction, a panel of reference genes present in all nucleated cells was amplified simultaneously^58^.

### Clonotype processing and filtration

We started by removing nonproductive clonotypes. Subsequently, we combined DNA rearrangement that yielded the same protein rearrangement together by summing their count and estimated frequency. Given the redundancy in the codon, different CDR3 nucleotide sequences will encode the same CDR3 amino acid sequence. Thus, we combined rearrangement with the same CDR3 protein sequence and different nucleotide sequences together as the protein sequence is the only relevant part for recognizing antigens presented by HLA proteins.

### Identifying PSC-associated clonotypes

PSC-associated clonotypes were identified using an incidence-based analytical framework^17^. First, we extracted public clonotypes, that is, clonotypes present in two or more people. For each public clonotype, we compared its frequency, that is its prevalence or occurrence, in the PSC and the healthy cohort using a one-sided Fisher’s exact test. Consequently, we utilized a permutation-based method^17^ to estimate the false discovery rate and the significance threshold for the association test. We considered any clonotype with a Fisher´s exact *P* value < 9 × 10^−5^ (<5% false discovery rate) to be PSC-associated. When comparing the repertoire of individuals with PSC with the repertoire of individuals with CD or UC, we used the same framework except that we used a two-sided Fisher’s exact test, then permutations were used to generate an analysis-tailored *P* value cutoff for a false discovery rate <5%.

### Seeded clustering of disease-associated clonotypes

To increase the number of disease-associated clonotypes, we developed a two-step clustering algorithm:

1. Clustering based on the sequence similarity.
  Here we used the amino acid sequence of the CDR3 to calculate the Levenshtein distance between each unique pair of PSC-associated clonotypes and all TRB clonotypes identified in our study cohort. Next, we selected pairs that have a Levenshtein distance of one, that is, clonotypes with higher sequence divergence were dropped from subsequent processing steps. The result of this step was a map between each PSC-associated clonotype and a collection of highly similar clonotypes that differ in their CDR3 sequence by one Levenshtein distance.
2. Clusters purification.
  To avoid introducing noise into the identified meta-clonotypes that were generated in step (1) based on sequence similarity only, we filtered the list of clonotypes present in a meta-clonotype by calculating the association strength in terms of *P* value between the seed and each member of the meta-clonotype. If the PSC-association *P* value of the seed and the candidate clonotype in the meta-cluster of the seed, was lower than the PSC-association *P* value of the seed only, then we assume that this candidate clonotype is also associated with PSC but because it has a lower frequency in the population, in other words lower prevalence, it was not associated with PSC in the main analysis using the Fisher's exact test. Nonetheless, if the calculated PSC-association *P* value was higher than the seed, we assume that this candidate clonotype is just a noise that is not associated with PSC. For each candidate clonotype in each meta-cluster identified in step (1), this filtration criterion is applied to removes noise introduced by sequence similarity and not by similarity in response, enabling us to identify a collection of highly similar clonotype sequences that are implicated in PSC.

### Global screening array genotyping and HLA imputation

We genotyped 431 people with PSC and 773 controls using the single nucleotide polymorphism global screening array from Illumina. Subsequently, classical HLA-I and HLA-II alleles were imputed at the two-field resolution, that is four-digits resolution, from these genotypes^59,60^.

### Generation of the TCR-seq validation datasets

Two datasets were used to validate the identified PSC-associated clonotypes. The first was derived from the SPARC IBD cohort of the Crohn’s and Colitis Foundation IBD Plexus program^25^. The TRB repertoires of these people were profiled using the immunoSEQ assay (Adaptive Biotechnologies) similar to the TRB repertoires of the discovery cohort. The second validation dataset was derived from 64 healthy controls and 154 people with PSC from Oslo University Hospital, Norway. For these samples, RNA was extracted from PAXgene tubes. Subsequently, 200 ng of RNA was used to profile the T cell repertoire using the Human TCR RNA Multiplex kit with UMI (MiLaboratories) following the manufacturer’s protocol. After sample demultiplexing, MIXCR (v.4.6.0)^61^ was used for identifying TRB clonotypes from the generated sequencing reads.

### Pathogen enrichment analysis

The analysis was performed using a hypergeometric test that accommodates four different variables, the total number of clonotypes, the number of clonotypes successfully annotated in a given database and the total number of clonotypes available for a particular pathogen in the database. Finally, the number of clonotypes annotated to be derived from a particular pathogen. Clonotypes with multiple antigenic specificities in the annotation database were dropped before the enrichment analysis.

### ECOCluster clonotype burden

ECOClusters are generated from TCRs with a known HLA association^32^, allowing us to ignore some instances of TCR cross-reactivity between HLAs. When calculating the clonotype burden for the ECOCluster TCRs, we counted clonotypes that are associated with HLAs encoded by the sample donor. We then divided this count by the total number of clonotypes observed in the sample to reach the ECOCluster burden.

### PhIP–seq immunoprecipitation, library preparation and sequencing

As a first step and to ensure a uniform antibody input for immunoprecipitation, each serum sample IgG antibody concentration was measured by a standard sandwich enzyme-linked immunosorbent assay (ELISA; primary antibody: goat anti-human IgG-UNLB, SB-2040-01; secondary antibody: goat F(ab')2 anti-human IgG-HRP, SB-2042-05). Three antigen libraries were used for performing PhIP–seq, namely, a microbiota library containing 244,000 variants^35^ a library composite of 100,000 peptides derived from environmental and allergic proteins^62^ and a library containing 13,192 antigenic peptides from different corona viral antigens^63^. The optimal ratio of phages and antibodies per reaction was determined beforehand and seemed to be a 4,000-fold coverage of phages per variant and 2 µg of antibodies^35^. First, a 96-deep-well plate (Greiner Bio-One, cat. no. 780270) was blocked with PhIP–seq blocking buffer (980 µl, Dulbecco’s phosphate-buffered saline (DPBS) with 3% BSA and 0.05% Tween 20) overnight at 4 °C on a rotator. Phages were then resuspended in extraction buffer (20 mM Tris-HCl, 100 mM NaCl, 6 mM MgSO_4_, pH 8.0, with 2 g l^−1^ BSA) to ensure a 4,000-fold coverage per variant. Resuspended phages were centrifuged for 2 h at 4 °C, at 3,000*g* to remove any cell debris or precipitate that could interfere with the assay. Bacteriophages (980 µl) were transferred to the preblocked 96-deep-well plate. Serum IgG antibodies (2 µg) were added to the bacteriophages. The plate was sealed with a 96-well sealing mat (Axygen, cat. no. AM-2ML-SQ) and incubated overnight at 4 °C on a rotator. A full-skirted 96-well PCR plate (Azenta Framestar, cat. no. 4ti-0960/W) preblocked with 150 µl of PhIP–seq blocking buffer (DPBS with 3% BSA and 0.05% Tween 20) overnight at 4 °C was prepared for the washing step. Protein A/G-coated magnetic beads (1:1 mixture of Invitrogen Dynabeads Protein A, cat. no. 10008D and Protein G, cat. no. 10009D) were washed three times with magnetic beads wash buffer (DPBS with 0.02% Tween 20) and resuspended in DPBS. Beads (40 µl) were added to bacteriophage-antibody mixtures, the plate was sealed with a 96-well sealing mat, and incubated for 4h at 4 °C on a rotator to ensure peptide–antibody complex formation. After 4 h, the plate was centrifuged at 900*g* for 2 min at room temperature before removing most of the supernatant. Beads were resuspended in the leftover (~100 µl) volume and transferred to the preblocked 96-well PCR plate. Beads were then washed twice with 145 µl of immunoprecipitation wash buffer (50 mM Tris-HCl, 150 mM NaCl, 0.1% IGEPAL CA630, pH 7.5). After the last wash step, the supernatant was removed, and the beads were stored at −20 °C until further processing. For pooled Illumina amplicon sequencing, several PCRs were performed with Q5 polymerase (New England Biolabs, cat. no. M0493L) according to the manufacturer’s recommendations. The following primer pairs were used: for PCR1, tcgtcggcagcgtcagatgtgtataagagacagGTTACTCGAGTGCGGCCGCAAGC and gtctcgtgggctcggagatgtgtataagagacagATGCTCGGGGATCCGAATTC; for PCR2, 10-nt unique dual index primers purchased from IDT; for PCR3 (of PCR2 pools), AATGATACGGCGACCACCGA and CAAGCAGAAGACGGCATACGA). PCR3 products were cut from an agarose gel and purified twice (1× QIAquick gel extraction kit, cat. no. 28704; 1× QIAquick PCR purification kit, cat. no. 28106; both according to manufacturer’s manual). Sequencing was done on an Illumina NovaSeq machine (custom primers for R1: ttactcgagtgcggccgcaagctttca; for R2: tgtgtataagagacagatgctcggggatccgaattct; R1/R2 44/31 nt).

### PhIP–seq data preprocessing

Pooled samples were sequenced with Illumina Novaseq 6000 paired read sequencing with 75 bp (read 1) and 25 bp (read 2). Raw reads were mapped to all three antigen libraries^35,62,63^, and the counts per peptide were normalized by comparing them with the base oligopeptide levels in the initial library (sequenced before immunoprecipitation)^35^. The generated count matrices were modeled using a generalized Poisson distribution, the two parameters, that is, λ and θ of this distribution, were estimated by analyzing the relationship between number of reads per variants in the library before immunoprecipitation and after immunoprecipitation but without incubation with serum. The probability of observing a particular count of reads against a particular antigen in the combined bacteriophage library was calculated using the fitted generalized Poisson model. Finally, Bonferroni correction was used to corrected for multiple testing. Subsequently, the fold-change of normalized peptide counts was computed to measure the level of enrichment. Information about fold-change levels, adjusted *P* values, read counts and presence–absence scores for each peptide were saved in separate matrix-like tables and used for downstream analysis. Before the association analysis, we filtered out samples with less than 250 bound antigens as well as samples with more than 3,000 antigens, because samples with either a very high or a very low number of bound-antigens might bias our statistical analyses.

### Isolation of liver-infiltrating mononuclear cells

Liver-infiltrating mononuclear cells (LIMCs) were isolated from liver tissue collected from recipient liver explants collected after liver transplantation^64^. Briefly, 50–100-g tissue sections were cut into 1–2 mm^3^ pieces, washed three to four times with PBS and resuspended in digestion media (RPMI 1640, 3% FBS, collagenase type 1A (VWR International)) for 30 min at 37 °C. Digested tissue was homogenized mechanically using a Stomacher400 circulator (Seward) and washed repeatedly with PBS through 63 μm nylon filters. Washed tissue was discarded and filtered cell suspensions were spun at 600*g* for 5 min. Wash supernatant was discarded and cell pellets were washed an additional three to four times with PBS to remove debris. Following the final wash, cell pellets were resuspended in PBS, and LIMCs were isolated by density centrifugation using Lympholyte-H (Cedarlane). Collected LIMCs were pelleted, resuspended in CryoStor cryopreservation medium (Sigma–Aldrich) at 5–25 × 10^6^ cells per aliquot, and stored in liquid nitrogen.

### Enrichment of hepatic B cells for single-cell RNA sequencing

Frozen LIMC aliquots were thawed quickly in 37 °C RPMI 1640 supplemented with 5% FBS and washed to remove cryopreservation medium. LIMCs were counted and resuspended at the recommended concentration for EasySep human Pan B cell negative-bead enrichment (StemCell Technologies), and B cells were enriched following the manufacturer’s protocol. To remove dead and dying cells postenrichment, B cells were stained with live/dead Zombie NIR dye (BioLegend) and sorted using a FACSMelody (BD Biosciences) before single-cell VDJ B cell receptor sequencing.

### Single-cell VDJ B cell receptor sequencing, alignment and annotation

Single-cell VDJ B cell receptor sequencing (BCR-seq) was performed on sorted liver B cells using Chromium Next GEM Single Cell 5′Library kit v.1.1 (10x Genomics) following the manufacturer’s user guide CG000207 Rev E. Pooled BCR cDNA libraries were barcoded, multiplexed and sequenced on a NovaSeq (Illumina) SP 200 cycle flow cell at 5,000 read pairs per cell. Sequencing reads were demultiplexed, aligned and annotated to the human genome reference GRCh38 (GENCODE v.32/Ensembl98) using Cell Ranger v.6.1.2 (10x Genomics) and BCR-seq was analyzed using Loupe VDJ Browser v.4.0.0 (10x Genomics).

### Generation of monoclonal antibodies

CDR3 BCR sequences obtained from single-cell VDJ BCR-seq were synthesized into monoclonal antibodies using high throughput (HTP-gene-to-antibody production service (GenScript)). Briefly, CDR3 sequences of expanded B cell clonotypes were cloned into pcDNA3.4 plasmids coexpressing human IgG1 kappa or human IgG1 lambda and transfected into the HD293F mammalian expression system. Monoclonal antibodies were purified using AmMag Protein A magnetic beads and resuspended in PBS and 50% glycerol for long-term storage.

### Antigen-specific T cell enrichment with HLA-B*08:01 tetramers

Freshly isolated or thawed peripheral blood mononuclear cells (PBMC) from HLA-B*08-positive healthy donors and people with PSC were blocked with anti-FcR reagent (Miltenyi) and stained first with a mix of PE-labeled HLA-B*08:01 tetramers loaded with RAKFKQLL (BZLF1) and FLRGRAYGL (EBNA3) peptides (immunAware APS). Samples were further stained with a cocktail of antibodies including anti-CD4-BV510 (clone OKT4, BioLegend), anti-CD8a-BV711 (clone RPA-T8, BioLegend), anti-CD3-BV785 (clone UCHT1, BioLegend), anti-CCR7-BV421 (clone G043H7, BioLegend), anti-CD45RA-Pe/Cy7 (clone HI100, BioLegend) and a live/dead dye 7AAD. Antigen-specific CD8^+^ T cells were gated as 7AAD^−^CD8^+^CD3^+^tetramer^+^ and sorted directly into RLT lysis buffer (Qiagen) using the SONY MA900 cell sorter.

### In vitro stimulation for induction of CD137 expression and antigen-specific CD8 T cell enrichment

Freshly isolated PBMCs were plated in 24-well plate at density of 7 × 10^6^ cells per well in 1 ml RPMI 1640, supplemented with 5% FCS and 1% penicillin-streptomycin. PepTivators EBV BZLF1 and EBNA1 (Miltenyi) consisting of 15-mer peptides covering EBV’s BZLF1 and EBNA1 proteins were used as antigens either separately (at peptide concentration 0.6 μM each) or as mix (at peptide concentration 0.3 μM each). Negative control wells without antigen stimulation were set for each donor and processed in parallel. After 16 h of incubation at 37 °C in 5% CO_2_, cells were harvested, stained with anti-CD137-PE antibody (clone 4B4-1, Miltenyi) and labeled further with anti-PE magnetic microbeads (Miltenyi). Antigen-specific CD137^+^ cells were enriched with positive magnetic selection on MS-columns (Miltenyi). CD137^+^ enriched cells were stained on columns with an antibody cocktail including anti-CD4-BV510 (clone OKT4, BioLegend), anti-CD8a-BV711 (clone RPA-T8, BioLegend), anti-CD3-BV785 (clone UCHT1, BioLegend), anti-CCR7-BV421 (clone G043H7, BioLegend), anti-CD45RA-Pe/Cy7 (clone HI100, BioLegend), anti-CD69-FITC (clone FN50, BioLegend) and a live/dead dye 7AAD. Afterwards, the on-column staining cells were eluted from the columns and sorted on SONY MA900 cell sorter. Antigen-specific CD8^+^ T cells were gated as 7AAD^−^CD8^+^CD137^+^CD69^+^ and sorted directly into RLT lysis buffer (Qiagen). Negative control wells were analyzed in parallel.

### Profiling the TRB repertoire of EBV-specific clonotypes

RNA was extracted from tetramer-sorted antigen-stimulated T cells using the RNeasy Micro Kit (Qiagen, cat. no. 74104). All extracted RNA was used to profile the TRB repertoire using the Human TCR RNA Multiplex kit with UMI (MiLaboratories) following the manufacturer’s protocol. Prepared libraries were sequenced using an Illumina NextSeq 1000 P1 lane (2 × 150 bp), generating approximately 100 million reads, providing an average of 7–7.7 million reads per library. After sample demultiplexing, MiXCR (v.4.6.0)^61^ was used for identifying TRB clonotypes from the generated sequencing reads.

### Generation and cultivation of EBV-transformed LCLs and EBV-specific T cell lines

To generate EBV-transformed lymphoblastoid cell lines (LCLs), frozen PBMCs were thawed from selected individuals with PSC (*n* = 4), centrifuged at 300*g* for 5 min at 4 °C in RPMI 1640 (Gibco), containing 10% heat-inactivated FCS (Gibco) and 1% penicillin-streptomycin (PenStrep; Gibco), and B cells were negatively selected using a Pan B isolation kit (Miltenyi). B cells were treated with CpG ODN 2006 (InvivoGen) and EBV supernatant for 1.5 h at 37 °C, 5% CO_2_. Next, the B cells were cultured with irradiated healthy donor feeder cells in medium consisting of RPMI 1640 (Gibco), 1% penicillin-streptomycin (PenStrep; Gibco), 5% human AB serum, 2% GlutaMAX (Gibco), 1% Minimum Essential Medium Non-Essential Amino Acids (Gibco) and 1% sodium pyruvate (Gibco), in addition to 1.25 μg ml^−1^ CpG ODN 2006 at 37 °C, 5% CO_2_. During expansion, the medium was replaced, and the cells were split as needed. To generate EBV-specific T cell lines, flowthrough from the negatively selected B cells was used according to the manufacturer's instructions. Cells were expanded in CTL medium (CTL-M) comprising of RPMI 1640 (Sigma–Aldrich), 1% penicillin-streptomycin (PenStrep; Gibco) and 10% human AB serum, supplemented with IL-4 (400 U ml^−1^), IL-7 (10 ng ml^−1^) and 0.1 µg ml^−1^ peptide pools: EBV consensus (Miltenyi), EBV-BZLF1, EBV-BMRF1 and EBV-EBNA1 (all JPT Peptide Technologies). Cells were pulsed with peptides in 1 ml in a 24-well G-Rex device (Wilson Wolf) overnight, then supplemented with CTL-M containing IL-4 and IL-7, and cultured for 14 days. Next, EBV-specific T cell lines were harvested and further expanded for 14 days in CTL-M containing irradiated T2 cells, irradiated PBMCs and 30 ng ml^−1^ OKT-3. The next day, 50 U ml^−1^ IL-2 was added, and again every third day. On day 4, culture medium was replaced completely, and medium was changed subsequently as needed. After 14 days of T2 expansion, T cell lines were frozen down in CTL-M containing 20% dimethyl sulfoxide (Sigma–Aldrich).

### Interferon-γ ELISpot

To investigate the antigenic response of EBV-specific T cell lines, expanded T cell lines were thawed and 3 × 10^5^ T cells were added to anti-interferon-γ-coated (1 mg ml^−1^; clone 1-D1K, Mabtech) ELISpot MultiScreen_HTS_ 96-well filter plates (Merck). T cells were stimulated overnight with autologous EBV-LCLs in an effector:target (E:T) ratio of 1:1 and allogenic HLA-B:08-matched EBV-LCLs and nontransformed B cells in an E:T ratio of 5:1. Allogenic HLA-B:08-matched donors were used due to limited material. Plates were developed using interferon-γ antibody (Mabtech), streptavidin-alkaline phosphatase (Mabtech), and 1-Step NBT/BCIP (Thermo Fisher Scientific). Spots were counted on dried plates with an AID ELISpot Reader.

### TriNetX

The TriNetX US Collaborative Network provides access to anonymized electronic medical records of 116 million people, from 65 US healthcare organizations. Medical health records of people <80 years of age from the US collaborative network were used to assemble cohorts of people diagnosed with infectious mononucleosis, excluding those who had also been diagnosed with *Chlamydia*, and people diagnosed with *Chlamydia* but not infectious mononucleosis. Propensity score matching for sex and age at the index between the two cohorts resulted in 124,383 people per cohort. We chose *Chlamydia* for negative control matching as it presents with a similar age distribution to that of infectious mononucleosis. Analytics for cohort outcomes, including people with outcome before the time window, were performed on the TriNetX Live Advanced Analytics dashboard with only aggregate results from infectious mononucleosis and control cohorts being surfaced and returned to the platform.

### Protein structural localization of BFRF3 in the EBV capsid

The EBV capsid reconstruction from cryogenic electron microscopy was retrieved from the Protein Databank (PDB ID 6w19)^56^ and visualized with Mol* 3D-Viewer. For analyzing the BFRF3/MCP hexon complex only, chains A–F (MCP) and Q–V (BFRF3) were extracted from the PDB file and visualized with PyMOL.

### Statistics and reproducibility

We used a cross-sectional study design in which we profiled the immune repertoire of people with PSC and healthy controls. Sample size for this study was based on availability considerations, as we did not recruit new participants but used stored biomaterial from previously recruited people with PSC and healthy blood donors. To have sufficient power to detect even small effects, we used biomaterial of all individuals with PSC that were available to us and of a larger number of healthy blood donors. The available sample sizes of *n* = 504 individuals with PSC and *n* = 904 healthy controls provide at least 95% power to detect differences of *d* ≥ 0.2 (standardized effect size) with a Mann–Whitney test (G*Power v.3.1). We also performed PhIP–seq on 120 people with PSC and 202 people without PSC. Some samples were removed during the quality control of the assays, mainly from the PhIP–seq dataset (those with fewer than 250 bound antigens or more than 3,000 bound antigens); no other metrics were used to exclude samples from any of these analyses. PSC-associated clonotypes were identified using a one-sided Fisher’s exact test and the association between infectious mononucleosis and time to PSC onset in the TriNetX dataset was calculated using the Cox proportional hazard model. Pathogen enrichment analysis was conducted using the hypergeometric test. Independent groups (for example, individuals with PSC versus healthy controls or carriers versus noncarriers of specific HLA alleles) were compared regarding different quantitative variables (for example, burden of PSC-associated clonotypes) by the two-sided nonparametric Mann–Whitney–Wilcoxon test as most variables were expected to be skewed and non-Gaussian. Association between such quantitative variables (for example, number of unique clonotypes and burden of PSC-associated clonotypes) was assessed by the nonparametric Spearman correlation coefficient.

### Reporting summary

Further information on research design is available in the Nature Portfolio Reporting Summary linked to this article.

## Online content

Any methods, additional references, Nature Portfolio reporting summaries, source data, extended data, supplementary information, acknowledgements, peer review information; details of author contributions and competing interests; and statements of data and code availability are available at 10.1038/s41591-025-03692-w.

## Supplementary information

Reporting Summary

Supplementary Table 1 The PSC-associated clonotypes identified from the T cell repertoires of the discovery cohort containing 504 individuals with PSC and 904 healthy controls.

Supplementary Table 2 The PSC-associated clonotypes identified by seeded clustering using the 136 PSC-associated clonotypes identified from the incidence analysis.

Supplementary Table 3 PSC-associated clonotypes that were targeting EBV antigens as define by the MIRA assay.

## Figures and Tables

**Extended Data Table 1:**
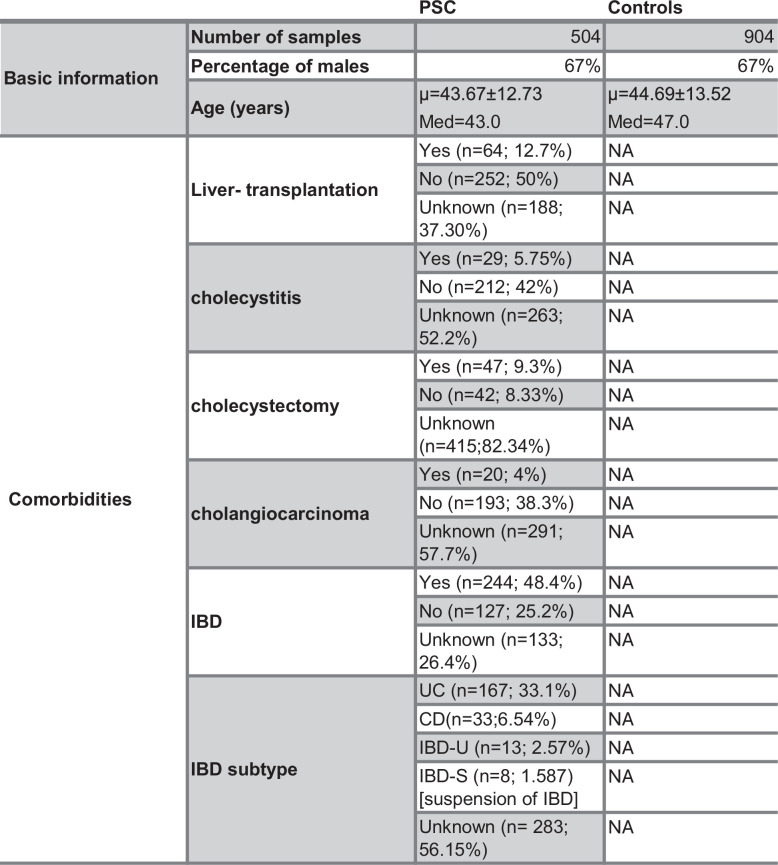
The clinical and phenotypic properties of the study cohort included in the TCR sequencing experiment

## Data Availability

The TCR-seq results of 904 healthy participants and 504 participants with PSC are available via Zenodo at 10.5281/zenodo.14989127 (ref. ^65^). The PhIP–seq data of participants with PSC are available via Zenodo at 10.5281/zenodo.14989837 (ref. ^66^) and for healthy controls at 10.5281/zenodo.14989788 (ref. ^67^). Due to GDPR and consent restrictions, clinical data from these people can be obtained by submitting a project application to the popgen v.2.0 Network (https://portal.popgen.de/) with a processing time of approximately 2 months. The first validation dataset, namely, the US-based SPARC IBD dataset, is available upon approved application to the Crohnʼs and Colitis Foundation IBD Plexus Program (https://www.crohnscolitisfoundation.org/ibd-plexus). Regarding the second validation dataset containing the TRB repertoire of people with PSC as well as healthy controls from Norway, institutional data privacy regulations prohibit deposition of individual level data to public repositories. Participant written consent also does not cover public sharing of data for use for unknown purposes. Upon contact with T.H.K. (t.h.karlsen@medisin.uio.no) an institutional data transfer agreement can be established and data shared if the aims of data use are covered by ethical approval and patient consent. The procedure will involve an update to the ethical approval as well as review by legal departments at both institutions, and the process will typically take 1–2 months from initial contact.

## References

[1] Karlsen, T. H., Folseraas, T., Thorburn, D. & Vesterhus, M. Primary sclerosing cholangitis; a comprehensive review. *J. Hepatol.* **67**, 1298–1323 (2017). 28802875 10.1016/j.jhep.2017.07.022

[2] Carbone, M. et al. Liver transplantation for primary sclerosing cholangitis (PSC) with or without inflammatory bowel disease (IBD)—a European Society of Organ Transplantation (ESOT) consensus statement. *Transpl. Int.* **36**, 11729 (2023). 37841645 10.3389/ti.2023.11729 PMC10570452

[3] Chazouilleres, O. et al. EASL clinical practice guidelines on sclerosing cholangitis. *J. Hepatol.* **77**, 761–806 (2022). 35738507 10.1016/j.jhep.2022.05.011

[4] van Munster, K. N. et al. Disease burden in primary sclerosing cholangitis in the Netherlands: a long-term follow-up study. *Liver Int.* **43**, 639–648 (2023). 36328957 10.1111/liv.15471

[5] Ji, S.-G. et al. Genome-wide association study of primary sclerosing cholangitis identifies new risk loci and quantifies the genetic relationship with inflammatory bowel disease. *Nat. Genet.* **49**, 269–273 (2017). 27992413 10.1038/ng.3745 PMC5540332

[6] Ellinghaus, D. et al. Analysis of five chronic inflammatory diseases identifies 27 new associations and highlights disease-specific patterns at shared loci. *Nat. Genet.* **48**, 510–518 (2016). 26974007 10.1038/ng.3528 PMC4848113

[7] Liu, J. Z. et al. Dense genotyping of immune-related disease regions identifies nine new risk loci for primary sclerosing cholangitis. *Nat. Genet.* **45**, 670–675 (2013). 23603763 10.1038/ng.2616 PMC3667736

[8] Næss, S. et al. Refinement of the MHC risk map in a Scandinavian primary sclerosing cholangitis population. *PLoS ONE* **9**, e114486 (2014). 25521205 10.1371/journal.pone.0114486 PMC4270690

[9] Karlsen, T. H. et al. Different HLA class II associations in ulcerative colitis patients with and without primary sclerosing cholangitis. *Genes Immun.* **8**, 275–278 (2007). 17301827 10.1038/sj.gene.6364377

[10] Probert, C. S. et al. Analysis of human common bile duct-associated T cells: evidence for oligoclonality, T cell clonal persistence, and epithelial cell recognition. *J. Immunol.* **158**, 1941–1948 (1997). 9029136

[11] Poch, T. et al. Single-cell atlas of hepatic T cells reveals expansion of liver-resident naive-like CD4^+^ T cells in primary sclerosing cholangitis. *J. Hepatol.* **75**, 414–423 (2021). 33774059 10.1016/j.jhep.2021.03.016 PMC8310924

[12] Bo, X., Broome, U., Remberger, M. & Sumitran-Holgersson, S. Tumour necrosis factor α impairs function of liver derived T lymphocytes and natural killer cells in patients with primary sclerosing cholangitis. *Gut* **49**, 131–141 (2001). 11413121 10.1136/gut.49.1.131 PMC1728361

[13] Liaskou, E. et al. Loss of CD28 expression by liver-infiltrating T cells contributes to pathogenesis of primary sclerosing cholangitis. *Gastroenterology* **147**, 221–232.e7 (2014). 24726754 10.1053/j.gastro.2014.04.003 PMC4961260

[14] Roth, D. B. V(D)J recombination: mechanism, errors, and fidelity. *Microbiol. Spectr.*10.1128/microbiolspec.MDNA3-0041-2014 (2014). 10.1128/microbiolspec.MDNA3-0041-2014 PMC5089068 26104458

[15] Robins, H. Immunosequencing: applications of immune repertoire deep sequencing. *Curr. Opin. Immunol.* **25**, 646–652 (2013). 24140071 10.1016/j.coi.2013.09.017

[16] Carlson, C. S. et al. Using synthetic templates to design an unbiased multiplex PCR assay. *Nat. Commun.* **4**, 2680 (2013). 24157944 10.1038/ncomms3680

[17] Emerson, R. O. et al. Immunosequencing identifies signatures of cytomegalovirus exposure history and HLA-mediated effects on the T cell repertoire. *Nat. Genet.* **49**, 659–665 (2017). 28369038 10.1038/ng.3822

[18] Snyder, T. M. et al. Magnitude and dynamics of the T-cell response to SARS-CoV-2 infection at both individual and population levels. *Front. Immunol.*10.3389/fimmu.2024.1488860 (2025). 10.3389/fimmu.2024.1488860 PMC11747429 39840037

[19] Greissl, J. et al. Immunosequencing of the T-cell receptor repertoire reveals signatures specific for identification and characterization of early Lyme disease. Preprint at *medRxiv*10.1101/2021.07.30.21261353 (2022).

[20] Komech, E. A. et al. CD8^+^ T cells with characteristic T cell receptor beta motif are detected in blood and expanded in synovial fluid of ankylosing spondylitis patients. *Rheumatology* **57**, 1097–1104 (2018). 29481668 10.1093/rheumatology/kex517

[21] Faham, M. et al. Discovery of T cell receptor β motifs specific to HLA–B27–positive ankylosing spondylitis by deep repertoire sequence analysis. *Arthritis Rheumatol.* **69**, 774–784 (2017). 28002888 10.1002/art.40028

[22] Britanova, O. V. et al. Targeted depletion of TRBV9^+^ T cells as immunotherapy in a patient with ankylosing spondylitis. *Nat. Med.* **29**, 2731–2736 (2023). 37872223 10.1038/s41591-023-02613-z PMC10667094

[23] Larman, H. B. et al. Autoantigen discovery with a synthetic human peptidome. *Nat. Biotechnol.* **29**, 535–541 (2011). 21602805 10.1038/nbt.1856 PMC4169279

[24] Mohan, D. et al. PhIP–seq characterization of serum antibodies using oligonucleotide-encoded peptidomes. *Nat. Protoc.* **13**, 1958–1978 (2018). 30190553 10.1038/s41596-018-0025-6 PMC6568263

[25] Raffals, L. E. et al. The development and initial findings of a study of a prospective adult research cohort with inflammatory bowel disease (SPARC IBD). *Inflamm. Bowel Dis.* **28**, 192–199 (2022). 34436563 10.1093/ibd/izab071 PMC9013198

[26] Liaskou, E. et al. High‐throughput T‐cell receptor sequencing across chronic liver diseases reveals distinct disease‐associated repertoires. *Hepatology* **63**, 1608–19 (2016). 26257205 10.1002/hep.28116

[27] Henriksen, E. K. K. et al. Gut and liver T-cells of common clonal origin in primary sclerosing cholangitis-inflammatory bowel disease. *J. Hepatol.* **66**, 116–122 (2017). 27647428 10.1016/j.jhep.2016.09.002

[28] Zahid, H. J. et al. Large-scale statistical mapping of T-cell receptor β sequences to human leukocyte antigens. Preprint at *bioRxiv*10.1101/2024.04.01.587617 (2024).

[29] Goncharov, M. et al. VDJdb in the pandemic era: a compendium of T cell receptors specific for SARS-CoV-2. *Nat. Methods* **19**, 1017–1019 (2022). 35970936 10.1038/s41592-022-01578-0

[30] Tickotsky, N., Sagiv, T., Prilusky, J., Shifrut, E. & Friedman, N. McPAS-TCR: a manually curated catalogue of pathology-associated T cell receptor sequences. *Bioinformatics* **33**, 2924–2929 (2017). 28481982 10.1093/bioinformatics/btx286

[31] Klinger, M. et al. Multiplex identification of antigen-specific T cell receptors using a combination of immune assays and immune receptor sequencing. *PLoS ONE* **10**, e0141561 (2015). 26509579 10.1371/journal.pone.0141561 PMC4624875

[32] May, D. H. et al. Identifying immune signatures of common exposures through co-occurrence of T-cell receptors in tens of thousands of donors. Preprint at *bioRxiv*10.1101/2024.03.26.583354 (2024).

[33] Pesesky, M. et al. Antigen-driven expansion of public clonal T cell populations in inflammatory bowel diseases. *J. Crohns Colitis*10.1093/ecco-jcc/jjaf048 (2025). 10.1093/ecco-jcc/jjaf048 40121186

[34] Andreu-Sánchez, S. et al. Phage display sequencing reveals that genetic, environmental, and intrinsic factors influence variation of human antibody epitope repertoire. *Immunity* **56**, 1376–1392.e8 (2023). 37164013 10.1016/j.immuni.2023.04.003 PMC12166656

[35] Vogl, T. et al. Population-wide diversity and stability of serum antibody epitope repertoires against human microbiota. *Nat. Med.* **27**, 1442–1450 (2021). 34282338 10.1038/s41591-021-01409-3

[36] Vogl, T. et al. Systemic antibody responses against human microbiota flagellins are overrepresented in chronic fatigue syndrome patients. *Sci. Adv.* **8**, eabq2422 (2024). 10.1126/sciadv.abq2422 PMC11580831 36149952

[37] Bourgonje, A. R. et al. Phage-display immunoprecipitation sequencing of the antibody epitope repertoire in inflammatory bowel disease reveals distinct antibody signatures. *Immunity* **56**, 1393–1409.e6 (2023). 37164015 10.1016/j.immuni.2023.04.017

[38] Lindsey, J. W. Antibodies to the Epstein-Barr virus proteins BFRF3 and BRRF2 cross-react with human proteins. *J. Neuroimmunol.* **310**, 131–134 (2017). 28778437 10.1016/j.jneuroim.2017.07.013

[39] Fechtner, S. et al. Antibody responses to Epstein–Barr virus in the preclinical period of rheumatoid arthritis suggest the presence of increased viral reactivation cycles. *Arthritis Rheumatol.* **74**, 597–603 (2022). 34605217 10.1002/art.41994 PMC8957485

[40] Ascherio, A. et al. Epstein–Barr virus antibodies and risk of multiple sclerosis: a prospective study. *JAMA* **286**, 3083–3088 (2001). 11754673 10.1001/jama.286.24.3083

[41] Pasoto, S. G. et al. EBV reactivation serological profile in primary Sjögren’s syndrome: an underlying trigger of active articular involvement? *Rheumatol. Int.* **33**, 1149–1157 (2013). 22955798 10.1007/s00296-012-2504-3

[42] Leung, A. K. C. et al. Infectious mononucleosis: an updated review. *Curr. Pediatr. Rev.* **20**, 305–322 (2024). 37526456 10.2174/1573396320666230801091558

[43] Robinson, W. H., Younis, S., Love, Z. Z., Steinman, L. & Lanz, T. V. Epstein–Barr virus as a potentiator of autoimmune diseases. *Nat. Rev. Rheumatol.* **20**, 729–740 (2024). 39390260 10.1038/s41584-024-01167-9

[44] Bjornevik, K. et al. Longitudinal analysis reveals high prevalence of Epstein–Barr virus associated with multiple sclerosis. *Science* **375**, 296–301 (2022). 35025605 10.1126/science.abj8222

[45] Moon, U. Y. et al. Patients with systemic lupus erythematosus have abnormally elevated Epstein–Barr virus load in blood. *Arthritis Res. Ther.* **6**, R295 (2004). 15225364 10.1186/ar1181 PMC464871

[46] Whittingham, S., McNeilage, L. J. & Mackay, I. R. Epstein–Barr virus as an etiological agent in primary Sjögren’s syndrome. *Med. Hypotheses* **22**, 373–386 (1987). 3035352 10.1016/0306-9877(87)90033-8

[47] Jog, N. R. et al. Association of Epstein–Barr virus serological reactivation with transitioning to systemic lupus erythematosus in at-risk individuals. *Ann. Rheum. Dis.* **78**, 1235 (2019). 31217170 10.1136/annrheumdis-2019-215361 PMC6692217

[48] McClain, M. T. et al. Early events in lupus humoral autoimmunity suggest initiation through molecular mimicry. *Nat. Med.* **11**, 85–89 (2005). 15619631 10.1038/nm1167

[49] Csorba, K. et al. Anti-C1q antibodies as occurring in systemic lupus erythematosus could be induced by an Epstein–Barr virus-derived antigenic site. *Front. Immunol.* **10**, 2619 (2019). 31787984 10.3389/fimmu.2019.02619 PMC6853867

[50] Lanz, T. V. et al. Clonally expanded B cells in multiple sclerosis bind EBV EBNA1 and GlialCAM. *Nature* **603**, 321–327 (2022). 35073561 10.1038/s41586-022-04432-7 PMC9382663

[51] Morrison, T. E., Mauser, A., Wong, A., Ting, J. P.-Y. & Kenney, S. C. Inhibition of IFN-γ signaling by an Epstein–Barr virus immediate-early protein. *Immunity* **15**, 787–799 (2001). 11728340 10.1016/s1074-7613(01)00226-6

[52] Tsurumi, T., Fujita, M. & Kudoh, A. Latent and lytic Epstein–Barr virus replication strategies. *Rev. Med. Virol.* **15**, 3–15 (2005). 15386591 10.1002/rmv.441

[53] Salamun, S. G. et al. The Epstein–Barr virus BMRF1 protein activates transcription and inhibits the DNA damage response by binding NuRD. *J. Virol.* **93**, e01070-19 (2019). 31462557 10.1128/JVI.01070-19 PMC6819917

[54] Perdue, C. L., Eick-Cost, A. A. & Rubertone, M. V. A brief description of the operation of the DoD serum repository. *Mil. Med.* **180**, 10–12 (2015). 26444888 10.7205/MILMED-D-14-00739

[55] Ebert, A. C. et al. Risk of inflammatory bowel disease following hospitalisation with infectious mononucleosis: nationwide cohort study from Denmark. *Nat. Commun.* **15**, 8383 (2024). 39333475 10.1038/s41467-024-52195-8 PMC11437054

[56] Liu, W. et al. Structures of capsid and capsid-associated tegument complex inside the Epstein–Barr virus. *Nat. Microbiol.* **5**, 1285–1298 (2020). 32719506 10.1038/s41564-020-0758-1 PMC7546529

[57] Gittelman, R. M. et al. Longitudinal analysis of T cell receptor repertoires reveals shared patterns of antigen-specific response to SARS-CoV-2 infection. *JCI Insight* **7**, e151849 (2022). 35439174 10.1172/jci.insight.151849 PMC9220833

[58] Pruessmann, W. et al. Molecular analysis of primary melanoma T cells identifies patients at risk for metastatic recurrence. *Nat. Cancer* **1**, 197–209 (2020). 33305293 10.1038/s43018-019-0019-5 PMC7725220

[59] Degenhardt, F. et al. Construction and benchmarking of a multi-ethnic reference panel for the imputation of HLA class I and II alleles. *Hum. Mol. Genet.* **28**, 2078–2092 (2019). 30590525 10.1093/hmg/ddy443 PMC6548229

[60] Degenhardt, F. et al. Trans-ethnic analysis of the human leukocyte antigen region for ulcerative colitis reveals shared but also ethnicity-specific disease associations. *Hum. Mol. Genet.* **30**, 356–369 (2021). 33555323 10.1093/hmg/ddab017 PMC8098114

[61] Bolotin, D. A. et al. MiXCR: software for comprehensive adaptive immunity profiling. *Nat. Methods* **12**, 380–381 (2015). 25924071 10.1038/nmeth.3364

[62] Leviatan, S. et al. Allergenic food protein consumption is associated with systemic IgG antibody responses in non-allergic individuals. *Immunity* **55**, 2454–2469.e6 (2022). 36473469 10.1016/j.immuni.2022.11.004 PMC12103637

[63] Klompus, S. et al. Cross-reactive antibodies against human coronaviruses and the animal coronavirome suggest diagnostics for future zoonotic spillovers. *Sci. Immunol.* **6**, eabe9950 (2021). 34326184 10.1126/sciimmunol.abe9950 PMC9267281

[64] Chung, B. K. et al. Phenotyping and auto-antibody production by liver-infiltrating B cells in primary sclerosing cholangitis and primary biliary cholangitis. *J. Autoimmun.* **77**, 45–54 (2017). 27784538 10.1016/j.jaut.2016.10.003

[65] ElAbd, H. et al. The TRB repertoires for ElAbd et al.; 2025; Nat Med. *Zenodo*10.5281/zenodo.14989127 (2025).

[66] ElAbd, H. et al. The PhIP-Seq repertoires of the 120 individuals with PSC from ElAbd et al.; 2025; Nat Med manuscript. *Zenodo*10.5281/zenodo.14989837 (2025).

[67] ElAbd, H. et al. The PhIP-Seq repertoires of the 202 healthy controls from ElAbd et al.; 2025; Nat Med manuscript. *Zenodo*10.5281/zenodo.14989788 (2025).

