# Supplementary material for: T and B cell responses against Epstein–Barr virus in primary sclerosing cholangitis
Source: Nat Med. 2025 Jun 11;31(7):2306–16. doi: 10.1038/s41591-025-03692-w (PMC12283410; doi:10.1038/s41591-025-03692-w)
Supplement: Supplementary file 1 — Reporting Summary [file 41591_2025_3692_MOESM1_ESM.pdf]

nature portfolio

Corresponding author(s): Hesham ElAbd, Andre Franke

Last updated by author(s): Mar 6, 2025

Reporting Summary

Nature Portfolio wishes to improve the reproducibility of the work that we publish. This form provides structure for consistency and transparency in reporting. For further information on Nature Portfolio policies, see our Editorial Policies and the Editorial Policy Checklist.

Statistics

For all statistical analyses, confirm that the following items are present in the figure legend, table legend, main text, or Methods section.

n/a	Confirmed	
☐	☒	The exact sample size (*n*) for each experimental group/condition, given as a discrete number and unit of measurement
☐	☒	A statement on whether measurements were taken from distinct samples or whether the same sample was measured repeatedly
☐	☒	The statistical test(s) used AND whether they are one- or two-sided<br>*Only common tests should be described solely by name; describe more complex techniques in the Methods section.*
☐	☒	A description of all covariates tested
☐	☒	A description of any assumptions or corrections, such as tests of normality and adjustment for multiple comparisons
☐	☒	A full description of the statistical parameters including central tendency (e.g. means) or other basic estimates (e.g. regression coefficient) AND variation (e.g. standard deviation) or associated estimates of uncertainty (e.g. confidence intervals)
☐	☒	For null hypothesis testing, the test statistic (e.g. *F*, *t*, *r*) with confidence intervals, effect sizes, degrees of freedom and *P* value noted<br>*Give P values as exact values whenever suitable.*
☒	☐	For Bayesian analysis, information on the choice of priors and Markov chain Monte Carlo settings
☒	☐	For hierarchical and complex designs, identification of the appropriate level for tests and full reporting of outcomes
☒	☐	Estimates of effect sizes (e.g. Cohen's *d*, Pearson's *r*), indicating how they were calculated

Our web collection on statistics for biologists contains articles on many of the points above.

Software and code

Policy information about availability of computer code

Data collection	No software were used for data collection.
Data analysis	Data analysis was mainly conducted using different Python 3 libraries and frameworks such as Pandas (1.5.3), SciPy (1.13.0) and NumPy (1.25.2) in addition to miXCR (v4.6.0). Data visualization was also conducted using Matplotlib (3.8.0) and Seaborn (0.11.2). For BCR-Seq data analysis, Cell Ranger v.6.1.2 (10X Genomics) and Loupe VDJ Browser v4.0.0 (10X Genomics) were used.

For manuscripts utilizing custom algorithms or software that are central to the research but not yet described in published literature, software must be made available to editors and reviewers. We strongly encourage code deposition in a community repository (e.g. GitHub). See the Nature Portfolio guidelines for submitting code & software for further information.

Data

Policy information about availability of data

All manuscripts must include a data availability statement. This statement should provide the following information, where applicable:

- Accession codes, unique identifiers, or web links for publicly available datasets
- A description of any restrictions on data availability
- For clinical datasets or third party data, please ensure that the statement adheres to our policy

The TCR-seq results of 904 healthy participants and 504 participants with PSC are available via Zenodo at https://doi.org/10.5281/zenodo.14989127. The PhIP–seq data of participants with PSC are available via Zenodo at https://doi.org/10.5281/zenodo.14989837 and for healthy controls at https://doi.org/10.5281/

zenodo.14989788. Due to GDPR and consent restrictions, clinical data for these people can be obtained by submitting a project application to the popgen v.2.0 Network (https://portal.popgen.de/) with a processing time of approximately 2 months. The first validation dataset, namely, the US-based SPARC IBD dataset, is available upon approved application to the Crohn's and Colitis Foundation IBD Plexus Program (https://www.crohnscolitisfoundation.org/ibd-plexus). Regarding the second validation dataset containing the TRB repertoires of people with PSC as well as healthy controls from Norway, institutional data privacy regulations prohibit deposition of individual level data to public repositories. Participant written consent also does not cover public sharing of data for use for unknown purposes. Upon contact with T.H.K. (t.h.karlsen@medisin.uio.no) an institutional data transfer agreement can be established and data shared if the aims of data use are covered by ethical approval and patient consent. The procedure will involve an update to the ethical approval as well as review by legal departments at both institutions, and the process will typically take 1–2 months from initial contact.

Research involving human participants, their data, or biological material

Policy information about studies with human participants or human data. See also policy information about sex, gender (identity/presentation), and sexual orientation and race, ethnicity and racism.

Reporting on sex and gender

We utilized large-scale immune-repertoire profiling data using TCR-Seq for 504 individuals with PSC and 904 controls. In addition, we performed PhIP-Seq on 120 samples and 202 healthy controls. For the TCR-Seq cohorts, we had a male ratio of 67% to reflect the male-predominance observed in the individuals with PSC (here also 67%). For samples measured with PhIP-Seq we had a 47% and 57% males in the PSC and control samples, respectively. Self-reported sex was previously validated using SNP-array-based sex predictions for the same samples. We studied sex-specific effects in the burden analysis of disease-associated clonotypes shown in Extended Data Figure 3 where the burden of PSC-associated clonotypes was comparable in individuals with PSC and and in controls.

Reporting on race, ethnicity, or other socially relevant groupings

There was no reporting on race or ethnicity on the study, as most samples were collected from individuals with European-ancestry.

Population characteristics

The population characteristics are described in table S1. In brief, samples used for TCR-Seq had the following characteristics:
1. Age mean=43.67±12.73 for PSC patients and 44.69±13.52 for healthy controls.
2. both sample panels had an equal ratio of males and females with 67% of the cohorts being male
For samples included in PhIP-Seq analysis, the study population had the following properties
1. median age= 44.65 ±13.24 for PSC and 46.8±8.47 for healthy controls
2. 47.5% of the PSC cohort consisted of males and 57% of the controls were male

Recruitment

Healthy controls were recruited via the local transfusion medicine department from blood donors, while individuals with PSC were recruited via the local biobank PopGen. For the current study, we have not actively recruited participant but used stored biomaterial from previously recruited PSC patients and healthy blood donors. The samples were selected to have matching age and sex distribution whenever possible.

Ethics oversight

The study has been approved by the ethical committee at the University of Kiel under the following ethical votes: D441/16, D474/12, A161/08, A103/14, and A148/14. Also, by the Regional Committees for Medical and Health Research Ethics of South-Eastern Norway (reference numbers 18221 and 13381). A written informed consent was collected from all participants prior to the beginning of the study. For isolating monoclonal antibodies from liver samples, fresh PSC and PBC liver explants were collected at Queen Elizabeth Hospital in Birmingham, United Kingdom, and at Oslo University Hospital Rikshospitalet in Oslo, Norway. As per the Declaration of Helsinki, written informed consent was obtained from participants in accordance with local research ethics committee approvals in Birmingham (LREC #06-Q2702-61) and Regional Committees for Medical and Health Research Ethics of South-Eastern Norway (reference numbers 13381, 18221 and 15368).

Note that full information on the approval of the study protocol must also be provided in the manuscript.

Field-specific reporting

Please select the one below that is the best fit for your research. If you are not sure, read the appropriate sections before making your selection.

☒ Life sciences ☐ Behavioural & social sciences ☐ Ecological, evolutionary & environmental sciences

For a reference copy of the document with all sections, see nature.com/documents/nr-reporting-summary-flat.pdf

Life sciences study design

All studies must disclose on these points even when the disclosure is negative.

Sample size

Sample size for this study was based on availability considerations, as we did not recruit new individuals but used stored biomaterial from previously recruited individuals with PSC and healthy blood donors. In order to have sufficient power to detect even small effects, we used biomaterial of all individuals with PCS that were available to us and of a larger number of healthy blood donors. The available sample sizes of n=504 individuals with PSC and n=904 healthy controls provide at least 95% power to detect differences of d>=0.2 (standardized effect size) with a Mann-Whitney test (G*Power 3.1).

Data exclusions

While some samples were removed during the quality control of these assays, mainly from the PhIP-Seq dataset were samples with fewer than 250 bound antigens or more than 3,000 bound antigens we removed, no other metrics was used to exclude samples from any of these analyses.

Replication

We replicated our findings at the T cell repertoire level using two independent datasets. First, the "A Study of a Prospective Adult Research

Replication	Cohort with IBD" cohort of the US-based Crohn's & Colitis Foundation IBD Plexus program (SPARC IBD) which contains 2,487 individuals with IBD only and 73 individuals with PSC-IBD. Second, a PSC cohort from Norway that contains 154 individuals with PSC and 64 healthy controls.
Randomization	We utilized a cross-sectional study design where we profiled the immune repertoire of individuals with PSC and healthy controls.Healthy controls were selected to match the age and sex distribution of individuals with PSC whenever possible.
Blinding	Given our case-control study, blinding was only used during data generation where samples from healthy controls and individuals with PSC were randomized and barcoded.

Reporting for specific materials, systems and methods

We require information from authors about some types of materials, experimental systems and methods used in many studies. Here, indicate whether each material, system or method listed is relevant to your study. If you are not sure if a list item applies to your research, read the appropriate section before selecting a response.

Materials & experimental systems

n/a	Involved in the study
☐	☒ Antibodies
☐	☒ Eukaryotic cell lines
☒	☐ Palaeontology and archaeology
☒	☐ Animals and other organisms
☒	☐ Clinical data
☒	☐ Dual use research of concern
☒	☐ Plants

Methods

n/a	Involved in the study
☒	☐ ChIP-seq
☒	☐ Flow cytometry
☒	☐ MRI-based neuroimaging

Antibodies

Antibodies used	anti-CD4-BV510 (clone OKT4, biolegend), anti-CD8a-BV711 (clone RPA-T8, biolegend), anti-CD3-BV785 (clone UCHT1, biolegend), anti-CCR7-BV421 (clone G043H7, biolegend), anti-CD45RA-Pe/Cy7 (clone HI100, biolegend), anti-CD137-PE antibody (clone 4B4-1, Miltenyi), anti-IFN-γ-coated (1 mg/ml; clone 1-D1K, Mabtech), Goat Anti-Human IgG-UNLB (SB-2040-01), Goat F(ab')2 Anti-Human IgG-HRP (SB-2042-05)
Validation	1. the validation of anti-CD4-BV510 (https://www.biolegend.com/en-ie/products/purified-anti-human-cd4-antibody-3650?GroupID=BLG590)<br>2. the validation of anti-CD8a-BV711 (https://www.biolegend.com/en-gb/products/brilliant-violet-711-anti-human-cd8a-antibody-7929)<br>3. the validation of anti-CD3-BV785 (https://www.biolegend.com/fr-fr/products/brilliant-violet-785-anti-human-cd3-antibody-14454?GroupID=BLG5900)<br>4. the validation of anti-CCR7-BV421 (https://www.biolegend.com/fr-lu/products/brilliant-violet-421-anti-human-cd197-ccr7-antibody-7497)<br>5. the validation of anti-CD45RA-Pe/Cy7 (https://www.biolegend.com/de-at/products/pe-cyanine7-anti-human-cd45ra-antibody-7055?GroupID=GROUP658)<br>6. the validation of anti-CD137-PE antibody (clone 4B4-1, Miltenyi) (https://www.miltenyibiotec.com/DE-en/products/cd137-antibody-anti-human-4b4-1.html#conjugate=vio-bright-fitc:size=100-tests-in-200-ul)<br>7. the validation of anti-IFN-γ-coated (1 mg/ml; clone 1-D1K, Mabtech) (https://www.mabtech.com/products/anti-human-ifn-g-mab-1-d1k-unconjugated-3420-3)<br>8. the validation of Goat Anti-Human IgG-UNLB (https://www.southernbiotech.com/goat-anti-human-igg-unlb-2040-01)<br>9. the validation of Goat F(ab')2 Anti-Human IgG-HRP (https://www.southernbiotech.com/goat-f-ab-2-anti-human-igg-hrp-2042-05)

Eukaryotic cell lines

Policy information about cell lines and Sex and Gender in Research

Cell line source(s)	EBV-transformed lymphoblastoid cell line and EBV-specific T cell lines were generated from PBMC isolated from four individuals with PSC according to the protocol described in the online methods.
Authentication	Cells were not authenticated as B cells are not viable after weeks if they are not EBV transformed so the fact that they spontaneously proliferate after weeks indicates their transformation
Mycoplasma contamination	cell lines tested negative for mycoplasma contamination
Commonly misidentified lines (See ICLAC register)	NA

Plants

Seed stocks	*Report on the source of all seed stocks or other plant material used. If applicable, state the seed stock centre and catalogue number. If plant specimens were collected from the field, describe the collection location, date and sampling procedures.*
Novel plant genotypes	*Describe the methods by which all novel plant genotypes were produced. This includes those generated by transgenic approaches, gene editing, chemical/radiation-based mutagenesis and hybridization. For transgenic lines, describe the transformation method, the number of independent lines analyzed and the generation upon which experiments were performed. For gene-edited lines, describe the editor used, the endogenous sequence targeted for editing, the targeting guide RNA sequence (if applicable) and how the editor was applied.*
Authentication	*Describe any authentication procedures for each seed stock used or novel genotype generated. Describe any experiments used to assess the effect of a mutation and, where applicable, how potential secondary effects (e.g. second site T-DNA insertions, mosiacism, off-target gene editing) were examined.*
