# Supplementary material for: T and B cell responses against Epstein–Barr virus in primary sclerosing cholangitis
Source: Nat Med. 2025 Jun 11;31(7):2306–16. doi: 10.1038/s41591-025-03692-w (PMC12283410; doi:10.1038/s41591-025-03692-w)
Supplement: Supplementary file 2 — The PSC-associated clonotypes identified from the T cell repertoires of the discovery cohort containing 504 individuals with PSC and 904 healthy controls. [file 41591_2025_3692_MOESM2_ESM.pdf]

CDR3 amino acid	V gene segment	J gene segment
CSVGSGEGYEQYF	TCRBV29-01	TCRBJ02-07
CASSLDSGSGNTIYF	TCRBV05-05	TCRBJ01-03
CASSLALAGTYNEQFF	TCRBV07-09	TCRBJ02-01
CASSLDGQAYEQYF	TCRBV07-02	TCRBJ02-07
CAWSWGGEQYF	TCRBV30-01	TCRBJ02-07
CSARAGGRSYEQYF	TCRBV20	TCRBJ02-07
CASSLEGRGSYEQYF	TCRBV11-02	TCRBJ02-07
CASSQDVQYGYTF	TCRBV04-03	TCRBJ01-02
CASSRGPNYEQYF	TCRBV07-06	TCRBJ02-07
CASSLGGQPYGYTF	TCRBV27-01	TCRBJ01-02
CASSQGPNYEQYF	TCRBV07-06	TCRBJ02-07
CASSEGQGEQPQHF	TCRBV02-01	TCRBJ01-05
CASSPTGLQETQYF	TCRBV07-03	TCRBJ02-05
CSAAEGTNTEAFF	TCRBV29-01	TCRBJ01-01
CASSQGSRSGNTIYF	TCRBV03-01/03-02	TCRBJ01-03
CSVEGGRGTDTQYF	TCRBV29-01	TCRBJ02-03
CASSDQGEDQPQHF	TCRBV10-02	TCRBJ01-05
CSALDGDTEAFF	TCRBV20	TCRBJ01-01
CASSLGGIPYGYTF	TCRBV27-01	TCRBJ01-02
CASSPGQGEGYEQYF	TCRBV04-01	TCRBJ02-07
CSVDAGFQETQYF	TCRBV29-01	TCRBJ02-05
CASSLGSSHQPQHF	TCRBV07-02	TCRBJ01-05
CSARDREVTGELFF	TCRBV20	TCRBJ02-02
CASSGDTQYF	TCRBV07	TCRBJ02-03
CASSDLNSPLHF	TCRBV27-01	TCRBJ01-06
CASSLVGGRSYEQYF	TCRBV05-01	TCRBJ02-07
CASSWGTSGRASQETQYF	TCRBV05-01	TCRBJ02-05
CASSPEGQGSPLHF	TCRBV18-01	TCRBJ01-06
CASSLGRDPTGELFF	TCRBV28-01	TCRBJ02-02
CASSPGPNYEQYF	TCRBV07	TCRBJ02-07
CASSLGGENTIYF	TCRBV05-01	TCRBJ01-03
CSAPPRGDSPLHF	TCRBV20	TCRBJ01-06
CASSSGQAYEQYF	TCRBV07-08	TCRBJ02-07
CASSDSAGYGYTF	TCRBV02-01	TCRBJ01-02
CASSLWGRGTDTQYF	TCRBV28-01	TCRBJ02-03
CASSQEGGSSTDTQYF	TCRBV04-03	TCRBJ02-03
CASSLGGLPYGYTF	TCRBV27-01	TCRBJ01-02
CASSLTSSTDTQYF	TCRBV11-03	TCRBJ02-03
CASSIIDRDTEAFF	TCRBV19-01	TCRBJ01-01
CASSHTDRNYGYTF	TCRBV04-01	TCRBJ01-02

CASSLTGVSGNTIYF	TCRBV11-03	TCRBJ01-03
CASSPRGGLTDTQYF	TCRBV03-01/03-02	TCRBJ02-03
CASSLSWGEQYF	TCRBV07-02	TCRBJ02-07
CASSRGGLSYEQYF	TCRBV14-01	TCRBJ02-07
CSTNTDTQYF	TCRBV29-01	TCRBJ02-03
CASSRGPNYEQYF	TCRBV07	TCRBJ02-07
CSVGPDSTDTQYF	TCRBV29-01	TCRBJ02-03
CASSFSGQNTEAFF	TCRBV12	TCRBJ01-01
CASSSTSGGRGETQYF	TCRBV05-04	TCRBJ02-05
CASSFGGRSSGANVLTF	TCRBV05-04	TCRBJ02-06
CASSEYQEGTEAFF	TCRBV25-01	TCRBJ01-01
CSAHPEAFF	TCRBV20-01	TCRBJ01-01
CASSVGGAVNTEAFF	TCRBV09-01	TCRBJ01-01
CASSLSRGGADGYTF	TCRBV27-01	TCRBJ01-02
CASSYGGQPYGYTF	TCRBV06-05	TCRBJ01-02
CASSLASSNEQFF	TCRBV28-01	TCRBJ02-01
CSARNRDYGYTF	TCRBV20	TCRBJ01-02
CSVGSGEDNEQFF	TCRBV29-01	TCRBJ02-01
CASSFKGDQPQHF	TCRBV07-02	TCRBJ01-05
CASSFPLGTDTQYF	TCRBV28-01	TCRBJ02-03
CASSDGTSGGASETQYF	TCRBV09-01	TCRBJ02-05
CASSLEANTDTQYF	TCRBV05-01	TCRBJ02-03
CASSPRYSNQPQHF	TCRBV11-02	TCRBJ01-05
CAWRTGFNTEAFF	TCRBV30-01	TCRBJ01-01
CASSLERYEKLFF	TCRBV07-02	TCRBJ01-04
CASSGGAGSTDTQYF	TCRBV02-01	TCRBJ02-03
CASSYSPQGGYEQYF	TCRBV06-05	TCRBJ02-07
CASSLVNPYEQYF	TCRBV07-02	TCRBJ02-07
CSARVSSGGGNEQFF	TCRBV20	TCRBJ02-01
CASSPEGQGSGYTF	TCRBV18-01	TCRBJ01-02
CATGTDLETQYF	TCRBV24-01	TCRBJ02-05
CASSLGPNYEQYF	TCRBV07	TCRBJ02-07
CASSLTGTGGRQPQHF	TCRBV07-02	TCRBJ01-05
CASSVGASGSLGETQYF	TCRBV09-01	TCRBJ02-05
CASSIAGLLSSYNEQFF	TCRBV19-01	TCRBJ02-01
CASSPGPNYEQYF	TCRBV07-06	TCRBJ02-07
CSVARGSEAFF	TCRBV29-01	TCRBJ01-01
CASKFQETQYF	TCRBV02-01	TCRBJ02-05
CASSSGPNYEQYF	TCRBV07	TCRBJ02-07
CASSVETGATGELFF	TCRBV09-01	TCRBJ02-02
CASSLVAAGTEAFF	TCRBV05-01	TCRBJ01-01

CASSPGTGEGYEQYF	TCRBV04-01	TCRBJ02-07
CSVDTDTQYF	TCRBV29-01	TCRBJ02-03
CASSLSLGETQYF	TCRBV07-02	TCRBJ02-05
CASSSLNTEAFF	TCRBV27-01	TCRBJ01-01
CASSQGPNYEQYF	TCRBV07	TCRBJ02-07
CSARRQGPGNTIYF	TCRBV20	TCRBJ01-03
CSTDTDTQYF	TCRBV29-01	TCRBJ02-03
CASSLGGYPYGYTF	TCRBV27-01	TCRBJ01-02
CASSLSLAGETQYF	TCRBV11-02	TCRBJ02-05
CASSGDRDSPLHF	TCRBV10-02	TCRBJ01-06
CASSLTLAGGPNEQFF	TCRBV05-01	TCRBJ02-01
CASRGRGYNEQFF	TCRBV19-01	TCRBJ02-01
CASGRLANTGELFF	TCRBV28-01	TCRBJ02-02
CASSHPDSYGYTF	TCRBV04-03	TCRBJ01-02
CASSPRGPSTDTQYF	TCRBV18-01	TCRBJ02-03
CASSTSRGSGNTIYF	TCRBV04-01	TCRBJ01-03
CASSSRNSGNTIYF	TCRBV28-01	TCRBJ01-03
CASSLRLAGPYEQYF	TCRBV11-02	TCRBJ02-07
CASSQDLAGATDTQYF	TCRBV04-03	TCRBJ02-03
CSAGGGTGKNIQYF	TCRBV20	TCRBJ02-04
CASSLSPGMNTEAFF	TCRBV04-01	TCRBJ01-01
CSARGSSGGRETQYF	TCRBV20	TCRBJ02-05
CASSYPRGGENSPLHF	TCRBV06-05	TCRBJ01-06
CSANRGEGYTF	TCRBV20-01	TCRBJ01-02
CASSQGNYGYTF	TCRBV11-03	TCRBJ01-02
CASSPRLAGGDTQYF	TCRBV12	TCRBJ02-03
CSSRGLYNEQFF	TCRBV20	TCRBJ02-01
CASSLQGYSNQPQHF	TCRBV05-08	TCRBJ01-05
CASSSGPNYEQYF	TCRBV07-06	TCRBJ02-07
CASSLLQGNEKLFF	TCRBV12	TCRBJ01-04
CASRFAGELFF	TCRBV27-01	TCRBJ02-02
CASSFLAGDTGELFF	TCRBV05-01	TCRBJ02-02
CASSSRGGGEKLFF	TCRBV05-01	TCRBJ01-04
CASSLLQGAYEQYF	TCRBV07-02	TCRBJ02-07
CASSLGPNYEQYF	TCRBV07-06	TCRBJ02-07
CASSHLNNEQFF	TCRBV03-01/03-02	TCRBJ02-01
CASSFWGRDTQYF	TCRBV05-01	TCRBJ02-03
CASSQVDRDTQYF	TCRBV04-03	TCRBJ02-03
CASSPQGGPGEQYF	TCRBV18-01	TCRBJ02-07
CASSLVAGGRETQYF	TCRBV05-06	TCRBJ02-05
CASSVETGGTGELFF	TCRBV09-01	TCRBJ02-02

CASSRTVNYGYTF	TCRBV07-02	TCRBJ01-02
CASSHRGTGELFF	TCRBV28-01	TCRBJ02-02
CASSLGQAYEQYF	TCRBV07-08	TCRBJ02-07
CASSVGLGELFF	TCRBV09-01	TCRBJ02-02
CSARDRGSENTGELFF	TCRBV20	TCRBJ02-02
CASSPPGQGVGQPQHF	TCRBV12	TCRBJ01-05
CASSFVQGGTEAFF	TCRBV07-02	TCRBJ01-01
CSARDRGTENTGELFF	TCRBV20	TCRBJ02-02
CASSFEGGNIQYF	TCRBV07-02	TCRBJ02-04
CASSLNTGGQETQYF	TCRBV05-01	TCRBJ02-05
CASSLSLAGAYNEQFF	TCRBV07-09	TCRBJ02-01
CASSPNRGGTEAFF	TCRBV07-03	TCRBJ01-01
CASSWGQGERGYTF	TCRBV05-04	TCRBJ01-02
CASSVGQGRSYNSPLHF	TCRBV09-01	TCRBJ01-06
