# Supplementary material for: T and B cell responses against Epstein–Barr virus in primary sclerosing cholangitis
Source: Nat Med. 2025 Jun 11;31(7):2306–16. doi: 10.1038/s41591-025-03692-w (PMC12283410; doi:10.1038/s41591-025-03692-w)
Supplement: Supplementary file 3 — The PSC-associated clonotypes identified by seeded clustering using the 136 PSC-associated clonotypes identified from the incidence analysis. [file 41591_2025_3692_MOESM3_ESM.pdf]

CDR3 amino acid	V gene segment	J gene segment
CSARNRDYGYTF	TCRBV20	TCRBJ01-02
CASSLNTGGTETQYF	TCRBV05-01	TCRBJ02-05
CASSLTLAGGANEQFF	TCRBV05-01	TCRBJ02-01
CASSLPQAYEQYF	TCRBV07-08	TCRBJ02-07
CASSRGGNYEQYF	TCRBV07	TCRBJ02-07
CASSIIDRDTEAFF	TCRBV19-01	TCRBJ01-01
CAWSFGGEQYF	TCRBV30-01	TCRBJ02-07
CASSLSLAGETQYF	TCRBV11-02	TCRBJ02-05
CASSAGQGEGYEQYF	TCRBV04-01	TCRBJ02-07
CASSGGAGSTDTQYF	TCRBV02-01	TCRBJ02-03
CASSHPDSYGYTF	TCRBV04-03	TCRBJ01-02
CASSHYNNEQFF	TCRBV03-01/03-02	TCRBJ02-01
CSVARGSKAFF	TCRBV29-01	TCRBJ01-01
CASKGDTQYF	TCRBV07	TCRBJ02-03
CASSLRLAGPYEQYF	TCRBV11-02	TCRBJ02-07
CSAGAGGRSYEQYF	TCRBV20	TCRBJ02-07
CASSPRAGLTDTQYF	TCRBV03-01/03-02	TCRBJ02-03
CASSKKGDQPQHF	TCRBV07-02	TCRBJ01-05
CASSPGQGETYEQYF	TCRBV04-01	TCRBJ02-07
CASSFWGQNTEAFF	TCRBV12	TCRBJ01-01
CASSSGPYYEQYF	TCRBV07	TCRBJ02-07
CASSPPGQGRGQPQHF	TCRBV12	TCRBJ01-05
CASSQRNYGYTF	TCRBV11-03	TCRBJ01-02
CASSPVQGGTEAFF	TCRBV07-02	TCRBJ01-01
CASSVGQGVSYNSPLHF	TCRBV09-01	TCRBJ01-06
CASSHLWNEQFF	TCRBV03-01/03-02	TCRBJ02-01
CASSRRGGLTDTQYF	TCRBV03-01/03-02	TCRBJ02-03
CSVGSGEDYEQYF	TCRBV29-01	TCRBJ02-07
CSCDTDTQYF	TCRBV29-01	TCRBJ02-03
CSTHTDTQYF	TCRBV29-01	TCRBJ02-03
CASSVDTQYF	TCRBV07	TCRBJ02-03
CASSPQGGPGEQYF	TCRBV18-01	TCRBJ02-07
CASSSALAGTYNEQFF	TCRBV07-09	TCRBJ02-01
CAWSWRGEQYF	TCRBV30-01	TCRBJ02-07
CSARAGGRPYEQYF	TCRBV20	TCRBJ02-07
CASSRTVAYGYTF	TCRBV07-02	TCRBJ01-02
CASSPGPVYEQYF	TCRBV07	TCRBJ02-07
CASSPGTGEPYEQYF	TCRBV04-01	TCRBJ02-07
CASSLETGGTGELFF	TCRBV09-01	TCRBJ02-02
CASSVWTGGTGELFF	TCRBV09-01	TCRBJ02-02

CASRGLGYNEQFF	TCRBV19-01	TCRBJ02-01
CASSVETGPTGELFF	TCRBV09-01	TCRBJ02-02
CASSRTVNYGYTF	TCRBV07-02	TCRBJ01-02
CASSQGFRSGNTIYF	TCRBV03-01/03-02	TCRBJ01-03
CSSRGLYNEQFF	TCRBV20	TCRBJ02-01
CASSPVGGRSYEQYF	TCRBV05-01	TCRBJ02-07
CASSKGGQPYGYTF	TCRBV06-05	TCRBJ01-02
CSARAGGASYEQYF	TCRBV20	TCRBJ02-07
CASSLQGFSNQPQHF	TCRBV05-08	TCRBJ01-05
CSVEGGRDTDTQYF	TCRBV29-01	TCRBJ02-03
CASSLSLAGAYNEQFF	TCRBV07-09	TCRBJ02-01
CASSLASGSGNTIYF	TCRBV05-05	TCRBJ01-03
CASQGDTQYF	TCRBV07	TCRBJ02-03
CSARDRGRENTGELFF	TCRBV20	TCRBJ02-02
CSVGAGEGYEQYF	TCRBV29-01	TCRBJ02-07
CASSLTSSTDTQYF	TCRBV11-03	TCRBJ02-03
CASSLSPGLNTEAFF	TCRBV04-01	TCRBJ01-01
CASSLLQGNEKLFF	TCRBV12	TCRBJ01-04
CSAWDGDTEAFF	TCRBV20	TCRBJ01-01
CASSIQGLLSSYNEQFF	TCRBV19-01	TCRBJ02-01
CASAFQETQYF	TCRBV02-01	TCRBJ02-05
CASSPNRGGTEAFF	TCRBV07-03	TCRBJ01-01
CASSPRGMSTDTQYF	TCRBV18-01	TCRBJ02-03
CASSGNTGGQETQYF	TCRBV05-01	TCRBJ02-05
CASSVSQGRSYNSPLHF	TCRBV09-01	TCRBJ01-06
CASSLAGGRSYEQYF	TCRBV05-01	TCRBJ02-07
CASSSGGLPYGYTF	TCRBV27-01	TCRBJ01-02
CASSLLWGEQYF	TCRBV07-02	TCRBJ02-07
CASSLLQVNEKLFF	TCRBV12	TCRBJ01-04
CSVGSGEGGEQYF	TCRBV29-01	TCRBJ02-07
CASSWGQGEGYEQYF	TCRBV04-01	TCRBJ02-07
CASSLVAAGTEAFF	TCRBV05-01	TCRBJ01-01
CASSREGGNIQYF	TCRBV07-02	TCRBJ02-04
CASSLGGQDYGYTF	TCRBV27-01	TCRBJ01-02
CASSPGQGSGYEQYF	TCRBV04-01	TCRBJ02-07
CASSEIQGEQPQHF	TCRBV02-01	TCRBJ01-05
CASSFKVDQPQHF	TCRBV07-02	TCRBJ01-05
CSARAGGMSYEQYF	TCRBV20	TCRBJ02-07
CASSYPRTGENSPLHF	TCRBV06-05	TCRBJ01-06
CASSHENNEQFF	TCRBV03-01/03-02	TCRBJ02-01
CASRGREYNEQFF	TCRBV19-01	TCRBJ02-01

CASSLAGTGGRQPQHF	TCRBV07-02	TCRBJ01-05
CASSYVGQPYGYTF	TCRBV06-05	TCRBJ01-02
CASSLTSATDTQYF	TCRBV11-03	TCRBJ02-03
CRTDTDTQYF	TCRBV29-01	TCRBJ02-03
CASSLGQQYEQYF	TCRBV07-08	TCRBJ02-07
CASSPGQGAGYEQYF	TCRBV04-01	TCRBJ02-07
CASSLIGRGTDTQYF	TCRBV28-01	TCRBJ02-03
CASSSGPFYEQYF	TCRBV07	TCRBJ02-07
CSEDTDTQYF	TCRBV29-01	TCRBJ02-03
CASSQGSRVGNTIYF	TCRBV03-01/03-02	TCRBJ01-03
CASSPGPTYEQYF	TCRBV07-06	TCRBJ02-07
CASSLTGYSGNTIYF	TCRBV11-03	TCRBJ01-03
CASSLSPGMNTEAFF	TCRBV04-01	TCRBJ01-01
CSARDREHTGELFF	TCRBV20	TCRBJ02-02
CASSLSIGEQYF	TCRBV07-02	TCRBJ02-07
CASSYGGVPYGYTF	TCRBV06-05	TCRBJ01-02
CASSTGPNYEQYF	TCRBV07-06	TCRBJ02-07
CSVARPSEAFF	TCRBV29-01	TCRBJ01-01
CASSLMAGGRETQYF	TCRBV05-06	TCRBJ02-05
CASSPEGQGNGYTF	TCRBV18-01	TCRBJ01-02
CASSPGTNYEQYF	TCRBV07-06	TCRBJ02-07
CASSTSRGSGNTIYF	TCRBV04-01	TCRBJ01-03
CASSLGGLPGGYTF	TCRBV27-01	TCRBJ01-02
CASQGRGYNEQFF	TCRBV19-01	TCRBJ02-01
CSTEGGRGTDTQYF	TCRBV29-01	TCRBJ02-03
CASSPGGLSYEQYF	TCRBV14-01	TCRBJ02-07
CASSDLNSPLHF	TCRBV27-01	TCRBJ01-06
CASSPDTQYF	TCRBV07	TCRBJ02-03
CSARDSGTENTGELFF	TCRBV20	TCRBJ02-02
CASSYPRGGENSPLHF	TCRBV06-05	TCRBJ01-06
CSAIRGEGYTF	TCRBV20-01	TCRBJ01-02
CASSLLQGAYEQYF	TCRBV07-02	TCRBJ02-07
CASSHGQGRSYNSPLHF	TCRBV09-01	TCRBJ01-06
CASSLVAWGTEAFF	TCRBV05-01	TCRBJ01-01
CSATDREVTGELFF	TCRBV20	TCRBJ02-02
CASSSGPLYEQYF	TCRBV07-06	TCRBJ02-07
CASKTQETQYF	TCRBV02-01	TCRBJ02-05
CSTDTDTQYF	TCRBV29-01	TCRBJ02-03
CASSVETGGTGELFF	TCRBV09-01	TCRBJ02-02
CASSQGPLYEQYF	TCRBV07-06	TCRBJ02-07
CASSLSGENTIYF	TCRBV05-01	TCRBJ01-03

CSARDIEVTGELFF	TCRBV20	TCRBJ02-02
CASSQGPNYEQYV	TCRBV07	TCRBJ02-07
CASSWLAGDTGELFF	TCRBV05-01	TCRBJ02-02
CASSVNTGGQETQYF	TCRBV05-01	TCRBJ02-05
CASSVGPGRSYNSPLHF	TCRBV09-01	TCRBJ01-06
CSARAGGRSYEQYF	TCRBV20	TCRBJ02-07
CSVGSGEGAEQYF	TCRBV29-01	TCRBJ02-07
CASSVGQGTSYNSPLHF	TCRBV09-01	TCRBJ01-06
CASSRGGLSYEQYF	TCRBV14-01	TCRBJ02-07
CASKFEETQYF	TCRBV02-01	TCRBJ02-05
CASSDSAGGGYTF	TCRBV02-01	TCRBJ01-02
CASSPGPYYEQYF	TCRBV07-06	TCRBJ02-07
CSARTQGPGNTIYF	TCRBV20	TCRBJ01-03
CASSFGGRDTQYF	TCRBV05-01	TCRBJ02-03
CASSLNQGGQETQYF	TCRBV05-01	TCRBJ02-05
CASSRPGLSYEQYF	TCRBV14-01	TCRBJ02-07
CASKVQETQYF	TCRBV02-01	TCRBJ02-05
CASSIGPNYEQYF	TCRBV07-06	TCRBJ02-07
CASSSRTSGNTIYF	TCRBV28-01	TCRBJ01-03
CASSPGVGEGYEQYF	TCRBV04-01	TCRBJ02-07
CSAARGTNTEAFF	TCRBV29-01	TCRBJ01-01
CASSRGPSYEQYF	TCRBV07	TCRBJ02-07
CASSLGGPPYGYTF	TCRBV27-01	TCRBJ01-02
CASSVGQAVNTEAFF	TCRBV09-01	TCRBJ01-01
CSVGSGEGYEQYF	TCRBV29-01	TCRBJ02-07
CASSRGRNYEQYF	TCRBV07	TCRBJ02-07
CASSLVLGGRETQYF	TCRBV05-06	TCRBJ02-05
CASSRSGLSYEQYF	TCRBV14-01	TCRBJ02-07
CASSWGTSGRGSQETQYF	TCRBV05-01	TCRBJ02-05
CASSYWGRDTQYF	TCRBV05-01	TCRBJ02-03
CASSFGQAYEQYF	TCRBV07-08	TCRBJ02-07
CASSVEPGGTGELFF	TCRBV09-01	TCRBJ02-02
CASSLTLSTDTQYF	TCRBV11-03	TCRBJ02-03
CASSLQLAGAYNEQFF	TCRBV07-09	TCRBJ02-01
CASRGAGYNEQFF	TCRBV19-01	TCRBJ02-01
CASSYGGYPYGYTF	TCRBV06-05	TCRBJ01-02
CSAPPTGDSPLHF	TCRBV20	TCRBJ01-06
CASSFSAGDTGELFF	TCRBV05-01	TCRBJ02-02
CSTQTDTQYF	TCRBV29-01	TCRBJ02-03
CSAGRGEGYTF	TCRBV20-01	TCRBJ01-02
CSADTDTQYF	TCRBV29-01	TCRBJ02-03

CSVEGGRHTDTQYF	TCRBV29-01	TCRBJ02-03
CSASEGTNTEAFF	TCRBV29-01	TCRBJ01-01
CASSLGGLDYGYTF	TCRBV27-01	TCRBJ01-02
CASKMQETQYF	TCRBV02-01	TCRBJ02-05
CSARNSDYGYTF	TCRBV20	TCRBJ01-02
CASSPRGFLTDTQYF	TCRBV03-01/03-02	TCRBJ02-03
CASSGRGPSTDTQYF	TCRBV18-01	TCRBJ02-03
CASSSRPSGNTIYF	TCRBV28-01	TCRBJ01-03
CASSPQGQGSGYTF	TCRBV18-01	TCRBJ01-02
CASSFVAGGRETQYF	TCRBV05-06	TCRBJ02-05
CASSPGTGGGYEQYF	TCRBV04-01	TCRBJ02-07
CASKFQETQYF	TCRBV02-01	TCRBJ02-05
CASSHGQGEGYEQYF	TCRBV04-01	TCRBJ02-07
CASSQGRNYEQYF	TCRBV07-06	TCRBJ02-07
CASSSGLNYEQYF	TCRBV07	TCRBJ02-07
CSAFDGDTEAFF	TCRBV20	TCRBJ01-01
CASSILAGDTGELFF	TCRBV05-01	TCRBJ02-02
CASSLVANTDTQYF	TCRBV05-01	TCRBJ02-03
CSAAEGTNTEAFF	TCRBV29-01	TCRBJ01-01
CASSLSTGMNTEAFF	TCRBV04-01	TCRBJ01-01
CASSLSRGGSDGYTF	TCRBV27-01	TCRBJ01-02
CASSLQGYGNQPQHF	TCRBV05-08	TCRBJ01-05
CASSPETGGTGELFF	TCRBV09-01	TCRBJ02-02
CASSFTGLQETQYF	TCRBV07-03	TCRBJ02-05
CSVEAGFQETQYF	TCRBV29-01	TCRBJ02-05
CASSQDSAGATDTQYF	TCRBV04-03	TCRBJ02-03
CSVARDSEAFF	TCRBV29-01	TCRBJ01-01
CASSVQLGELFF	TCRBV09-01	TCRBJ02-02
CASAGDTQYF	TCRBV07	TCRBJ02-03
CASSDAAGYGYTF	TCRBV02-01	TCRBJ01-02
CSAREGTNTEAFF	TCRBV29-01	TCRBJ01-01
CSIDTDTQYF	TCRBV29-01	TCRBJ02-03
CASSSGRNYEQYF	TCRBV07	TCRBJ02-07
CASSFQAGDTGELFF	TCRBV05-01	TCRBJ02-02
CASSSVGGRSYEQYF	TCRBV05-01	TCRBJ02-07
CASSRGTNYEQYF	TCRBV07-06	TCRBJ02-07
CSARVNSGGGNEQFF	TCRBV20	TCRBJ02-01
CASSSGTNYEQYF	TCRBV07-06	TCRBJ02-07
CASSSNTGGQETQYF	TCRBV05-01	TCRBJ02-05
CASSLEGRGVYEQYF	TCRBV11-02	TCRBJ02-07
CASSHLNSPLHF	TCRBV27-01	TCRBJ01-06

CSVARGSEAFF	TCRBV29-01	TCRBJ01-01
CSVGSGRGYEQYF	TCRBV29-01	TCRBJ02-07
CASSDTNSPLHF	TCRBV27-01	TCRBJ01-06
CSVGSGVGYEQYF	TCRBV29-01	TCRBJ02-07
CASSSRQAYEQYF	TCRBV07-08	TCRBJ02-07
CASSRSRGSGNTIYF	TCRBV04-01	TCRBJ01-03
CASSLQGSSNQPQHF	TCRBV05-08	TCRBJ01-05
CASSFEGGNIQYF	TCRBV07-02	TCRBJ02-04
CASSPGPQYEQYF	TCRBV07	TCRBJ02-07
CASSFSGRDTQYF	TCRBV05-01	TCRBJ02-03
CASSRGQNYEQYF	TCRBV07	TCRBJ02-07
CSAARQGPGNTIYF	TCRBV20	TCRBJ01-03
CASSPRGGNTDTQYF	TCRBV03-01/03-02	TCRBJ02-03
CASSQLLAGATDTQYF	TCRBV04-03	TCRBJ02-03
CATGTDQETQYF	TCRBV24-01	TCRBJ02-05
CSARALGRSYEQYF	TCRBV20	TCRBJ02-07
CASGSLNTEAFF	TCRBV27-01	TCRBJ01-01
CASSWRNSGNTIYF	TCRBV28-01	TCRBJ01-03
CSARVSSGSGNEQFF	TCRBV20	TCRBJ02-01
CASSYGQGEGYEQYF	TCRBV04-01	TCRBJ02-07
CASSQVDRDTQYF	TCRBV04-03	TCRBJ02-03
CASSLVRGGRETQYF	TCRBV05-06	TCRBJ02-05
CASSRTVDYGYTF	TCRBV07-02	TCRBJ01-02
CASSLTLAGAYNEQFF	TCRBV07-09	TCRBJ02-01
CASSPTGFQETQYF	TCRBV07-03	TCRBJ02-05
CASSTGQGRSYNSPLHF	TCRBV09-01	TCRBJ01-06
CASSAGTSGGASETQYF	TCRBV09-01	TCRBJ02-05
CASSAQGYSNQPQHF	TCRBV05-08	TCRBJ01-05
CSARVSSGGGNEQFF	TCRBV20	TCRBJ02-01
CSVQGGRGTDTQYF	TCRBV29-01	TCRBJ02-03
CASSTTRGSGNTIYF	TCRBV04-01	TCRBJ01-03
CASSFWGQDTQYF	TCRBV05-01	TCRBJ02-03
CSVEIGRGTDTQYF	TCRBV29-01	TCRBJ02-03
CSVDTDTQYF	TCRBV29-01	TCRBJ02-03
CASSPGPSYEQYF	TCRBV07-06	TCRBJ02-07
CASSHLFNEQFF	TCRBV03-01/03-02	TCRBJ02-01
CSARDRGTENTGELFF	TCRBV20	TCRBJ02-02
CASSLNPGGQETQYF	TCRBV05-01	TCRBJ02-05
CASSPEAQGSPLHF	TCRBV18-01	TCRBJ01-06
CSARRQGPGNTIYF	TCRBV20	TCRBJ01-03
CASSPEGQGTGYTF	TCRBV18-01	TCRBJ01-02

CASSLALSGTYNEQFF	TCRBV07-09	TCRBJ02-01
CSAEGGRGTDTQYF	TCRBV29-01	TCRBJ02-03
CASSPGQGEGYEQYF	TCRBV04-01	TCRBJ02-07
CASSLTPSTDTQYF	TCRBV11-03	TCRBJ02-03
CSARGGGRSYEQYF	TCRBV20	TCRBJ02-07
CASSLIANTDTQYF	TCRBV05-01	TCRBJ02-03
CASSQDLAGNTDTQYF	TCRBV04-03	TCRBJ02-03
CASSLGGVNTIYF	TCRBV05-01	TCRBJ01-03
CASSLTGVSGNTIYF	TCRBV11-03	TCRBJ01-03
CAWSWGREQYF	TCRBV30-01	TCRBJ02-07
CASSYYGQPYGYTF	TCRBV06-05	TCRBJ01-02
CASSSGPRYEQYF	TCRBV07-06	TCRBJ02-07
CASSLHQGNEKLFF	TCRBV12	TCRBJ01-04
CASSLGGYPYGYTF	TCRBV27-01	TCRBJ01-02
CASSSFNTEAFF	TCRBV27-01	TCRBJ01-01
CSARAVGRSYEQYF	TCRBV20	TCRBJ02-07
CSARNQDYGYTF	TCRBV20	TCRBJ01-02
CSVGSGESYEQYF	TCRBV29-01	TCRBJ02-07
CSAHPEAFF	TCRBV20-01	TCRBJ01-01
CASSQGSDSGNTIYF	TCRBV03-01/03-02	TCRBJ01-03
CASSQNTGGQETQYF	TCRBV05-01	TCRBJ02-05
CASSVGASGSLLETQYF	TCRBV09-01	TCRBJ02-05
CASSEYQEATEAFF	TCRBV25-01	TCRBJ01-01
CASSLVMAGTEAFF	TCRBV05-01	TCRBJ01-01
CASSQGTGEGYEQYF	TCRBV04-01	TCRBJ02-07
CASSQGPYGYTF	TCRBV11-03	TCRBJ01-02
CASSLTGTGGRQPQHF	TCRBV07-02	TCRBJ01-05
CASSTGGENTIYF	TCRBV05-01	TCRBJ01-03
CSARNQGPGNTIYF	TCRBV20	TCRBJ01-03
CASSYLAGDTGELFF	TCRBV05-01	TCRBJ02-02
CATGTDLETQYF	TCRBV24-01	TCRBJ02-05
CASSQSLGETQYF	TCRBV07-02	TCRBJ02-05
CASSRGPLYEQYF	TCRBV07	TCRBJ02-07
CSAAGGTNTEAFF	TCRBV29-01	TCRBJ01-01
CASSFLGRDTQYF	TCRBV05-01	TCRBJ02-03
CASSRGPTYEQYF	TCRBV07	TCRBJ02-07
CSVDAGLQETQYF	TCRBV29-01	TCRBJ02-05
CASSPGTSGRASQETQYF	TCRBV05-01	TCRBJ02-05
CSVGSGLGYEQYF	TCRBV29-01	TCRBJ02-07
CASSVGWGELFF	TCRBV09-01	TCRBJ02-02
CASSLAGYSNQPQHF	TCRBV05-08	TCRBJ01-05

CASSLGGIPYGYTF	TCRBV27-01	TCRBJ01-02
CASSSLNTEAFF	TCRBV27-01	TCRBJ01-01
CASSLEGRASYEQYF	TCRBV11-02	TCRBJ02-07
CASSMGPNYEQYF	TCRBV07	TCRBJ02-07
CASSRLVNYGYTF	TCRBV07-02	TCRBJ01-02
CSARDREVTGELFF	TCRBV20	TCRBJ02-02
CASSLAWGEQYF	TCRBV07-02	TCRBJ02-07
CSARDRGSPNTGELFF	TCRBV20	TCRBJ02-02
CASSARNSGNTIYF	TCRBV28-01	TCRBJ01-03
CASSTTVNYGYTF	TCRBV07-02	TCRBJ01-02
CSARDLGTENTGELFF	TCRBV20	TCRBJ02-02
CASSLGGLGYGYTF	TCRBV27-01	TCRBJ01-02
CASSLNTGTQETQYF	TCRBV05-01	TCRBJ02-05
CSGAEGTNTEAFF	TCRBV29-01	TCRBJ01-01
CASSLGLNYEQYF	TCRBV07-06	TCRBJ02-07
CASSSPRGGENSPLHF	TCRBV06-05	TCRBJ01-06
CASSLDGQAYEQYF	TCRBV07-02	TCRBJ02-07
CSARDRGTKNTGELFF	TCRBV20	TCRBJ02-02
CASSGDTQYF	TCRBV07	TCRBJ02-03
CASSLDWGEQYF	TCRBV07-02	TCRBJ02-07
CASSLSNGETQYF	TCRBV07-02	TCRBJ02-05
CASSSGPRYEQYF	TCRBV07	TCRBJ02-07
CASSLGGINYGYTF	TCRBV27-01	TCRBJ01-02
CASSPGPGYEQYF	TCRBV07-06	TCRBJ02-07
CASSPIGGLTDTQYF	TCRBV03-01/03-02	TCRBJ02-03
CASSFSGQNTEAFF	TCRBV12	TCRBJ01-01
CASSLNLGGQETQYF	TCRBV05-01	TCRBJ02-05
CASSLQGYSNQPQHF	TCRBV05-08	TCRBJ01-05
CASSPGPNYEQYF	TCRBV07-06	TCRBJ02-07
CASSYQGYSNQPQHF	TCRBV05-08	TCRBJ01-05
CSARAQGRSYEQYF	TCRBV20	TCRBJ02-07
CASSRGGPSYEQYF	TCRBV14-01	TCRBJ02-07
CSVEGGRLTDTQYF	TCRBV29-01	TCRBJ02-03
CASSSLHTEAFF	TCRBV27-01	TCRBJ01-01
CASSSGPAYEQYF	TCRBV07	TCRBJ02-07
CASSFLAGRTGELFF	TCRBV05-01	TCRBJ02-02
CASSLRGPSTDTQYF	TCRBV18-01	TCRBJ02-03
CASSPQKGPGEQYF	TCRBV18-01	TCRBJ02-07
CASSIAGLLSSYNEQFF	TCRBV19-01	TCRBJ02-01
CASSPGGLPYGYTF	TCRBV27-01	TCRBJ01-02
CSAPPRGDSPLHF	TCRBV20	TCRBJ01-06

CASRGRGNNEQFF	TCRBV19-01	TCRBJ02-01
CASRGRGDNEQFF	TCRBV19-01	TCRBJ02-01
CASSLGGQAYGYTF	TCRBV27-01	TCRBJ01-02
CASSQDGGSSTDTQYF	TCRBV04-03	TCRBJ02-03
CASSKSWGEQYF	TCRBV07-02	TCRBJ02-07
CASSLLLAGPYEQYF	TCRBV11-02	TCRBJ02-07
CASSQGPNYEQYF	TCRBV07-06	TCRBJ02-07
CASSGLNNEQFF	TCRBV03-01/03-02	TCRBJ02-01
CSARGSSGSRETQYF	TCRBV20	TCRBJ02-05
CSARDRGTGNTGELFF	TCRBV20	TCRBJ02-02
CASSPRLAGTDTQYF	TCRBV12	TCRBJ02-03
CASSLNTGGGETQYF	TCRBV05-01	TCRBJ02-05
CASSFGGQPYGYTF	TCRBV27-01	TCRBJ01-02
CASSLVNRYEQYF	TCRBV07-02	TCRBJ02-07
CASSYSKQGGYEQYF	TCRBV06-05	TCRBJ02-07
CSVGGGEGYEQYF	TCRBV29-01	TCRBJ02-07
CASSRGPNYEQYV	TCRBV07-06	TCRBJ02-07
CASSHVDRNYGYTF	TCRBV04-01	TCRBJ01-02
CASSLPWGEQYF	TCRBV07-02	TCRBJ02-07
CSARGSSGGRETQYF	TCRBV20	TCRBJ02-05
CASSVGQSRSYNSPLHF	TCRBV09-01	TCRBJ01-06
CASSVGASGSVGETQYF	TCRBV09-01	TCRBJ02-05
CASSLLLAGTYNEQFF	TCRBV07-09	TCRBJ02-01
CASSYGGQVYGYTF	TCRBV06-05	TCRBJ01-02
CASSSGPVYEQYF	TCRBV07-06	TCRBJ02-07
CSVAKGSEAFF	TCRBV29-01	TCRBJ01-01
CASSFPLGTDTQYF	TCRBV28-01	TCRBJ02-03
CASSLGGYDYGYTF	TCRBV27-01	TCRBJ01-02
CASSLGSSHQPQHF	TCRBV07-02	TCRBJ01-05
CASSVGEGRSYNSPLHF	TCRBV09-01	TCRBJ01-06
CAWSWAGEQYF	TCRBV30-01	TCRBJ02-07
CSARAGSRSYEQYF	TCRBV20	TCRBJ02-07
CASSAGPNYEQYF	TCRBV07-06	TCRBJ02-07
CASSPRLSNQPQHF	TCRBV11-02	TCRBJ01-05
CASSYGRQPYGYTF	TCRBV06-05	TCRBJ01-02
CSVEGTRGTDTQYF	TCRBV29-01	TCRBJ02-03
CASSRSGQNTEAFF	TCRBV12	TCRBJ01-01
CASSLSRGGADGYTF	TCRBV27-01	TCRBJ01-02
CSTRTDTQYF	TCRBV29-01	TCRBJ02-03
CASSPAGQGSPLHF	TCRBV18-01	TCRBJ01-06
CASSPGRNYEQYF	TCRBV07-06	TCRBJ02-07

CASSFPGQNTEAFF	TCRBV12	TCRBJ01-01
CASSLWLAGTYNEQFF	TCRBV07-09	TCRBJ02-01
CASSRGPNYEQYF	TCRBV07-06	TCRBJ02-07
CAWTWGGEQYF	TCRBV30-01	TCRBJ02-07
CASSWGTSGRASKETQYF	TCRBV05-01	TCRBJ02-05
CASSLEANTDTQYF	TCRBV05-01	TCRBJ02-03
CSGGSGEGYEQYF	TCRBV29-01	TCRBJ02-07
CASSLEAITDTQYF	TCRBV05-01	TCRBJ02-03
CASSLYLAGTYNEQFF	TCRBV07-09	TCRBJ02-01
CASSQGRNYEQYF	TCRBV07	TCRBJ02-07
CASSATGVSGNTIYF	TCRBV11-03	TCRBJ01-03
CAWRDGFNTEAFF	TCRBV30-01	TCRBJ01-01
CASSLVTGGRETQYF	TCRBV05-06	TCRBJ02-05
CASSLEGRGFYEQYF	TCRBV11-02	TCRBJ02-07
CSVGSGGGYEQYF	TCRBV29-01	TCRBJ02-07
CASQLTSSTDTQYF	TCRBV11-03	TCRBJ02-03
CASSSGPLYEQYF	TCRBV07	TCRBJ02-07
CASSPRGPGTDTQYF	TCRBV18-01	TCRBJ02-03
CSPRGLYNEQFF	TCRBV20	TCRBJ02-01
CASSTGGLPYGYTF	TCRBV27-01	TCRBJ01-02
CASSFDGRDTQYF	TCRBV05-01	TCRBJ02-03
CASSPQLAGGDTQYF	TCRBV12	TCRBJ02-03
CASSHPDRNYGYTF	TCRBV04-01	TCRBJ01-02
CAWSWVGEQYF	TCRBV30-01	TCRBJ02-07
CASSVGQAYEQYF	TCRBV07-08	TCRBJ02-07
CASSQGHRSGNTIYF	TCRBV03-01/03-02	TCRBJ01-03
CASSVGAGRSYNSPLHF	TCRBV09-01	TCRBJ01-06
CASSLDSTSGNTIYF	TCRBV05-05	TCRBJ01-03
CASSTGPNYEQYF	TCRBV07	TCRBJ02-07
CASSPRSPSTDTQYF	TCRBV18-01	TCRBJ02-03
CASSSGPGYEQYF	TCRBV07-06	TCRBJ02-07
CSARRDGPGNTIYF	TCRBV20	TCRBJ01-03
CSARWRDYGYTF	TCRBV20	TCRBJ01-02
CASSVGGAANTEAFF	TCRBV09-01	TCRBJ01-01
CASSPRGGTTDTQYF	TCRBV03-01/03-02	TCRBJ02-03
CASSIGGENTIYF	TCRBV05-01	TCRBJ01-03
CASSVRLGELFF	TCRBV09-01	TCRBJ02-02
CASSIGGQPYGYTF	TCRBV06-05	TCRBJ01-02
CASSPGPDYEQYF	TCRBV07-06	TCRBJ02-07
CASSGGAVSTDTQYF	TCRBV02-01	TCRBJ02-03
CASSLILGETQYF	TCRBV07-02	TCRBJ02-05

CASSLGPNYEQYF	TCRBV07-06	TCRBJ02-07
CSAAQGTNTEAFF	TCRBV29-01	TCRBJ01-01
CASSSRGGREKLFF	TCRBV05-01	TCRBJ01-04
CASSPKGQGSGYTF	TCRBV18-01	TCRBJ01-02
CASVFQETQYF	TCRBV02-01	TCRBJ02-05
CASSQDLAGASDTQYF	TCRBV04-03	TCRBJ02-03
CASSSGTNYEQYF	TCRBV07	TCRBJ02-07
CASSAGQAYEQYF	TCRBV07-08	TCRBJ02-07
CASSPESQGSPLHF	TCRBV18-01	TCRBJ01-06
CASSLEGENTIYF	TCRBV05-01	TCRBJ01-03
CASSLALAGTYNEQFF	TCRBV07-09	TCRBJ02-01
CSAFPEAFF	TCRBV20-01	TCRBJ01-01
CASSLLVGAYEQYF	TCRBV07-02	TCRBJ02-07
CASSRTRNYGYTF	TCRBV07-02	TCRBJ01-02
CSARDPGTENTGELFF	TCRBV20	TCRBJ02-02
CASSYSGQNTEAFF	TCRBV12	TCRBJ01-01
CASSSGPNYEQYF	TCRBV07-06	TCRBJ02-07
CASSQGNYGYTF	TCRBV11-03	TCRBJ01-02
CASSEYQEGTEAFF	TCRBV25-01	TCRBJ01-01
CASSNLAGDTGELFF	TCRBV05-01	TCRBJ02-02
CSARAGGRNYEQYF	TCRBV20	TCRBJ02-07
CASSLFGGRSYEQYF	TCRBV05-01	TCRBJ02-07
CASSPGTGKGYEQYF	TCRBV04-01	TCRBJ02-07
CASSLTLAGGPNEQFF	TCRBV05-01	TCRBJ02-01
CASSYNTGGQETQYF	TCRBV05-01	TCRBJ02-05
CASSLGGEPYGYTF	TCRBV27-01	TCRBJ01-02
CATRFAGELFF	TCRBV27-01	TCRBJ02-02
CSVGSGEDGEQFF	TCRBV29-01	TCRBJ02-01
CASSVETGAGGELFF	TCRBV09-01	TCRBJ02-02
CSVARGSQAFF	TCRBV29-01	TCRBJ01-01
CASSQEGGISTDTQYF	TCRBV04-03	TCRBJ02-03
CASSLGGQPYGYTF	TCRBV27-01	TCRBJ01-02
CASSKTSSTDTQYF	TCRBV11-03	TCRBJ02-03
CASSLASSNEQFF	TCRBV28-01	TCRBJ02-01
CASSPGQGESYEQYF	TCRBV04-01	TCRBJ02-07
CSVEGGWGTDTQYF	TCRBV29-01	TCRBJ02-03
CASSLSRGAADGYTF	TCRBV27-01	TCRBJ01-02
CASSRTNNYGYTF	TCRBV07-02	TCRBJ01-02
CASSPEGQGTPLHF	TCRBV18-01	TCRBJ01-06
CSANRGEGYTF	TCRBV20-01	TCRBJ01-02
CASSLVHPYEQYF	TCRBV07-02	TCRBJ02-07

CASSFWGRDTQYF	TCRBV05-01	TCRBJ02-03
CSANNRDYGYTF	TCRBV20	TCRBJ01-02
CASSLAPNYEQYF	TCRBV07-06	TCRBJ02-07
CASSHGGLPYGYTF	TCRBV27-01	TCRBJ01-02
CASSSGQNYEQYF	TCRBV07	TCRBJ02-07
CASSLVGERSYEQYF	TCRBV05-01	TCRBJ02-07
CASSLGGMPYGYTF	TCRBV27-01	TCRBJ01-02
CASSHVNNEQFF	TCRBV03-01/03-02	TCRBJ02-01
CSAADGTNTEAFF	TCRBV29-01	TCRBJ01-01
CASSFSGWNTEAFF	TCRBV12	TCRBJ01-01
CASSLTGRGTDTQYF	TCRBV28-01	TCRBJ02-03
CASSFVTGGTEAFF	TCRBV07-02	TCRBJ01-01
CSARDRGTANTGELFF	TCRBV20	TCRBJ02-02
CASSLITGGQETQYF	TCRBV05-01	TCRBJ02-05
CASSSGPAYEQYF	TCRBV07-06	TCRBJ02-07
CASSYGGLPYGYTF	TCRBV06-05	TCRBJ01-02
CASSVDRDSPLHF	TCRBV10-02	TCRBJ01-06
CSVDAGYQETQYF	TCRBV29-01	TCRBJ02-05
CASSFKGGNIQYF	TCRBV07-02	TCRBJ02-04
CASSLGGVPYGYTF	TCRBV27-01	TCRBJ01-02
CASSEGQAYEQYF	TCRBV07-08	TCRBJ02-07
CAWSWGAEQYF	TCRBV30-01	TCRBJ02-07
CASSLGNNYEQYF	TCRBV07-06	TCRBJ02-07
CASSVDTGATGELFF	TCRBV09-01	TCRBJ02-02
CASSLWGRDTQYF	TCRBV05-01	TCRBJ02-03
CASSRGGLFYEQYF	TCRBV14-01	TCRBJ02-07
CASSDGQGEGYEQYF	TCRBV04-01	TCRBJ02-07
CASSVGQDRSYNSPLHF	TCRBV09-01	TCRBJ01-06
CSVGTGEGYEQYF	TCRBV29-01	TCRBJ02-07
CSVDRGFQETQYF	TCRBV29-01	TCRBJ02-05
CASSFEGQNIQYF	TCRBV07-02	TCRBJ02-04
CASSFPRGGENSPLHF	TCRBV06-05	TCRBJ01-06
CSARDRVVTGELFF	TCRBV20	TCRBJ02-02
CASSQGPDYEQYF	TCRBV07	TCRBJ02-07
CSAQAGGRSYEQYF	TCRBV20	TCRBJ02-07
CASSVGGLPYGYTF	TCRBV27-01	TCRBJ01-02
CASSLTANTDTQYF	TCRBV05-01	TCRBJ02-03
CASSGGAGLTDTQYF	TCRBV02-01	TCRBJ02-03
CASSGRLAGPYEQYF	TCRBV11-02	TCRBJ02-07
CASSPEGSGSPLHF	TCRBV18-01	TCRBJ01-06
CASSLYLAGAYNEQFF	TCRBV07-09	TCRBJ02-01

CASSLEGRPSYEQYF	TCRBV11-02	TCRBJ02-07
CSAYNRDYGYTF	TCRBV20	TCRBJ01-02
CASSPGENYEQYF	TCRBV07-06	TCRBJ02-07
CASSSSLGETQYF	TCRBV07-02	TCRBJ02-05
CASARLANTGELFF	TCRBV28-01	TCRBJ02-02
CSAATGTNTEAFF	TCRBV29-01	TCRBJ01-01
CASSVETGFTGELFF	TCRBV09-01	TCRBJ02-02
CSARNREYGYTF	TCRBV20	TCRBJ01-02
CASSLQAYSNQPQHF	TCRBV05-08	TCRBJ01-05
CASSMGGQPYGYTF	TCRBV27-01	TCRBJ01-02
CASSQESRSGNTIYF	TCRBV03-01/03-02	TCRBJ01-03
CASSNLNSPLHF	TCRBV27-01	TCRBJ01-06
CASSQGGNYEQYF	TCRBV07-06	TCRBJ02-07
CASSQDLAGATDTQYF	TCRBV04-03	TCRBJ02-03
CASSPPGQGVGQPQHF	TCRBV12	TCRBJ01-05
CASSLGGLSYGYTF	TCRBV27-01	TCRBJ01-02
CASSPGMGEGYEQYF	TCRBV04-01	TCRBJ02-07
CASSVWGRDTQYF	TCRBV05-01	TCRBJ02-03
CASSLEALTDTQYF	TCRBV05-01	TCRBJ02-03
CASSSRNSGNTIYF	TCRBV28-01	TCRBJ01-03
CASSLIDRDTEAFF	TCRBV19-01	TCRBJ01-01
CASSLGGLPYGYTF	TCRBV27-01	TCRBJ01-02
CASSSGPIYEQYF	TCRBV07	TCRBJ02-07
CASSSGQNYEQYF	TCRBV07-06	TCRBJ02-07
CASSRGPSYEQYF	TCRBV07-06	TCRBJ02-07
CASSLVAGGRETQYF	TCRBV05-06	TCRBJ02-05
CASSPWGRGTDTQYF	TCRBV28-01	TCRBJ02-03
CASSRTVSYGYTF	TCRBV07-02	TCRBJ01-02
CASSYGGMPYGYTF	TCRBV06-05	TCRBJ01-02
CASSLSLAGSYNEQFF	TCRBV07-09	TCRBJ02-01
CASSVGQGRSYNSPLHF	TCRBV09-01	TCRBJ01-06
CASSFRGRDTQYF	TCRBV05-01	TCRBJ02-03
CSGDTDTQYF	TCRBV29-01	TCRBJ02-03
CASSVETGTTGELFF	TCRBV09-01	TCRBJ02-02
CASSTNTGGQETQYF	TCRBV05-01	TCRBJ02-05
CASSLSSGGADGYTF	TCRBV27-01	TCRBJ01-02
CASSHGSRSGNTIYF	TCRBV03-01/03-02	TCRBJ01-03
CSARAFGRSYEQYF	TCRBV20	TCRBJ02-07
CASSPKGDQPQHF	TCRBV07-02	TCRBJ01-05
CSVARGQEAFF	TCRBV29-01	TCRBJ01-01
CSARDRGGENTGELFF	TCRBV20	TCRBJ02-02

CASSVSLAGAYNEQFF	TCRBV07-09	TCRBJ02-01
CSARQQGPGNTIYF	TCRBV20	TCRBJ01-03
CASSVETGATGELFF	TCRBV09-01	TCRBJ02-02
CASSSGGNYEQYF	TCRBV07-06	TCRBJ02-07
CASSSRAGGEKLFF	TCRBV05-01	TCRBJ01-04
CSVEGPRGTDTQYF	TCRBV29-01	TCRBJ02-03
CASSPGPNYEQYF	TCRBV07	TCRBJ02-07
CASSHLPNEQFF	TCRBV03-01/03-02	TCRBJ02-01
CASSQDTQYF	TCRBV07	TCRBJ02-03
CASSLTVSTDTQYF	TCRBV11-03	TCRBJ02-03
CSVGQGEGYEQYF	TCRBV29-01	TCRBJ02-07
CASSQGSRSGNTIYF	TCRBV03-01/03-02	TCRBJ01-03
CASSQSRGSGNTIYF	TCRBV04-01	TCRBJ01-03
CSTRGLYNEQFF	TCRBV20	TCRBJ02-01
CSARESSGGGNEQFF	TCRBV20	TCRBJ02-01
CASSQDVQYGYTF	TCRBV04-03	TCRBJ01-02
CASSGDRDSPLHF	TCRBV10-02	TCRBJ01-06
CSARDRGTTNTGELFF	TCRBV20	TCRBJ02-02
CASSRGLNYEQYF	TCRBV07-06	TCRBJ02-07
CASSSENTEAFF	TCRBV27-01	TCRBJ01-01
CSAAAGTNTEAFF	TCRBV29-01	TCRBJ01-01
CASSQGSHSGNTIYF	TCRBV03-01/03-02	TCRBJ01-03
CSVGQDSTDTQYF	TCRBV29-01	TCRBJ02-03
CASSLGRDPTGELFF	TCRBV28-01	TCRBJ02-02
CASSVGGMVNTEAFF	TCRBV09-01	TCRBJ01-01
CASSPAGQGSGYTF	TCRBV18-01	TCRBJ01-02
CASLFQETQYF	TCRBV02-01	TCRBJ02-05
CASSHTDRNYGYTF	TCRBV04-01	TCRBJ01-02
CASSQGPGYEQYF	TCRBV07	TCRBJ02-07
CASSTSRGAGNTIYF	TCRBV04-01	TCRBJ01-03
CSARDRGAENTGELFF	TCRBV20	TCRBJ02-02
CASSLGGYGYGYTF	TCRBV27-01	TCRBJ01-02
CASSYARGGENSPLHF	TCRBV06-05	TCRBJ01-06
CASSLALAGMYNEQFF	TCRBV07-09	TCRBJ02-01
CASSEGPNYEQYF	TCRBV07-06	TCRBJ02-07
CASSLVGGRDYEQYF	TCRBV05-01	TCRBJ02-07
CASSLSLGETQYF	TCRBV07-02	TCRBJ02-05
CSAAEGMNTEAFF	TCRBV29-01	TCRBJ01-01
CSARDRGTPNTGELFF	TCRBV20	TCRBJ02-02
CSVGSGTGYEQYF	TCRBV29-01	TCRBJ02-07
CSARDRGSENTGELFF	TCRBV20	TCRBJ02-02

CASSLEGRGAYEQYF	TCRBV11-02	TCRBJ02-07
CASSVGASGSLGETQYF	TCRBV09-01	TCRBJ02-05
CSALDGDTEAFF	TCRBV20	TCRBJ01-01
CASSFGGLPYGYTF	TCRBV27-01	TCRBJ01-02
CSVGPGEGYEQYF	TCRBV29-01	TCRBJ02-07
CASSLDSRSGNTIYF	TCRBV05-05	TCRBJ01-03
CASSPRGGRTDTQYF	TCRBV03-01/03-02	TCRBJ02-03
CASSVTGAVNTEAFF	TCRBV09-01	TCRBJ01-01
CASSVGLGELFF	TCRBV09-01	TCRBJ02-02
CASGDLNSPLHF	TCRBV27-01	TCRBJ01-06
CASSPQGGPNEQYF	TCRBV18-01	TCRBJ02-07
CASSPGPPYEQYF	TCRBV07	TCRBJ02-07
CASSRGQNYEQYF	TCRBV07-06	TCRBJ02-07
CASSRGPTYEQYF	TCRBV07-06	TCRBJ02-07
CASSLGGSPYGYTF	TCRBV27-01	TCRBJ01-02
CASSLVGGRSYEQYF	TCRBV05-01	TCRBJ02-07
CASSRGGLPYGYTF	TCRBV27-01	TCRBJ01-02
CASSVKTGGTGELFF	TCRBV09-01	TCRBJ02-02
CASSPTGPQETQYF	TCRBV07-03	TCRBJ02-05
CSGEGGRGTDTQYF	TCRBV29-01	TCRBJ02-03
CASSIIERDTEAFF	TCRBV19-01	TCRBJ01-01
CASSLSRGGASGYTF	TCRBV27-01	TCRBJ01-02
CASSRRGPSTDTQYF	TCRBV18-01	TCRBJ02-03
CASVSLNTEAFF	TCRBV27-01	TCRBJ01-01
CASSPEGPSTDTQYF	TCRBV18-01	TCRBJ02-03
CASSIQGYSNQPQHF	TCRBV05-08	TCRBJ01-05
CASSRDSGSGNTIYF	TCRBV05-05	TCRBJ01-03
CSARDRETTGELFF	TCRBV20	TCRBJ02-02
CASSLDANTDTQYF	TCRBV05-01	TCRBJ02-03
CASSFEGANIQYF	TCRBV07-02	TCRBJ02-04
CASSSGPSYEQYF	TCRBV07-06	TCRBJ02-07
CASSLKTGGQETQYF	TCRBV05-01	TCRBJ02-05
CASSYGRGGENSPLHF	TCRBV06-05	TCRBJ01-06
CAWSMGGEQYF	TCRBV30-01	TCRBJ02-07
CASSPTGLQETQYF	TCRBV07-03	TCRBJ02-05
CSVAEGTNTEAFF	TCRBV29-01	TCRBJ01-01
CASSLQGENTIYF	TCRBV05-01	TCRBJ01-03
CASSNGGQPYGYTF	TCRBV06-05	TCRBJ01-02
CASSVFTGGTGELFF	TCRBV09-01	TCRBJ02-02
CASSSRGMGEKLFF	TCRBV05-01	TCRBJ01-04
CSVEGHRGTDTQYF	TCRBV29-01	TCRBJ02-03

CASSIGPNYEQYF	TCRBV07	TCRBJ02-07
CASSSGQLYEQYF	TCRBV07-08	TCRBJ02-07
CASSSSNSGNTIYF	TCRBV28-01	TCRBJ01-03
CASSQGPSYEQYF	TCRBV07	TCRBJ02-07
CASSVDTGGTGELFF	TCRBV09-01	TCRBJ02-02
CASSLTWGEQYF	TCRBV07-02	TCRBJ02-07
CASSLGGGPYGYTF	TCRBV27-01	TCRBJ01-02
CSVAQGSEAFF	TCRBV29-01	TCRBJ01-01
CSSNTDTQYF	TCRBV29-01	TCRBJ02-03
CASSVQGAVNTEAFF	TCRBV09-01	TCRBJ01-01
CASSGQGYSNQPQHF	TCRBV05-08	TCRBJ01-05
CASSRGPAYEQYF	TCRBV07	TCRBJ02-07
CSTGTDTQYF	TCRBV29-01	TCRBJ02-03
CASSPEGQGSGYTF	TCRBV18-01	TCRBJ01-02
CASSYGGQFYGYTF	TCRBV06-05	TCRBJ01-02
CASSLGGENTIYF	TCRBV05-01	TCRBJ01-03
CASSFLAGDTGELFF	TCRBV05-01	TCRBJ02-02
CASSRPVNYGYTF	TCRBV07-02	TCRBJ01-02
CASSLWGRGTDTQYF	TCRBV28-01	TCRBJ02-03
CASSVGGAVNTEAFF	TCRBV09-01	TCRBJ01-01
CASSSTSSTDTQYF	TCRBV11-03	TCRBJ02-03
CAWGTGFNTEAFF	TCRBV30-01	TCRBJ01-01
CASSGGAGITDTQYF	TCRBV02-01	TCRBJ02-03
CASSSGPTYEQYF	TCRBV07-06	TCRBJ02-07
CASSPRLAGGDTQYF	TCRBV12	TCRBJ02-03
CASSLSLAGNYNEQFF	TCRBV07-09	TCRBJ02-01
CASSLEVRGSYEQYF	TCRBV11-02	TCRBJ02-07
CASSGTGLQETQYF	TCRBV07-03	TCRBJ02-05
CASSLGPNYEQYF	TCRBV07	TCRBJ02-07
CASSINTGGQETQYF	TCRBV05-01	TCRBJ02-05
CSARAGGKSYEQYF	TCRBV20	TCRBJ02-07
CAWITGFNTEAFF	TCRBV30-01	TCRBJ01-01
CSVGSGEHYEQYF	TCRBV29-01	TCRBJ02-07
CASSAGQGEQPQHF	TCRBV02-01	TCRBJ01-05
CASSVETRGTGELFF	TCRBV09-01	TCRBJ02-02
CASSPEGQGSPLHF	TCRBV18-01	TCRBJ01-06
CASSQTGVSGNTIYF	TCRBV11-03	TCRBJ01-03
CASSLVWGGRETQYF	TCRBV05-06	TCRBJ02-05
CASSVVGAVNTEAFF	TCRBV09-01	TCRBJ01-01
CASSYSPAGGYEQYF	TCRBV06-05	TCRBJ02-07
CASSSGQGEGYEQYF	TCRBV04-01	TCRBJ02-07

CASSFGLGTDTQYF	TCRBV28-01	TCRBJ02-03
CASSFDGDQPQHF	TCRBV07-02	TCRBJ01-05
CASSSRGEGEKLFF	TCRBV05-01	TCRBJ01-04
CASSQGTRSGNTIYF	TCRBV03-01/03-02	TCRBJ01-03
CSVGSGERYEQYF	TCRBV29-01	TCRBJ02-07
CASSLGGAPYGYTF	TCRBV27-01	TCRBJ01-02
CASSRSLGETQYF	TCRBV07-02	TCRBJ02-05
CAWRTGINTEAFF	TCRBV30-01	TCRBJ01-01
CSAGGGTGKNIQYF	TCRBV20	TCRBJ02-04
CASSLRGGRSYEQYF	TCRBV05-01	TCRBJ02-07
CASSLSLAAAYNEQFF	TCRBV07-09	TCRBJ02-01
CASSEGRGEQPQHF	TCRBV02-01	TCRBJ01-05
CASSPGQGEFYEQYF	TCRBV04-01	TCRBJ02-07
CASSPFGQGSGYTF	TCRBV18-01	TCRBJ01-02
CASSLVASGRETQYF	TCRBV05-06	TCRBJ02-05
CASSKTVNYGYTF	TCRBV07-02	TCRBJ01-02
CASSSRGYGEKLFF	TCRBV05-01	TCRBJ01-04
CASSVGQGRTYNSPLHF	TCRBV09-01	TCRBJ01-06
CASSLEGHGSYEQYF	TCRBV11-02	TCRBJ02-07
CASSLSWGEQYF	TCRBV07-02	TCRBJ02-07
CASSLTLAGGRNEQFF	TCRBV05-01	TCRBJ02-01
CSSRGSYNEQFF	TCRBV20	TCRBJ02-01
CASSYPRGGEDSPLHF	TCRBV06-05	TCRBJ01-06
CASSFGGRSSGANVLTF	TCRBV05-04	TCRBJ02-06
CASSGVAGSTDTQYF	TCRBV02-01	TCRBJ02-03
CASSLSFGEQYF	TCRBV07-02	TCRBJ02-07
CASSRETGGTGELFF	TCRBV09-01	TCRBJ02-02
CASSPGQGEGYEQYV	TCRBV04-01	TCRBJ02-07
CASSHSDRNYGYTF	TCRBV04-01	TCRBJ01-02
CASSRSWGEQYF	TCRBV07-02	TCRBJ02-07
CSVGSGQGYEQYF	TCRBV29-01	TCRBJ02-07
CASSPGPFYEQYF	TCRBV07-06	TCRBJ02-07
CASSQASRSGNTIYF	TCRBV03-01/03-02	TCRBJ01-03
CSSRGVYNEQFF	TCRBV20	TCRBJ02-01
CSTYTDTQYF	TCRBV29-01	TCRBJ02-03
CASSLNTGGMETQYF	TCRBV05-01	TCRBJ02-05
CASSLLQGAHEQYF	TCRBV07-02	TCRBJ02-07
CASSFKGDQPQHF	TCRBV07-02	TCRBJ01-05
CASRGRGYNEQFF	TCRBV19-01	TCRBJ02-01
CASSKGGLPYGYTF	TCRBV27-01	TCRBJ01-02
CASSLEAGGRETQYF	TCRBV05-06	TCRBJ02-05

CASSSGQRYEQYF	TCRBV07-08	TCRBJ02-07
CASSLEGRGIYEQYF	TCRBV11-02	TCRBJ02-07
CASSLVGGRAYEQYF	TCRBV05-01	TCRBJ02-07
CASSVEIGGTGELFF	TCRBV09-01	TCRBJ02-02
CASSQGPAYEQYF	TCRBV07	TCRBJ02-07
CASSVNTGGTGELFF	TCRBV09-01	TCRBJ02-02
CASSPEGQGSSYTF	TCRBV18-01	TCRBJ01-02
CASSLGPEYEQYF	TCRBV07-06	TCRBJ02-07
CIVDTDTQYF	TCRBV29-01	TCRBJ02-03
CSVGSDSTDTQYF	TCRBV29-01	TCRBJ02-03
CASRFLAGDTGELFF	TCRBV05-01	TCRBJ02-02
CSVGPQSTDTQYF	TCRBV29-01	TCRBJ02-03
CASSQGQGEGYEQYF	TCRBV04-01	TCRBJ02-07
CASSLGQAYEQYV	TCRBV07-08	TCRBJ02-07
CSVETGRGTDTQYF	TCRBV29-01	TCRBJ02-03
CASSRGPNYEQYV	TCRBV07	TCRBJ02-07
CSSRGDYNEQFF	TCRBV20	TCRBJ02-01
CASSPRYSNQPQHF	TCRBV11-02	TCRBJ01-05
CSARDREYTGELFF	TCRBV20	TCRBJ02-02
CASSRGPNYEQYF	TCRBV07	TCRBJ02-07
CSTSTDTQYF	TCRBV29-01	TCRBJ02-03
CASTRTVNYGYTF	TCRBV07-02	TCRBJ01-02
CASSSGPSYEQYF	TCRBV07	TCRBJ02-07
CASSKNTGGQETQYF	TCRBV05-01	TCRBJ02-05
CASSLVNFYEQYF	TCRBV07-02	TCRBJ02-07
CASSLGGYNYGYTF	TCRBV27-01	TCRBJ01-02
CSAGSGEGYEQYF	TCRBV29-01	TCRBJ02-07
CASSLDSGSGNTIYF	TCRBV05-05	TCRBJ01-03
CAWGWGGEQYF	TCRBV30-01	TCRBJ02-07
CASSLGGFPYGYTF	TCRBV27-01	TCRBJ01-02
CASSLVAADTEAFF	TCRBV05-01	TCRBJ01-01
CASSLMLGETQYF	TCRBV07-02	TCRBJ02-05
CASSAPGQGVGQPQHF	TCRBV12	TCRBJ01-05
CASSPPGTGVGQPQHF	TCRBV12	TCRBJ01-05
CASSLALAGFYNEQFF	TCRBV07-09	TCRBJ02-01
CASSPNGGGTEAFF	TCRBV07-03	TCRBJ01-01
CSVITDTQYF	TCRBV29-01	TCRBJ02-03
CASSQGPSYEQYF	TCRBV07-06	TCRBJ02-07
CASSAGPNYEQYF	TCRBV07	TCRBJ02-07
CASSPGTGRGYEQYF	TCRBV04-01	TCRBJ02-07
CASSKGPNYEQYF	TCRBV07-06	TCRBJ02-07

CASSEGQGEQPQHF	TCRBV02-01	TCRBJ01-05
CASSQGPAYEQYF	TCRBV07-06	TCRBJ02-07
CASSDQGEDQPQHF	TCRBV10-02	TCRBJ01-05
CASSLGNNYEQYF	TCRBV07	TCRBJ02-07
CASSPGLNYEQYF	TCRBV07-06	TCRBJ02-07
CASSLFGRGTDTQYF	TCRBV28-01	TCRBJ02-03
CASSLGTENTIYF	TCRBV05-01	TCRBJ01-03
CASSLWLAGAYNEQFF	TCRBV07-09	TCRBJ02-01
CASSLGGEDTIYF	TCRBV05-01	TCRBJ01-03
CASSLWSSNEQFF	TCRBV28-01	TCRBJ02-01
CASSPETGATGELFF	TCRBV09-01	TCRBJ02-02
CSVTPDSTDTQYF	TCRBV29-01	TCRBJ02-03
CASSSGSAYEQYF	TCRBV07-08	TCRBJ02-07
CASSLWGRSTDTQYF	TCRBV28-01	TCRBJ02-03
CASSLVKPYEQYF	TCRBV07-02	TCRBJ02-07
CASSLEGSGSYEQYF	TCRBV11-02	TCRBJ02-07
CSVDAGFQETQYF	TCRBV29-01	TCRBJ02-05
CASSWGQAYEQYF	TCRBV07-08	TCRBJ02-07
CASSDGTSGGASETQYF	TCRBV09-01	TCRBJ02-05
CASSLQLGETQYF	TCRBV07-02	TCRBJ02-05
CSARNLDYGYTF	TCRBV20	TCRBJ01-02
CASSYGGQPYGYTF	TCRBV27-01	TCRBJ01-02
CASSYVAGGRETQYF	TCRBV05-06	TCRBJ02-05
CASSLSMGEQYF	TCRBV07-02	TCRBJ02-07
CASSLFGQPYGYTF	TCRBV27-01	TCRBJ01-02
CASSYQGQPYGYTF	TCRBV06-05	TCRBJ01-02
CASSLGPNYEQYV	TCRBV07	TCRBJ02-07
CASSSGPYYEQYF	TCRBV07-06	TCRBJ02-07
CASSLLQGAEKLFF	TCRBV12	TCRBJ01-04
CASSFGGENTIYF	TCRBV05-01	TCRBJ01-03
CASSLNTGGAETQYF	TCRBV05-01	TCRBJ02-05
CASSMGPNYEQYF	TCRBV07-06	TCRBJ02-07
CSARDRGTLNTGELFF	TCRBV20	TCRBJ02-02
CASSRGPEYEQYF	TCRBV07	TCRBJ02-07
CASSVEKGGTGELFF	TCRBV09-01	TCRBJ02-02
CASSLPGRGTDTQYF	TCRBV28-01	TCRBJ02-03
CASSYGGSPYGYTF	TCRBV06-05	TCRBJ01-02
CASSHLNNEQFF	TCRBV03-01/03-02	TCRBJ02-01
CASSYGQAYEQYF	TCRBV07-08	TCRBJ02-07
CASSLSRGGANGYTF	TCRBV27-01	TCRBJ01-02
CASSLSLAGPYNEQFF	TCRBV07-09	TCRBJ02-01

CAWSWGEEQYF	TCRBV30-01	TCRBJ02-07
CASSARGGLTDTQYF	TCRBV03-01/03-02	TCRBJ02-03
CASSEAQGEQPQHF	TCRBV02-01	TCRBJ01-05
CASSYPGQPYGYTF	TCRBV06-05	TCRBJ01-02
CASSVGGAVGTEAFF	TCRBV09-01	TCRBJ01-01
CASSLSLAGALNEQFF	TCRBV07-09	TCRBJ02-01
CASSLALAGTNNEQFF	TCRBV07-09	TCRBJ02-01
CASSQEGGSSTDTQYF	TCRBV04-03	TCRBJ02-03
CSVEGGRGRDTQYF	TCRBV29-01	TCRBJ02-03
CASSFGQGEGYEQYF	TCRBV04-01	TCRBJ02-07
CASSLSPGQNTEAFF	TCRBV04-01	TCRBJ01-01
CSAGEGTNTEAFF	TCRBV29-01	TCRBJ01-01
CASSTQGYSNQPQHF	TCRBV05-08	TCRBJ01-05
CASSQGQRSGNTIYF	TCRBV03-01/03-02	TCRBJ01-03
CSARGSAGGRETQYF	TCRBV20	TCRBJ02-05
CASSLEARGSYEQYF	TCRBV11-02	TCRBJ02-07
CASSVGPNYEQYF	TCRBV07-06	TCRBJ02-07
CASSLVGGRVYEQYF	TCRBV05-01	TCRBJ02-07
CSARNFDYGYTF	TCRBV20	TCRBJ01-02
CSARDRGLENTGELFF	TCRBV20	TCRBJ02-02
CSVEGGRGVDTQYF	TCRBV29-01	TCRBJ02-03
CASSPEAQGSGYTF	TCRBV18-01	TCRBJ01-02
CASSQDLAGAQDTQYF	TCRBV04-03	TCRBJ02-03
CASSLWGTGTDTQYF	TCRBV28-01	TCRBJ02-03
CSVEYGRGTDTQYF	TCRBV29-01	TCRBJ02-03
CSVARGREAFF	TCRBV29-01	TCRBJ01-01
CASSEGQGLQPQHF	TCRBV02-01	TCRBJ01-05
CASSPRQGLTDTQYF	TCRBV03-01/03-02	TCRBJ02-03
CASSAGTGEGYEQYF	TCRBV04-01	TCRBJ02-07
CSVGSGSGYEQYF	TCRBV29-01	TCRBJ02-07
CSAKNRDYGYTF	TCRBV20	TCRBJ01-02
CASSHRGTGELFF	TCRBV28-01	TCRBJ02-02
CASGRLANTGELFF	TCRBV28-01	TCRBJ02-02
CASSHNNNEQFF	TCRBV03-01/03-02	TCRBJ02-01
CASKFLAGDTGELFF	TCRBV05-01	TCRBJ02-02
CASSLTSHTDTQYF	TCRBV11-03	TCRBJ02-03
CASSLNTGGQETQYF	TCRBV05-01	TCRBJ02-05
CASSLGGLAYGYTF	TCRBV27-01	TCRBJ01-02
CASSLSRGGAYGYTF	TCRBV27-01	TCRBJ01-02
CASSYGGQIYGYTF	TCRBV06-05	TCRBJ01-02
CASSPEGAGSGYTF	TCRBV18-01	TCRBJ01-02

CASSSGPEYEQYF	TCRBV07	TCRBJ02-07
CASSVGNAVNTEAFF	TCRBV09-01	TCRBJ01-01
CSAEEGTNTEAFF	TCRBV29-01	TCRBJ01-01
CASSLDGQVYEQYF	TCRBV07-02	TCRBJ02-07
CASSPGPFYEQYF	TCRBV07	TCRBJ02-07
CSAPRQGPGNTIYF	TCRBV20	TCRBJ01-03
CSVGLGEGYEQYF	TCRBV29-01	TCRBJ02-07
CSTFTDTQYF	TCRBV29-01	TCRBJ02-03
CASSIGGLPYGYTF	TCRBV27-01	TCRBJ01-02
CASSHLHNEQFF	TCRBV03-01/03-02	TCRBJ02-01
CSARAGGTSYEQYF	TCRBV20	TCRBJ02-07
CSVGSGAGYEQYF	TCRBV29-01	TCRBJ02-07
CASSLTSITDTQYF	TCRBV11-03	TCRBJ02-03
CASSSGPNYEQYF	TCRBV07	TCRBJ02-07
CSARDIGTENTGELFF	TCRBV20	TCRBJ02-02
CASSNGGLPYGYTF	TCRBV27-01	TCRBJ01-02
CASSSGPTYEQYF	TCRBV07	TCRBJ02-07
CASSPGPAYEQYF	TCRBV07-06	TCRBJ02-07
CASSSVSGGRGETQYF	TCRBV05-04	TCRBJ02-05
CASSFVQGGTEAFF	TCRBV07-02	TCRBJ01-01
CASSLPLAGETQYF	TCRBV11-02	TCRBJ02-05
CASSFDTQYF	TCRBV07	TCRBJ02-03
CASSSGGNYEQYF	TCRBV07	TCRBJ02-07
CAWSWGSEQYF	TCRBV30-01	TCRBJ02-07
CASRRTVNYGYTF	TCRBV07-02	TCRBJ01-02
CASSMTSGGRGETQYF	TCRBV05-04	TCRBJ02-05
CASSPGPNYEQYV	TCRBV07-06	TCRBJ02-07
CASSSGPHYEQYF	TCRBV07	TCRBJ02-07
CSVGWGEGYEQYF	TCRBV29-01	TCRBJ02-07
CASSPTGLVETQYF	TCRBV07-03	TCRBJ02-05
CSVEMGRGTDTQYF	TCRBV29-01	TCRBJ02-03
CASSFLNNEQFF	TCRBV03-01/03-02	TCRBJ02-01
CAWRTGFNTEAFF	TCRBV30-01	TCRBJ01-01
CATGGDLETQYF	TCRBV24-01	TCRBJ02-05
CASSQGPNYEQYF	TCRBV07	TCRBJ02-07
CASSPRGGLTDTQYF	TCRBV03-01/03-02	TCRBJ02-03
CASSLGPKYEQYF	TCRBV07	TCRBJ02-07
CASSPRGGGEKLFF	TCRBV05-01	TCRBJ01-04
CASSLLSGAYEQYF	TCRBV07-02	TCRBJ02-07
CASSPLGGPGEQYF	TCRBV18-01	TCRBJ02-07
CASSSGPNYEQYV	TCRBV07-06	TCRBJ02-07

CASSKGPNYEQYF	TCRBV07	TCRBJ02-07
CASSWGTSGRASQETQYF	TCRBV05-01	TCRBJ02-05
CASSSRGGGEKLFF	TCRBV05-01	TCRBJ01-04
CAWRWGGEQYF	TCRBV30-01	TCRBJ02-07
CSPGGGTGKNIQYF	TCRBV20	TCRBJ02-04
CSARDRRTENTGELFF	TCRBV20	TCRBJ02-02
CASSEGPNYEQYF	TCRBV07	TCRBJ02-07
CASSYGGPPYGYTF	TCRBV06-05	TCRBJ01-02
CSVGPDSTDTQYF	TCRBV29-01	TCRBJ02-03
CASSPRGQGSPLHF	TCRBV18-01	TCRBJ01-06
CASSLWQAYEQYF	TCRBV07-08	TCRBJ02-07
CSVEGGRYTDTQYF	TCRBV29-01	TCRBJ02-03
CASSNRGTGELFF	TCRBV28-01	TCRBJ02-02
CASSPNAGGTEAFF	TCRBV07-03	TCRBJ01-01
CASSFEGRGSYEQYF	TCRBV11-02	TCRBJ02-07
CASSTIDRDTEAFF	TCRBV19-01	TCRBJ01-01
CSASNRDYGYTF	TCRBV20	TCRBJ01-02
CASSVGGYPYGYTF	TCRBV27-01	TCRBJ01-02
CASSQDLAGITDTQYF	TCRBV04-03	TCRBJ02-03
CASSPRLAGVDTQYF	TCRBV12	TCRBJ02-03
CSENTDTQYF	TCRBV29-01	TCRBJ02-03
CASSQGDRSGNTIYF	TCRBV03-01/03-02	TCRBJ01-03
CASSWGQGERGYTF	TCRBV05-04	TCRBJ01-02
CASSSGQAYEQYF	TCRBV07-08	TCRBJ02-07
CSVADGSEAFF	TCRBV29-01	TCRBJ01-01
CSVDSGEGYEQYF	TCRBV29-01	TCRBJ02-07
CASSDSAGYGYTF	TCRBV02-01	TCRBJ01-02
CSVEGGRGTDTQYF	TCRBV29-01	TCRBJ02-03
CASSYGGQKYGYTF	TCRBV06-05	TCRBJ01-02
CASSPRVPSTDTQYF	TCRBV18-01	TCRBJ02-03
CASSFGGYPYGYTF	TCRBV27-01	TCRBJ01-02
CASRFAGELFF	TCRBV27-01	TCRBJ02-02
CASSHGGTGELFF	TCRBV28-01	TCRBJ02-02
CSVEAGRGTDTQYF	TCRBV29-01	TCRBJ02-03
CASSFMGQNTEAFF	TCRBV12	TCRBJ01-01
CASSPGPPYEQYF	TCRBV07-06	TCRBJ02-07
CSARIQGPGNTIYF	TCRBV20	TCRBJ01-03
CSARDRELTGELFF	TCRBV20	TCRBJ02-02
CASSVGRGRSYNSPLHF	TCRBV09-01	TCRBJ01-06
CASSPGQNYEQYF	TCRBV07-06	TCRBJ02-07
CASSTGQAYEQYF	TCRBV07-08	TCRBJ02-07

CASSLDSGAGNTIYF	TCRBV05-05	TCRBJ01-03
CASSVSRGSGNTIYF	TCRBV04-01	TCRBJ01-03
CASSPGHNYEQYF	TCRBV07-06	TCRBJ02-07
CASSVGASGSSGETQYF	TCRBV09-01	TCRBJ02-05
CASSLVAAGRETQYF	TCRBV05-06	TCRBJ02-05
CASSYSRGGENSPLHF	TCRBV06-05	TCRBJ01-06
CSAREREVTGELFF	TCRBV20	TCRBJ02-02
CASSDINSPLHF	TCRBV27-01	TCRBJ01-06
CASSMGQAYEQYF	TCRBV07-08	TCRBJ02-07
CSARNWDYGYTF	TCRBV20	TCRBJ01-02
CASSVTSSTDTQYF	TCRBV11-03	TCRBJ02-03
CSVESGEGYEQYF	TCRBV29-01	TCRBJ02-07
CAWRTGYNTEAFF	TCRBV30-01	TCRBJ01-01
CSSDTDTQYF	TCRBV29-01	TCRBJ02-03
CASSLERYEKLFF	TCRBV07-02	TCRBJ01-04
CASSLEGRGYYEQYF	TCRBV11-02	TCRBJ02-07
CASSERGPSTDTQYF	TCRBV18-01	TCRBJ02-03
CSARDREKTGELFF	TCRBV20	TCRBJ02-02
CASSSGPGYEQYF	TCRBV07	TCRBJ02-07
CASSQVLAGATDTQYF	TCRBV04-03	TCRBJ02-03
CSARQRDYGYTF	TCRBV20	TCRBJ01-02
CASSPRTGLTDTQYF	TCRBV03-01/03-02	TCRBJ02-03
CSTLTDTQYF	TCRBV29-01	TCRBJ02-03
CSVGLDSTDTQYF	TCRBV29-01	TCRBJ02-03
CASSFKTDQPQHF	TCRBV07-02	TCRBJ01-05
CSAGDGDTEAFF	TCRBV20	TCRBJ01-01
CASSPGPQYEQYF	TCRBV07-06	TCRBJ02-07
CASSPRLPSTDTQYF	TCRBV18-01	TCRBJ02-03
CASSRGPDYEQYF	TCRBV07-06	TCRBJ02-07
CASSPGTGETYEQYF	TCRBV04-01	TCRBJ02-07
CASSEYSEGTEAFF	TCRBV25-01	TCRBJ01-01
CASSLALAGNYNEQFF	TCRBV07-09	TCRBJ02-01
CASSLGGRNTIYF	TCRBV05-01	TCRBJ01-03
CSVGFGEGYEQYF	TCRBV29-01	TCRBJ02-07
CSATRQGPGNTIYF	TCRBV20	TCRBJ01-03
CSASRGEGYTF	TCRBV20-01	TCRBJ01-02
CASSLGPNYEQYV	TCRBV07-06	TCRBJ02-07
CASSDSAGFGYTF	TCRBV02-01	TCRBJ01-02
CASSLGGIGYGYTF	TCRBV27-01	TCRBJ01-02
CASSPTDRNYGYTF	TCRBV04-01	TCRBJ01-02
CSTNTDTQYF	TCRBV29-01	TCRBJ02-03

CASSYGGDPYGYTF	TCRBV06-05	TCRBJ01-02
CASSPGTGEGYEQYF	TCRBV04-01	TCRBJ02-07
CASSLGPYYEQYF	TCRBV07-06	TCRBJ02-07
CASSFPDGTDTQYF	TCRBV28-01	TCRBJ02-03
CSARGTSGGRETQYF	TCRBV20	TCRBJ02-05
CASSLYGYSNQPQHF	TCRBV05-08	TCRBJ01-05
CASSLSYGEQYF	TCRBV07-02	TCRBJ02-07
CASSYGGLPYGYTF	TCRBV27-01	TCRBJ01-02
CASSGGQGEGYEQYF	TCRBV04-01	TCRBJ02-07
CSANRGVGYTF	TCRBV20-01	TCRBJ01-02
CASSANTGGQETQYF	TCRBV05-01	TCRBJ02-05
CASSPYGPSTDTQYF	TCRBV18-01	TCRBJ02-03
CASSVGQRRSYNSPLHF	TCRBV09-01	TCRBJ01-06
CASSYSPQGGYEQYF	TCRBV06-05	TCRBJ02-07
CASSPVGPSTDTQYF	TCRBV18-01	TCRBJ02-03
CSANQGEGYTF	TCRBV20-01	TCRBJ01-02
CASSPSRGSGNTIYF	TCRBV04-01	TCRBJ01-03
CSVGNGEGYEQYF	TCRBV29-01	TCRBJ02-07
CASSLGGWPYGYTF	TCRBV27-01	TCRBJ01-02
CASSLALAGTHNEQFF	TCRBV07-09	TCRBJ02-01
CASSLSRGSGNTIYF	TCRBV04-01	TCRBJ01-03
CASSVQGYSNQPQHF	TCRBV05-08	TCRBJ01-05
CASSSGPPYEQYF	TCRBV07	TCRBJ02-07
CASSLEVNTDTQYF	TCRBV05-01	TCRBJ02-03
CSVEGGAGTDTQYF	TCRBV29-01	TCRBJ02-03
CSARFRDYGYTF	TCRBV20	TCRBJ01-02
CASSLEINTDTQYF	TCRBV05-01	TCRBJ02-03
CSVGRGEGYEQYF	TCRBV29-01	TCRBJ02-07
CASSLGQAYEQYF	TCRBV07-08	TCRBJ02-07
CASSVGASGSLQETQYF	TCRBV09-01	TCRBJ02-05
CAWSWGGEQYF	TCRBV30-01	TCRBJ02-07
CASSQGQGRSYNSPLHF	TCRBV09-01	TCRBJ01-06
CASSLVNAGTEAFF	TCRBV05-01	TCRBJ01-01
CASSQDLRGATDTQYF	TCRBV04-03	TCRBJ02-03
CASSFEGVNIQYF	TCRBV07-02	TCRBJ02-04
CASSLSGRGSYEQYF	TCRBV11-02	TCRBJ02-07
CASSSTSGGRGETQYF	TCRBV05-04	TCRBJ02-05
CASSLEGRGSYEQYF	TCRBV11-02	TCRBJ02-07
CASSLWGDGTDTQYF	TCRBV28-01	TCRBJ02-03
CASSLGGLNYGYTF	TCRBV27-01	TCRBJ01-02
CASSSGPNYEQYV	TCRBV07	TCRBJ02-07

CASSLVNPYEQYF	TCRBV07-02	TCRBJ02-07
CASSLTLAGGVNEQFF	TCRBV05-01	TCRBJ02-01
CASSGYQEGTEAFF	TCRBV25-01	TCRBJ01-01
CASSYGGQPYGYTF	TCRBV06-05	TCRBJ01-02
CASSLALADTYNEQFF	TCRBV07-09	TCRBJ02-01
CASSGGAGVTDTQYF	TCRBV02-01	TCRBJ02-03
CASSAGQGRSYNSPLHF	TCRBV09-01	TCRBJ01-06
CASSLALAGTSNEQFF	TCRBV07-09	TCRBJ02-01
CASSGGQGEQPQHF	TCRBV02-01	TCRBJ01-05
CASSPRGPSTDTQYF	TCRBV18-01	TCRBJ02-03
CASSPRLAGPDTQYF	TCRBV12	TCRBJ02-03
CASSQEDRDTQYF	TCRBV04-03	TCRBJ02-03
CASSRGQGEGYEQYF	TCRBV04-01	TCRBJ02-07
CASSPGAGEGYEQYF	TCRBV04-01	TCRBJ02-07
CASSPRGPNTDTQYF	TCRBV18-01	TCRBJ02-03
CASSQSWGEQYF	TCRBV07-02	TCRBJ02-07
CASSEYQENTEAFF	TCRBV25-01	TCRBJ01-01
CSVGSGEDNEQFF	TCRBV29-01	TCRBJ02-01
CASSLGSNYEQYF	TCRBV07-06	TCRBJ02-07
CASSHSRGSGNTIYF	TCRBV04-01	TCRBJ01-03
CASSTLAGDTGELFF	TCRBV05-01	TCRBJ02-02
CASSPGPNYEQYV	TCRBV07	TCRBJ02-07
CASSRTTNYGYTF	TCRBV07-02	TCRBJ01-02
CASSFEGTNIQYF	TCRBV07-02	TCRBJ02-04
CASSQDNYGYTF	TCRBV11-03	TCRBJ01-02
