# Supplementary material for: T and B cell responses against Epstein–Barr virus in primary sclerosing cholangitis
Source: Nat Med. 2025 Jun 11;31(7):2306–16. doi: 10.1038/s41591-025-03692-w (PMC12283410; doi:10.1038/s41591-025-03692-w)
Supplement: Supplementary file 4 — PSC-associated clonotypes that were targeting EBV antigens as define by the MIRA assay. [file 41591_2025_3692_MOESM4_ESM.pdf]

**CDR3 amino acid**	**V gene segment**	**J gene segment**
CASSTSRGAGNTIYF	TCRBV04-01	TCRBJ01-03
CSVGSGEHYEQYF	TCRBV29-01	TCRBJ02-07
CASSWGQGEGYEQYF	TCRBV04-01	TCRBJ02-07
CASSEGQAYEQYF	TCRBV07-08	TCRBJ02-07
CASSVGQAYEQYF	TCRBV07-08	TCRBJ02-07
CASSQGQGEGYEQYF	TCRBV04-01	TCRBJ02-07
CASSYPRGGENSPLHF	TCRBV06-05	TCRBJ01-06
CSVGAGEGYEQYF	TCRBV29-01	TCRBJ02-07
CASSDLNSPLHF	TCRBV27-01	TCRBJ01-06
CASSPGTGEGYEQYF	TCRBV04-01	TCRBJ02-07
CASSPGVGEGYEQYF	TCRBV04-01	TCRBJ02-07
CASSPGQGEGYEQYF	TCRBV04-01	TCRBJ02-07
CASSLGRDPTGELFF	TCRBV28-01	TCRBJ02-02
CASSRGQGEGYEQYF	TCRBV04-01	TCRBJ02-07
CASSQGPNYEQYF	TCRBV07-06	TCRBJ02-07
CASSSGPNYEQYF	TCRBV07-06	TCRBJ02-07
CASSLGQAYEQYF	TCRBV07-08	TCRBJ02-07
CASSYSRGGENSPLHF	TCRBV06-05	TCRBJ01-06
CSVGSGEDGEQFF	TCRBV29-01	TCRBJ02-01
CASSSGQAYEQYF	TCRBV07-08	TCRBJ02-07
CASSTSRGSGNTIYF	TCRBV04-01	TCRBJ01-03
CSVGTGEGYEQYF	TCRBV29-01	TCRBJ02-07
CSVGQGEGYEQYF	TCRBV29-01	TCRBJ02-07
CASSPVGPSTDTQYF	TCRBV18-01	TCRBJ02-03
CSVGSGEDNEQFF	TCRBV29-01	TCRBJ02-01
CASSPGPNYEQYF	TCRBV07-06	TCRBJ02-07
CASSTGQAYEQYF	TCRBV07-08	TCRBJ02-07
CSVGSGEGAEQYF	TCRBV29-01	TCRBJ02-07
CASSSLNTEAFF	TCRBV27-01	TCRBJ01-01
CASSQGNYGYTF	TCRBV11-03	TCRBJ01-02
CASSPGQGESYEQYF	TCRBV04-01	TCRBJ02-07
CSVGSGEGYEQYF	TCRBV29-01	TCRBJ02-07
